# Supplementary material for: A Moderate Intake of Beer Improves Metabolic Dysfunction-Associated Steatotic Liver Disease (MASLD) in a High-Fat Diet (HFD)-Induced Mouse Model
Source: Molecules. 2024 Dec 17;29(24):5954. doi: 10.3390/molecules29245954 (PMC11676803; doi:10.3390/molecules29245954)
Supplement: Supplementary file 1 [file molecules-29-05954-s001.zip › molecules-3326954-supplementary.pdf]

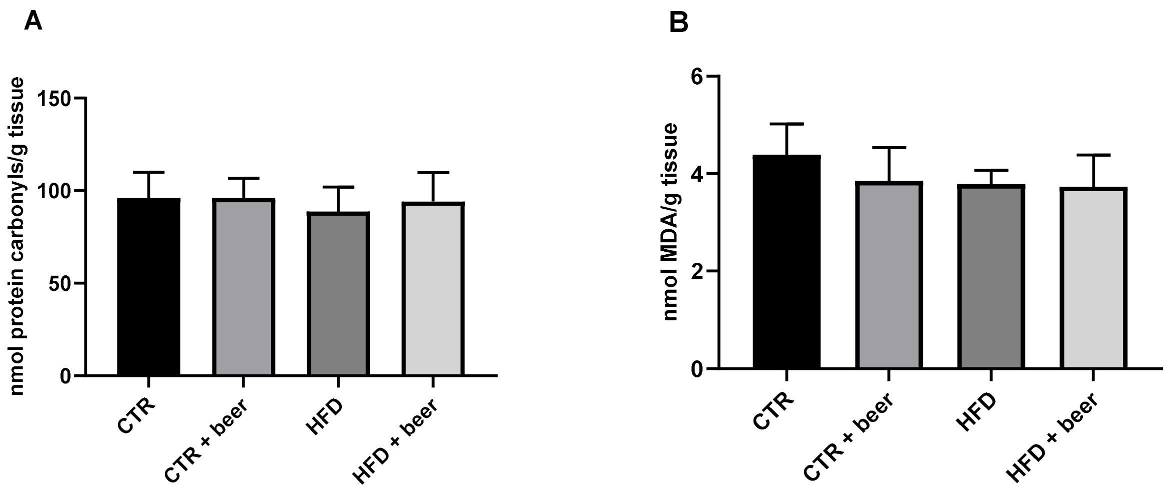

Figure S1. Protein carbonylation (A) and malondialdehyde (MDA) (B) in liver of CTR, CTR+beer, HFD and HFD+beer mouse groups. Values are expressed as means ± SD. Values within each row different letters (a,b) are significantly different by one way ANOVA-test ($p \leq 0.05$).

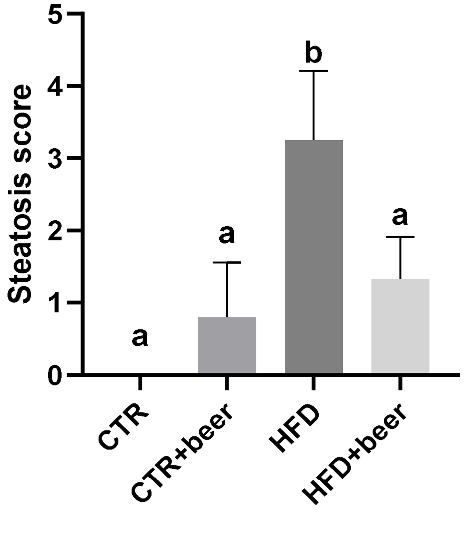

Figure S2: Steatosis scores for mice liver tissues from CTR, CTR+beer, HFD and HFD+beer groups. Values are reported as mean ± SD of relative levels (n=5). Values within each column different letters (a,b) are significantly different by one way ANOVA-test ($p \leq 0.05$), followed by Tukey's post-hoc test.

Table S1. Metabolites concentration (µmol/g) in cecal content of CTR, CTR+beer, HFD, HFD+beer mice.

Group	Formic acid	Malic acid	Uric acid	Succinic acid	Lactic acid	Acetic acid	Propionic acid	Isobutyric acid	Butyric acid	Pyrid[illegible] phosp[illegible]
CTR	0.95^{AB}±1.36	0.82^{A}±0.49	0.01^{A}±0.00	4.22^{B}±1.20	12.80^{A}±4.83	20.59^{A}±5.87	11.33^{A}±4.27	0.72^{A}±0.20	0.46^{A}±0.24	0.09^{A}±[illegible]
CTR+beer	1.96^{A}±0.99	0.70^{A}±0.42	0.01^{A}±0.00	2.59^{C}±0.44	9.32^{A}±3.34	14.71^{B}±3.80	7.68^{AB}±3.56	0.61^{A}±0.15	0.21^{B}±0.04	0.08^{A}±[illegible]
HFD	0.17^{B}±0.14	1.05^{A}±0.30	0.00^{A}±0.00	3.44^{BC}±0.91	1.42^{B}±0.34	2.68^{C}±1.28	4.21^{B}±2.05	0.03^{B}±0.02	0.07^{B}±0.02	0.01^{B}±[illegible]
HFD+beer	0.11^{B}±0.09	1.01^{A}±0.24	0.01^{A}±0.01	5.48^{A}±0.69	2.52^{B}±0.40	3.98^{C}±0.64	7.89^{A}±1.01	0.10^{B}±0.03	0.13^{B}±0.05	0.01^{B}±[illegible]

Values are expressed as means ± SD of three replicates. Values within each row different letters (A, B, C) are significantly different by two-way ANOVA-test ($p \leq 0.05$).

Table S2. Correlation between plasma biochemical parameters and cecal metabolites with the four experimental group

Variables	CTR	CTR+beer	HFD	HFD+beer
AST	0,0074	-0,2309	**0,7056**	**-0,4825**
ALT	-0,0455	**-0,4506**	**0,8575**	-0,3593
Glucose	**-0,6314**	-0,0238	**0,6490**	0,0343
TC	**-0,4046**	**-0,5223**	**0,5818**	0,3631
LDL	-0,3231	**-0,5185**	**0,7684**	0,0876
Urea	-0,3290	0,2669	**0,4216**	-0,3449
Final body weight	**-0,5096**	**-0,3724**	**0,7635**	0,1411
Hepatic lipids	**-0,5086**	-0,2866	**0,8560**	-0,0382
Formic acid	0,0841	**0,5973**	-0,3273	-0,3577
Malic acid	-0,1123	-0,2910	0,2332	0,1750
Uric acid	0,1420	0,2818	**-0,4378**	0,0077
Succinic acid	0,1328	**-0,5777**	-0,2150	**0,6540**
Lactic acid	**0,6740**	0,2633	**-0,5388**	**-0,4285**
Acetic acid	**0,7243**	0,2632	**-0,5538**	**-0,4659**
Propionic acid	**0,5556**	-0,0287	**-0,5391**	-0,0125
Isobutyric acid	**0,6468**	**0,3982**	**-0,5927**	**-0,4810**
Butyric acid	**0,7470**	-0,0516	**-0,4527**	-0,2759
Pyridoxal phosphate	**0,6708**	**0,4004**	**-0,5591**	**-0,5419**
TYR	**0,6422**	-0,0599	**-0,4068**	-0,2042
PHE	**0,6918**	-0,1274	**-0,4634**	-0,1317
TRP	**0,3849**	-0,3382	-0,2934	0,2296

Statistically significant correlations are highlighted in bold ($p<0.05$)

Table S3. RNA-Seq statistics

Name	Group	Total Reads	Mapped Reads	% Mapping
L1	CTR	94819368	85442595	90.11

L2	CTR	75172516	47421179	63.08
L3	CTR	86936722	72067918	82.90
L4	CTR	123475550	105165320	85.17
L5	HFD	86116776	74857814	86.93
L6	HFD	94267858	82823251	87.86
L7	HFD	92676444	82758848	89.30
L8	HFD	92142042	72375705	78.55
L9	CTR+beer	79208334	58273227	73.57
L10	CTR+beer	83004162	74751318	90.06
L11	CTR+beer	118033700	90108392	76.34
L12	CTR+beer	91538744	80417817	87.85
L13	HFD+beer	99425388	86220323	86.72
L14	HFD+beer	88011176	78869264	89.61
L15	HFD+beer	77866386	69025429	88.65
L16	HFD+beer	81171884	71506428	88.09

Table S4. Differentially expressed genes DEGs calculated between: CTR vs HFD, CTR vs HFD+beer, CTR vs CTR+beer, HFD vs HFD+beer. For each comparison: logarithmic Fold Change (logFC), logarithmic count per million (logCPM), Pvalue and False Discovery Rate (FDR) was reported.

**CTR vs HFD**				
**Gene**	**logFC**	**logCPM**	**PValue**	**FDR**
Adam11	2.716369	4.257475	5.97E-20	1.12E-15
Cyp2b10	4.4495	4.590597	1.88E-19	1.76E-15
Slc39a4	2.136319	3.801845	1.11E-16	6.92E-13
Fabp5	2.576208	3.056301	3.56E-16	1.67E-12
Cyp2c55	3.874664	2.287299	3.43E-15	1.29E-11
Mfsd2a	-3.27247	4.17009	1.46E-11	4.55E-08
Tuba8	3.278292	0.323603	3.21E-11	8.60E-08
1810055G02Rik	-1.62031	3.783062	9.15E-11	2.14E-07
Rgs16	-3.75713	3.217859	1.09E-10	2.27E-07
Grip1	-3.1765	0.923688	1.59E-10	2.98E-07
Ifitm2	1.546896	4.580946	3.82E-09	6.50E-06
Snhg11	1.684267	4.809192	4.16E-09	6.50E-06
Klhl31	-3.90173	-0.71509	5.20E-09	7.49E-06
Dnajc12	3.434754	2.157957	1.99E-08	2.53E-05
Gm31121	-4.63216	-1.00291	2.03E-08	2.53E-05
Ugt1a9	2.307086	3.158467	2.70E-08	3.14E-05
Gstm3	2.784718	2.636442	2.85E-08	3.14E-05

Clvs1	6.87447	-1.67863	3.18E-08	3.18E-05
Rnu1b6	-8.3312	-0.42052	3.25E-08	3.18E-05
Gm2436	7.339101	-1.26028	3.40E-08	3.18E-05
1810053B23Rik	-3.71489	-0.0458	5.64E-08	5.03E-05
Orm2	5.558596	6.452298	6.74E-08	5.58E-05
Saa1	6.079548	9.723102	6.85E-08	5.58E-05
Saa2	7.336037	9.307076	1.20E-07	9.40E-05
Fgf21	-3.52854	0.475714	2.66E-07	0.000199
Lcn2	6.606444	6.354745	3.44E-07	0.000248
Gm33799	-5.11364	-1.75126	4.03E-07	0.00028
Apcs	2.512056	6.010405	4.22E-07	0.000283
Krt23	-2.78933	-0.13284	4.58E-07	0.000296
Rfx4	-1.44455	1.845696	5.30E-07	0.000331
Klf10	-1.36412	3.474219	5.98E-07	0.000362
Saa3	5.628572	3.945826	7.87E-07	0.000461
LOC118568368	-2.6199	1.356757	9.08E-07	0.000515
n-R5s118	6.884492	-1.6996	9.79E-07	0.00054
Arrdc3	-1.83604	6.495846	1.07E-06	0.000571
Cetn1	-3.6902	-0.98526	1.25E-06	0.000643
Snord3b4	1.684074	4.605238	1.27E-06	0.000643
Trhde	-1.56752	2.063819	1.35E-06	0.000668
Cyp2c53-ps	2.614997	2.260957	1.57E-06	0.000754
LOC118567337	-1.60302	1.456489	1.69E-06	0.000787
Htatip2	1.500633	1.919125	1.72E-06	0.000787
Htd2	6.786708	-1.76455	3.30E-06	0.001471
Gm33944	-4.59795	-0.14545	3.40E-06	0.001484
Ugdh	1.157386	5.415962	3.87E-06	0.001649
Gm32063	-1.15839	5.116926	4.06E-06	0.001693
Impg1	-4.50198	-1.80916	4.52E-06	0.001842
Reep5	1.361865	1.81789	5.17E-06	0.002028
Fgl1	2.535108	7.56863	5.24E-06	0.002028
Pisd-ps1	1.065768	3.271778	5.30E-06	0.002028
Esco2	2.200331	0.453213	5.47E-06	0.002052
Cyp17a1	-1.85628	0.825695	6.19E-06	0.002275
Gadd45b	2.697138	-0.77748	6.40E-06	0.002288
Tgtp2	-1.34891	2.155695	6.47E-06	0.002288
Teddm1a	-3.82404	-1.1233	6.86E-06	0.002381
BC024139	-1.09945	2.611268	7.42E-06	0.002528
Akr1c19	1.155794	3.48584	8.27E-06	0.002768
Mup-ps12	-1.26906	3.964271	9.19E-06	0.003021
Plekhb1	1.31326	2.395886	9.69E-06	0.003132
Prtn3	4.541231	0.215595	1.10E-05	0.003485
Gm34119	-2.27877	0.575117	1.13E-05	0.003545
Ifit3	-1.03789	3.014033	1.19E-05	0.003656
Orm1	2.045096	7.483176	1.37E-05	0.004131
Ffar4	-4.04298	-1.90181	1.46E-05	0.004357
Gm19619	-1.99551	1.108024	1.54E-05	0.004514

Fam25a	1.630983	4.85127	1.74E-05	0.005027
Mt2	5.847931	4.642904	2.19E-05	0.006211
Fads2	-1.29709	7.965092	2.42E-05	0.006772
Gm30536	-1.36845	1.914126	2.75E-05	0.007583
Isyna1	2.545873	2.322248	2.95E-05	0.008023
Stap1	-2.04808	-0.0528	3.01E-05	0.008048
1810064F22Rik	2.396391	0.687624	3.16E-05	0.008339
Csad	2.132388	6.246884	3.49E-05	0.009096
Prxl2b	1.720635	0.200024	3.60E-05	0.009251
Lrtm1	-1.55825	0.984265	3.66E-05	0.009276
Rgsl1	-5.5768	-2.56306	3.86E-05	0.009644
Atp6v0c-ps2	1.597049	2.219994	3.93E-05	0.009694
A930038B10Rik	-1.41089	1.455208	4.07E-05	0.009894
Gm42154	-2.16494	-0.55288	4.12E-05	0.009894
Gm29906	5.486488	-2.61772	4.92E-05	0.011541
Mvp	1.050782	4.43082	4.96E-05	0.011541
Etfbkmt	1.29237	4.642548	4.99E-05	0.011541
Zc3h6	-1.11904	3.325275	5.06E-05	0.011555
Cyp2c29	1.937853	9.258688	5.38E-05	0.01211
Gm48551	5.43952	-2.67466	5.50E-05	0.01211
Nav2	-1.21034	7.452001	5.52E-05	0.01211
Adgrv1	-2.26461	-0.13679	5.56E-05	0.01211
Cadm4	1.601932	1.706939	5.62E-05	0.01211
Pcp4l1	1.464118	0.982651	5.71E-05	0.01216
Ttll2	-5.53219	-2.59679	5.89E-05	0.012409
Synj2	-1.21361	4.033306	6.37E-05	0.013263
Mt1	4.821521	5.369455	7.05E-05	0.014512
Gpr12	-1.50377	1.247985	8.15E-05	0.016503
LOC118568404	1.750939	0.698507	8.26E-05	0.016503
Cd40lg	-5.31635	-2.70061	8.28E-05	0.016503
Gm42226	1.487572	0.786258	8.80E-05	0.017356
9530057J20Rik	-4.29116	-2.25506	8.97E-05	0.017503
Epha2	-1.11224	4.835061	0.000103	0.019838
Gm30246	-3.85213	-1.95269	0.000105	0.020032
Gna14	-1.61119	1.642372	0.000107	0.020136
Serpina3g	-1.52254	1.222129	0.000107	0.020136
Gm48552	-5.33364	-2.65313	0.000116	0.021526
Gm2446	-3.24048	-0.64556	0.000123	0.022571
Gbp3	-1.09035	2.022031	0.000124	0.022617
BC023105	-2.51479	3.858003	0.000127	0.022834
Rps14	0.942941	3.332168	0.00014	0.025067
Gm33721	1.975007	1.561573	0.000143	0.02522
Galk1	1.020539	2.771814	0.000146	0.025468
Cd300ld5	-6.04491	-2.33687	0.000149	0.025468
Gm53846	-2.38388	0.130805	0.000149	0.025468
Vtcn1	1.87261	0.51433	0.000149	0.025468
Gm26684	-1.39801	2.128918	0.000158	0.026574

Tlr6	-3.63189	-2.01143	0.000159	0.026574
Lmod3	-4.31027	-2.19135	0.00016	0.026574
Gm2a	1.087705	4.648487	0.000169	0.027805
Ccdc141	-1.16771	5.322445	0.000182	0.029726
Gm41358	-0.94735	2.557491	0.00019	0.030312
Samd9l	-0.92186	4.427605	0.00019	0.030312
Lrg1	2.123767	8.763855	0.000191	0.030312
Nr1d1	-1.74562	6.724662	0.000193	0.030328
Zfp608	-1.00879	3.781779	0.000197	0.030743
Cry1	1.475324	1.660525	0.0002	0.030907
Cyp7a1	1.185798	6.08801	0.000213	0.032661
Gm15772	1.197385	1.895959	0.000228	0.034246
Gm51644	-2.19231	0.403673	0.000228	0.034246
Marcksl1-ps4	1.574482	1.567108	0.000228	0.034246
Gm29669	-2.64476	-1.32988	0.000232	0.034552
Cpb2	1.050913	5.825338	0.000242	0.035075
Tex35	-3.6504	-2.23311	0.000243	0.035075
Tmem176b	1.102738	5.871959	0.000243	0.035075
Qsox1	1.187682	6.647667	0.000246	0.035075
Slco2a1	0.874217	4.514583	0.000246	0.035075
Sox30	-4.94707	-2.88101	0.000247	0.035075
Rpl13	0.892861	4.269999	0.000256	0.035968
Gm15998	1.470432	3.724801	0.000259	0.035968
Hp	1.933894	10.17951	0.000259	0.035968
Nrros	-0.85697	3.166181	0.000264	0.036399
Kcng3	-4.62563	-2.51683	0.000281	0.03839
Gm53265	-1.1456	1.671799	0.000288	0.039062
Chrna9	4.209907	-2.41686	0.000293	0.039484
Cyp3a11	2.267406	9.922794	0.000298	0.039883
Gm39398	-2.55158	-1.17145	0.000308	0.04098
Pcgf2	1.000263	3.091452	0.000319	0.042088
Gm15501	1.939546	-0.54981	0.000322	0.042088
Capn12	-2.98513	-1.62252	0.000327	0.042088
Cyp26a1	1.711245	1.652521	0.000327	0.042088
Hpx	1.914348	10.17808	0.000328	0.042088
Gstp-ps	1.373167	4.605955	0.000348	0.044348
Fut1	-4.93874	-2.89621	0.000367	0.046432
Gsta2	1.660816	2.050858	0.000376	0.047253
Fam13a	-1.03391	4.162831	0.000378	0.047253
Rab11fip2	-0.93124	3.286621	0.000387	0.04748
Slc35g1	1.183331	1.404803	0.000388	0.04748
Gm33548	2.153653	1.247302	0.000392	0.04748
Gm32894	2.739917	1.614082	0.000393	0.04748
Rplp1	0.884116	4.277753	0.000393	0.04748
Ndufa2	1.404149	2.537539	0.000395	0.04748
Gbp10	-1.4911	3.744822	0.000399	0.047665
0610043K17Rik	-1.45313	2.379355	0.000406	0.048139

Syt14	-3.63195	-1.37955	0.000416	0.048995
Gm16016	-1.27664	0.761525	0.00042	0.049193
Rpusd3	0.948423	2.308956	0.000429	0.049885
Fam222a	1.547727	2.217483	0.000431	0.049885
**CTR vs HFD+beer**				
**Gene**	**logFC**	**logCPM**	**PValue**	**FDR**
Gm26444	11.5088	2.650138	1.35E-13	2.55E-09
Fam220a	8.758821	-0.10093	1.64E-10	1.55E-06
Grip1	-2.78921	0.752455	5.63E-07	0.00295
Pnp2	4.088909	0.283821	6.27E-07	0.00295
Hbb-bs	1.889345	4.733797	1.09E-06	0.004111
Orm2	5.286442	6.599132	2.08E-06	0.006519
Fam25a	1.670943	4.98858	4.59E-06	0.012336
Saa3	5.122075	4.095292	5.46E-06	0.012844
Marcksl1-ps4	1.948663	1.620719	8.59E-06	0.015564
Rgs16	-4.30407	3.887139	9.45E-06	0.015564
Dnajc12	2.676997	2.375373	1.19E-05	0.015564
Teddm1a	-3.8722	-0.89556	1.29E-05	0.015564
Gm32894	3.512108	1.662474	1.34E-05	0.015564
Rnu2-10	1.894124	4.119262	1.38E-05	0.015564
Apcs	1.975008	6.24859	1.45E-05	0.015564
Gm31121	-4.49815	-0.98792	1.50E-05	0.015564
Isyna1	2.606553	2.448436	1.51E-05	0.015564
Gm51464	2.173858	1.142959	1.59E-05	0.015564
Gm42221	2.594137	2.287184	1.62E-05	0.015564
Snora81	2.027831	3.946067	1.65E-05	0.015564
Saa1	5.076769	9.885243	1.78E-05	0.015988
Gm23444	1.545454	2.757775	2.00E-05	0.016425
Rnu12	1.458835	5.534607	2.01E-05	0.016425
Fgf21	-2.98286	0.152331	2.19E-05	0.016879
Lcn2	5.701665	6.507689	2.24E-05	0.016879
Mfsd2a	-2.412	3.570183	2.37E-05	0.017128
Saa2	6.105538	9.459775	2.61E-05	0.017907
1810055G02Rik	-1.41437	3.780876	2.66E-05	0.017907
BC023105	-1.6181	3.288743	3.28E-05	0.021308
Gm25939	1.899939	1.755937	3.53E-05	0.022181
Rtl1	2.394236	1.548371	3.67E-05	0.022291
Ifitm2	1.348876	4.781822	3.86E-05	0.022721
Gck	-1.43136	6.20119	4.63E-05	0.025762
Orm1	2.030477	7.630437	4.70E-05	0.025762
Mir6538	2.35211	1.694933	4.79E-05	0.025762
Spr	1.131839	3.351779	5.06E-05	0.026484
Gm51616	-2.13054	1.508584	6.13E-05	0.030688
Gbp10	-1.32601	3.775622	6.19E-05	0.030688
Fgl1	2.280874	7.752541	6.44E-05	0.030865
Adgrv1	-2.61953	0.298093	6.56E-05	0.030865
Gpr12	-1.51841	1.409702	7.20E-05	0.032467

Cc2d2b	-2.45015	0.006191	7.24E-05	0.032467
Gvin-ps2	-1.61582	4.87381	0.000104	0.045592
**CTR vs CTR+beer**				
**Gene**	**logFC**	**logCPM**	**PValue**	**FDR**
Gm2436	7.290398	-1.26115	4.84E-08	0.000627
Saa3	6.527595	3.90079	8.45E-08	0.000627
Nfil3	1.475431	4.322221	1.02E-07	0.000627
H1f1	1.604702	1.853006	2.34E-07	0.001081
Gstp-ps	1.06813	4.674989	6.03E-07	0.002225
mt-Rnr1	-2.08504	9.846488	1.62E-06	0.004971
8030431J09Rik	1.587098	4.072629	2.49E-06	0.006087
Gm33548	3.032276	1.098065	2.64E-06	0.006087
Rgs16	-3.87711	3.30726	3.59E-06	0.007366
Slco1a4	-1.04777	4.40516	1.26E-05	0.022568
Gm32894	3.582247	1.50384	1.36E-05	0.022568
Tnfrsf13c	-3.9188	-2.00914	1.47E-05	0.022568
Gys2	-1.39533	6.582525	1.63E-05	0.023182
Gm15441	-1.84494	0.667882	1.90E-05	0.024558
1810055G02Rik	-1.08769	3.379866	2.00E-05	0.024558
Gm10804	-2.73669	0.96854	3.99E-05	0.046013
Gm39795	2.403287	-1.0794	4.49E-05	0.048614
Gm826	-1.52315	2.068982	4.74E-05	0.048614
**HFD vs HFD+beer**				
**Gene**	**logFC**	**logCPM**	**PValue**	**FDR**
n-TGgcc6	-8.53017	-0.17373	2.68E-20	5.11E-16
Gm26444	12.42561	3.557455	3.51E-13	3.34E-09
Fam220a	8.31502	-0.50934	4.86E-10	3.09E-06
Gm2436	-7.76401	-0.82971	3.73E-09	1.78E-05
n-TEttc6	7.568593	-1.1921	2.05E-08	7.81E-05
Nr4a1	3.151595	3.924963	5.87E-07	0.001867
Fos	1.447462	2.454999	7.24E-07	0.001973
Gm51464	2.515807	1.484409	1.07E-06	0.002551
Gm40616	1.188982	4.406088	7.61E-06	0.016121
Cldn34c1	1.420528	4.779805	1.67E-05	0.031879
Gm26799	-2.07567	0.176505	1.91E-05	0.033128
Cd300ld5	6.242457	-2.29697	2.14E-05	0.033987
9530057J20Rik	3.885588	-2.17812	2.94E-05	0.043138

Table S5. Gene Ontology (GO) analysis the differentially expressed genes DEGs for CTR*vs*HFD and CTR*vs*HFD+beer comparisons. Gene ontology IDs (GO-ID), gene ontology terms (GO-term), associated genes found and corrected (Bonferroni step down) p-values as determined by ClueGO (http://apps.cytoscape.org/apps/cluego) are indicated.

CTR*vs*HFD			
**ID GO:**	**Associated Genes Found**	**Term**	**PValue**
0001562	Gbp10, Gbp3, Vtcn1	response to protozoan	2.1E-02
0001774	Nr1d1, Stap1, Tlr6	microglial cell activation	2.1E-02
0002269	Nr1d1, Stap1, Tlr6	leukocyte activation involved in inflammatory response	2.1E-02
0002526	Hp, Orm1, Orm2, Saa1, Saa2, Saa3	acute inflammatory response	2.0E-03
0006636	Fabp5, Fads2, Prxl2b	unsaturated fatty acid biosynthetic process	2.1E-02
0006805	Cyp26a1, Cyp2b10, Cyp2c29, Cyp2c55, Gsta2	xenobiotic metabolic process	8.9E-03
0006882	Mt1, Mt2, Slc39a4	cellular zinc ion homeostasis	8.9E-03
0006953	Hp, Orm1, Orm2, Saa1, Saa2, Saa3	acute-phase response	1.4E-05
0010893	Cyp17a1, Cyp7a1, Nr1d1	positive regulation of steroid biosynthetic process	1.1E-02
0019217	Cyp7a1, Etfbkmt, Fabp5, Galk1, Mfsd2a	regulation of fatty acid metabolic process	6.0E-03
0019373	Cyp2b10, Cyp2c29, Cyp2c55	epoxygenase P450 pathway	1.3E-02
0035456	Gbp3, Ifit3, Ifitm2, Tgtp2	response to interferon-beta	9.3E-03
0035458	Gbp3, Ifit3, Ifitm2, Tgtp2	cellular response to interferon-beta	6.0E-03
0035634	Fgl1, Gsta2, Ifit3, Saa1, Saa2, Saa3	response to stilbenoid	1.7E-07
0043030	Nr1d1, Stap1, Tlr6	regulation of macrophage activation	2.3E-02
0045744	Arrdc3, Cry1, Rgs16	negative regulation of G protein-coupled receptor signaling pathway	2.0E-02
0045922	Cyp7a1, Etfbkmt, Mfsd2a	negative regulation of fatty acid metabolic process	1.1E-02
0045940	Cyp17a1, Cyp7a1, Nr1d1	positive regulation of steroid metabolic process	2.2E-02
0046164	Cyp7a1, Galk1, Synj2	alcohol catabolic process	2.1E-02
0046916	Hpx, Lcn2, Mt1, Mt2, Slc39a4	cellular transition metal ion homeostasis	7.0E-03
0048246	Saa1, Saa2, Stap1	macrophage chemotaxis	2.1E-02
0050994	Etfbkmt, Fgf21, Mfsd2a	regulation of lipid catabolic process	1.2E-02
0055069	Mt1, Mt2, Slc39a4	zinc ion homeostasis	1.0E-02
0061900	Nr1d1, Stap1, Tlr6	glial cell activation	2.0E-02
0097028	Cd40lg, Prtn3, Tmem176b	dendritic cell differentiation	2.1E-02
0120161	Arrdc3, Fabp5, Ffar4, Fgf21, Lcn2, Nr1d1	regulation of cold-induced thermogenesis	3.2E-03
0120162	Fabp5, Ffar4, Fgf21, Lcn2	positive regulation of cold-induced thermogenesis	2.2E-02
0150076	Nr1d1, Stap1, Tlr6	neuroinflammatory response	7.4E-03
1905517	Saa1, Saa2, Stap1	macrophage migration	1.8E-02
**CTR*vs*HFD+beer**			
**ID GO:**	**Associated Genes Found**	**Term**	**Pvalue**
0006953	Orm1, Orm2, Saa1, Saa2, Saa3	acute-phase response	1.5E-08
0035634	Fgl1, Saa1, Saa2, Saa3	response to stilbenoid	3.7E-08

Table S6. RRBS statistics.

Sample Name	Group	M Seqs	M C's	% Mapping	% mCpG
L1	CTR	52.1	1281.2	72.5	50.4
L2	CTR	48.3	1136.6	67.5	49.5
L3	CTR	53.4	1275.7	70.6	49.4
L4	CTR	59.3	1344.9	68.3	45.9
L5	HFD	51.4	1071.6	68.3	48.2

L6	HFD	69.7	1614.1	69.8	45.0
L7	HFD	48.9	1232.3	71.6	50.9
L8	HFD	51.4	1107.2	68.7	44.6
L9	CTR+beer	65.4	1678.5	72.1	46.0
L10	CTR+beer	58.9	1427.7	73.5	45.3
L11	CTR+beer	72.7	1891.2	76.2	51.6
L12	CTR+beer	98.0	2344.6	73.0	43.6
L13	HFD+beer	54.7	1249.1	70.5	40.2
L14	HFD+beer	97.3	2130.8	68.7	38.4
L15	HFD+beer	88.6	1939.9	68.6	44.1
L16	HFD+beer	43.6	1005.2	70.3	45.0

Table S7. Differentially Methylated Cytosine DMCs calculated between: CTR vs HFD, CTR vs HFD+beer, CTR vs CTR+beer, HFD vs HFD+beer. For each comparison: Chromosome, Position, Gene, False Discovery Rate (Logistic regression $p<0.05$ after correction. Min obs 10 (FDR) and methylation differences was reported.

**CTR vs HFD**				
**Chromosome**	**Position**	**Gene**	**FDR**	**Meth Difference**
NC_000067.7	22338972	null	4.09E-11	-23.2036
NC_000067.7	34217150	Dst	6.48E-08	-11.9897
NC_000067.7	34278984	Dst	8.19E-08	18.56758
NC_000067.7	34278993	Dst	4.2E-12	24.6533
NC_000067.7	34478661	Ccdc115	2.95E-08	-10.0792
NC_000067.7	52541058	Nab1	5.27E-10	32.09332
NC_000067.7	55143062	null	2.16E-07	-24.2114
NC_000067.7	59523730	Fzd7	1.48E-11	-38.6176
NC_000067.7	59523744	Fzd7	3.14E-08	-31.8494
NC_000067.7	59523831	Fzd7	1.2E-09	-35.1017
NC_000067.7	1.37E+08	null	6.66E-07	-18.0793
NC_000067.7	1.53E+08	Npl	5.37E-09	21.44641
NC_000067.7	1.72E+08	Kcnj10	8.6E-11	-17.2131
NC_000067.7	1.8E+08	null	2.31E-07	-10.5947
NC_000067.7	1.81E+08	null	4.63E-09	-12.8785
NC_000067.7	1.83E+08	null	9.09E-08	-21.1506
NC_000067.7	1.85E+08	Mtarc2	4.07E-07	-31.8068
NC_000067.7	1.87E+08	Gpatch2	2.22E-07	21.90664
NC_000067.7	1.87E+08	Gpatch2	2.3E-07	21.13245
NC_000068.8	11727700	Il15ra	6.73E-08	27.2771
NC_000068.8	20464054	Etl4	4.92E-07	-19.8334
NC_000068.8	20904949	Arhgap21	1.77E-11	-29.5902
NC_000068.8	20904962	Arhgap21	5.92E-09	-23.1676
NC_000068.8	20905005	Arhgap21	3.8E-09	-25.7168
NC_000068.8	27575345	Rxra	1.34E-08	12.46991
NC_000068.8	32385198	null	2.96E-07	-36.2573
NC_000068.8	33526437	Lmx1b	5.92E-07	33.18217
NC_000068.8	52269071	Gm13522	4.84E-09	-29.5585

NC_000068.8	73446869	Chn1	2.29E-13	18.27319
NC_000068.8	73446872	Chn1	1.35E-09	14.83787
NC_000068.8	73950500	null	2.14E-07	-37.607
NC_000068.8	75899873	Pde11a	8.01E-08	-15.3677
NC_000068.8	84713172	Rtn4rl2	9.28E-08	-35.1972
NC_000068.8	1.05E+08	Wt1os	1.03E-08	32.88914
NC_000068.8	1.09E+08	null	6.11E-07	-19.0668
NC_000068.8	1.21E+08	Map1a	5.91E-07	-23.0025
NC_000068.8	1.22E+08	Gm34325	5.59E-08	-40.1797
NC_000068.8	1.52E+08	Rem1	7.57E-07	31.47436
NC_000068.8	1.57E+08	Blcap	8.51E-08	-15.3878
NC_000068.8	1.6E+08	Gm34980	3.57E-07	-11.8267
NC_000068.8	1.63E+08	null	3.75E-09	-19.5706
NC_000068.8	1.63E+08	L3mbtl1	8.52E-09	-14.4292
NC_000068.8	1.65E+08	null	8.71E-09	-27.9889
NC_000068.8	1.65E+08	null	5.34E-07	-24.1131
NC_000068.8	1.66E+08	Zmynd8	9.49E-07	-24.6021
NC_000068.8	1.68E+08	A530013C23Rik	2.15E-09	-21.8743
NC_000068.8	1.73E+08	null	7.67E-12	-38.092
NC_000068.8	1.78E+08	null	1.65E-07	-10.1397
NC_000069.7	36519821	Anxa5	1.34E-08	-26.9542
NC_000069.7	51469240	5031434O11Rik	1.32E-09	-33.8052
NC_000069.7	51952497	Maml3	4.89E-08	-15.3732
NC_000069.7	55264348	Dclk1	2.26E-08	-25.6559
NC_000069.7	67369961	Gfm1	2.64E-07	13.90026
NC_000069.7	79286719	Gm17359	1.92E-10	21.26921
NC_000069.7	79613976	Rxfp1	5.33E-08	-23.9439
NC_000069.7	79634114	Rxfp1	1.56E-09	27.84442
NC_000069.7	82920185	Fgg	1.21E-08	-24.0674
NC_000069.7	82920221	Fgg	1.39E-07	-22.716
NC_000069.7	89706854	null	1.11E-09	-23.8743
NC_000069.7	89706914	null	7.54E-07	-21.4135
NC_000069.7	96297847	null	2.39E-07	-18.9448
NC_000069.7	1.29E+08	Elovl6	1.73E-08	-20.6062
NC_000069.7	1.31E+08	Lef1	1.87E-11	30.09499
NC_000069.7	1.31E+08	Lef1	8.56E-08	28.53374
NC_000069.7	1.31E+08	Lef1	6.02E-14	34.225
NC_000069.7	1.42E+08	Kyat3	1.19E-07	-15.8807
NC_000070.7	40261373	Topors	2.03E-10	-11.164
NC_000070.7	82455682	Gm11266	2.82E-07	-21.5742
NC_000070.7	82455717	Gm11266	1.15E-10	-51.0406
NC_000070.7	97991130	Nfia	9.65E-08	15.50323
NC_000070.7	1.2E+08	Hivep3	1.04E-07	32.38343
NC_000070.7	1.36E+08	Asap3	8.58E-13	24.92185
NC_000070.7	1.36E+08	null	3.01E-08	-32.4974
NC_000070.7	1.37E+08	null	4.32E-09	19.78396
NC_000070.7	1.4E+08	Klhdc7a	6.91E-07	-11.326

NC_000070.7	1.41E+08	null	5.95E-18	-13.6987
NC_000070.7	1.42E+08	Efhd2	5.84E-07	-35.0093
NC_000070.7	1.42E+08	Efhd2	1.05E-08	-38.553
NC_000070.7	1.42E+08	Efhd2	6.34E-08	-37.5582
NC_000070.7	1.46E+08	null	3.57E-08	-15.8172
NC_000070.7	1.46E+08	null	2.79E-10	-17.4561
NC_000070.7	1.46E+08	null	7.45E-08	-14.9803
NC_000070.7	1.46E+08	null	8.51E-09	-15.636
NC_000070.7	1.46E+08	null	3.16E-11	-18.4649
NC_000070.7	1.46E+08	null	3.82E-09	-16.199
NC_000070.7	1.46E+08	null	5.55E-14	-20.7522
NC_000070.7	1.46E+08	null	1.14E-09	-16.1337
NC_000070.7	1.46E+08	null	8.51E-15	-21.2343
NC_000070.7	1.46E+08	null	2.49E-09	-16.7364
NC_000070.7	1.46E+08	null	4.82E-07	-20.4735
NC_000070.7	1.46E+08	null	3.94E-07	-16.5675
NC_000070.7	1.46E+08	null	5.53E-09	-24.6448
NC_000070.7	1.46E+08	null	7.14E-07	-18.16
NC_000070.7	1.46E+08	null	7.78E-07	-20.0638
NC_000070.7	1.49E+08	Mtor	2.79E-07	-11.8494
NC_000071.7	28287357	Gm35223	6.19E-07	-17.6692
NC_000071.7	38938142	Clnk	8.62E-07	-17.9498
NC_000071.7	50191338	Adgra3	1.39E-08	-15.128
NC_000071.7	50191371	Adgra3	1.23E-12	-19.9956
NC_000071.7	50191402	Adgra3	8.52E-07	-12.9863
NC_000071.7	55131854	null	2.58E-12	56.08122
NC_000071.7	66372423	null	1.15E-09	-13.7544
NC_000071.7	67855298	Atp8a1	5.94E-11	-28.9376
NC_000071.7	1.02E+08	null	6.81E-09	20.1384
NC_000071.7	1.04E+08	Aff1	6.61E-07	30.209
NC_000071.7	1.05E+08	Pkd2	1.98E-11	16.0272
NC_000071.7	1.05E+08	Pkd2	1.23E-10	14.72828
NC_000071.7	1.06E+08	Lrrc8c	9.91E-08	-34.873
NC_000071.7	1.2E+08	null	1.88E-12	-29.9693
NC_000071.7	1.23E+08	Rhof	2.23E-09	-18.0579
NC_000071.7	1.24E+08	Vps37b	5.31E-07	18.1397
NC_000071.7	1.24E+08	Vps37b	3.62E-07	25.84763
NC_000071.7	1.25E+08	Rilpl1	9.12E-09	-28.8216
NC_000071.7	1.25E+08	n-TAtgc10	2.47E-17	-23.7489
NC_000071.7	1.25E+08	n-TAtgc10	8.02E-17	-24.7484
NC_000071.7	1.29E+08	Rimbp2	6.9E-08	-30.7125
NC_000071.7	1.36E+08	Hspb1	4.49E-07	-14.9732
NC_000071.7	1.36E+08	Hspb1	1.45E-09	-36.2323
NC_000071.7	1.44E+08	Eif2ak1	9.21E-08	-11.6862
NC_000071.7	1.44E+08	Eif2ak1	7.62E-12	-14.006
NC_000071.7	1.44E+08	2900089D17Rik	9.96E-09	-20.8411
NC_000071.7	1.46E+08	Cdk8	2.71E-09	11.14254

NC_000071.7	1.46E+08	Cdk8	1.12E-20	16.58661
NC_000072.7	23235916	null	8.8E-09	32.2998
NC_000072.7	28420954	Gcc1	1.41E-07	-16.8029
NC_000072.7	28421007	Gcc1	4.64E-07	-14.8657
NC_000072.7	28928138	Snd1	9.8E-09	-18.1113
NC_000072.7	28928187	Snd1	1.18E-09	-19.1596
NC_000072.7	28928202	Snd1	4.07E-07	-15.2579
NC_000072.7	31713335	Gm13846	6.62E-11	-31.1064
NC_000072.7	34974714	null	7.64E-09	-20.1675
NC_000072.7	38234484	null	1.06E-08	12.19691
NC_000072.7	47725420	Gm52861	2.95E-08	-12.4168
NC_000072.7	87473091	Arhgap25	8.01E-09	30.11771
NC_000072.7	90439670	Klf15	2.78E-07	-20.5188
NC_000072.7	90439676	Klf15	7.44E-08	-22.0758
NC_000072.7	90439684	Klf15	2.76E-07	-19.2562
NC_000072.7	90439686	Klf15	2.19E-07	-21.1829
NC_000072.7	90439693	Klf15	7.44E-08	-22.0758
NC_000072.7	90439696	Klf15	8.48E-08	-23.5545
NC_000072.7	90439728	Klf15	9.02E-07	-18.9492
NC_000072.7	90439730	Klf15	1.29E-07	-20.6378
NC_000072.7	90439734	Klf15	4.08E-08	-22.2973
NC_000072.7	90439736	Klf15	1.29E-07	-20.6378
NC_000072.7	90439774	Klf15	3.78E-08	-21.8878
NC_000072.7	90439778	Klf15	3.78E-08	-21.8878
NC_000072.7	90439794	Klf15	1.47E-08	-23.318
NC_000072.7	1.15E+08	Syn2	1.02E-08	-24.2341
NC_000072.7	1.24E+08	null	1.48E-08	30.08542
NC_000072.7	1.25E+08	Atn1	2.36E-07	-25.6123
NC_000072.7	1.43E+08	C2cd5	3.77E-07	-23.9269
NC_000072.7	1.45E+08	Lmntd1	4.66E-09	11.19144
NC_000072.7	1.45E+08	Lmntd1	1.85E-07	-39.2914
NC_000072.7	1.49E+08	Amn1	2.32E-07	-38.7936
NC_000072.7	1.49E+08	Amn1	2.32E-07	-38.7936
NC_000073.7	3267939	Nlrp12	7.33E-07	-17.4126
NC_000073.7	18482760	null	7.6E-07	-22.7286
NC_000073.7	18482845	null	2.22E-07	-24.3395
NC_000073.7	28078631	Paf1	5.16E-09	-11.9522
NC_000073.7	28078653	Paf1	1.64E-08	-11.408
NC_000073.7	28078660	Paf1	2.74E-15	-15.859
NC_000073.7	28078715	Paf1	1.13E-07	-11.0073
NC_000073.7	30811144	Hpn	1.86E-07	-10.5147
NC_000073.7	46486859	null	3.72E-11	31.66536
NC_000073.7	48992593	Nav2	1.9E-10	-29.939
NC_000073.7	51160070	Gm33190	2.12E-09	33.18459
NC_000073.7	67101455	null	9.02E-07	18.59065
NC_000073.7	99000635	Serpinh1	8.09E-14	-46.0288
NC_000073.7	1.04E+08	null	1.79E-07	-23.4043

NC_000073.7	1.26E+08	Sbk1	3.18E-09	-23.4528
NC_000073.7	1.28E+08	Sec23ip	1.97E-07	-24.4046
NC_000073.7	1.36E+08	null	2.2E-08	25.18779
NC_000073.7	1.41E+08	Sigirr	1.99E-07	-20.9716
NC_000073.7	1.41E+08	Sigirr	4.72E-07	-25.4869
NC_000073.7	1.41E+08	Sigirr	1.38E-08	-21.4337
NC_000073.7	1.43E+08	Kcnq1	2.72E-07	-25.3075
NC_000074.7	4287815	Lrrc8e	2.47E-07	-15.0681
NC_000074.7	13541765	Tmem255b	2.97E-11	32.46395
NC_000074.7	70956086	Crlf1	3.57E-10	-13.7281
NC_000074.7	72914203	Rab8a	5.39E-09	-14.074
NC_000074.7	85511150	Nfix	1.41E-08	-32.1424
NC_000074.7	92526102	Irx3os	2.97E-10	-22.7178
NC_000074.7	1.05E+08	null	4.06E-07	31.75486
NC_000074.7	1.05E+08	null	4.87E-07	-16.2011
NC_000074.7	1.07E+08	1110028F18Rik	2.01E-08	16.98707
NC_000074.7	1.09E+08	Zfhx3	5.87E-07	-17.1494
NC_000074.7	1.16E+08	Wwox	5.77E-11	-25.7317
NC_000074.7	1.16E+08	Wwox	1.04E-09	-24.9919
NC_000074.7	1.2E+08	Gm32006	5.12E-07	41.67677
NC_000074.7	1.21E+08	Irf8	8.93E-12	-27.4282
NC_000074.7	1.21E+08	Irf8	1.73E-07	-13.9471
NC_000074.7	1.22E+08	null	1.73E-07	-13.9411
NC_000075.7	48449848	Nnmt	2.93E-09	10.94636
NC_000075.7	48557409	Gm53521	4.6E-07	14.67214
NC_000075.7	62251087	Anp32a	3.36E-08	-24.1428
NC_000075.7	65145673	Parp16	4.33E-07	-15.6683
NC_000075.7	74832681	null	7E-10	13.52686
NC_000075.7	77831912	Elovl5	9.96E-07	15.60411
NC_000075.7	82980974	Gm2065	2.59E-07	-20.9237
NC_000075.7	82980998	Gm2065	4.68E-11	-25.7519
NC_000075.7	85206749	Tent5a	5.72E-08	-36.599
NC_000075.7	85206806	Tent5a	2.25E-15	-52.4849
NC_000075.7	1E+08	null	1.39E-09	-22.8891
NC_000075.7	1.08E+08	Slc38a3	2.79E-09	16.95709
NC_000075.7	1.11E+08	Prss50	4.54E-07	19.23821
NC_000075.7	1.19E+08	Acvr2b	7.39E-07	18.13228
NC_000076.7	19814800	Map3k5	7.25E-09	-14.9709
NC_000076.7	60209324	Cdh23	7.89E-07	-22.3798
NC_000076.7	63419395	Ctnna3	2.48E-11	-18.8322
NC_000076.7	63419412	Ctnna3	3.15E-11	-19.3239
NC_000076.7	66773090	null	8.79E-08	-17.0131
NC_000076.7	76927003	Col18a1	2.01E-08	-32.4698
NC_000076.7	76927017	Col18a1	3.6E-08	-31.961
NC_000076.7	76927025	Col18a1	4.47E-07	-27.0906
NC_000076.7	79806098	Grin3b	2.38E-10	15.27984
NC_000076.7	79806165	Grin3b	1.68E-08	10.42615

NC_000076.7	79806230	Grin3b	6.34E-11	12.19684
NC_000076.7	80166151	Reep6	1.89E-10	-18.6519
NC_000076.7	80670344	Lingo3	3.11E-07	-12.1382
NC_000076.7	80671301	Lingo3	3.85E-08	-50.6618
NC_000076.7	81219635	Dohh	9.95E-08	-15.5724
NC_000076.7	81221132	Dohh	2.17E-07	-31.7199
NC_000076.7	95399303	Nudt4	8.82E-07	-12.4215
NC_000076.7	95399323	Nudt4	8.58E-07	-15.0719
NC_000076.7	95399374	Nudt4	3.95E-07	-13.5533
NC_000076.7	1.19E+08	null	3.24E-09	-39.0479
NC_000076.7	1.21E+08	null	3.87E-07	18.03103
NC_000076.7	1.21E+08	null	5.13E-09	20.64839
NC_000076.7	1.21E+08	Gm46204	2.79E-09	-11.3646
NC_000076.7	1.25E+08	null	4.49E-07	15.59824
NC_000076.7	1.27E+08	Cyp27b1	4.18E-09	-12.6991
NC_000076.7	1.27E+08	Agap2	5.72E-08	-24.6228
NC_000077.7	4051827	Sec14l2	2.16E-09	-28.203
NC_000077.7	5658196	Mrps24	3.36E-21	-16.4902
NC_000077.7	5658208	Mrps24	3.72E-20	-16.9837
NC_000077.7	5658348	Mrps24	3.85E-13	-19.0543
NC_000077.7	18964758	Meis1	4.17E-09	-25.1335
NC_000077.7	33706788	Kcnip1	5.06E-07	-17.6816
NC_000077.7	48762131	Irgm1	2.28E-10	-22.8186
NC_000077.7	60575347	null	4.94E-10	31.11004
NC_000077.7	60575441	null	1.76E-10	30.56335
NC_000077.7	63155828	null	7.89E-07	37.07767
NC_000077.7	63155944	null	2.28E-09	46.973
NC_000077.7	68952162	Borcs6	4.25E-10	-19.0157
NC_000077.7	70365978	null	1.47E-16	-16.1298
NC_000077.7	74721871	Mnt	1.68E-07	-10.3115
NC_000077.7	75322365	Serpinf2	2.41E-07	-19.3647
NC_000077.7	75322373	Serpinf2	5.6E-07	-18.5272
NC_000077.7	75606623	null	3.46E-09	-36.9832
NC_000077.7	75821226	Rph3al	1.56E-07	-16.4423
NC_000077.7	94040019	B230206L02Rik	1.4E-11	-10.4794
NC_000077.7	94101612	Tob1	1.43E-09	-10.2472
NC_000077.7	94101659	Tob1	1.47E-07	-11.2671
NC_000077.7	94101685	Tob1	1E-09	-11.5637
NC_000077.7	94101686	Tob1	4.52E-07	-10.5227
NC_000077.7	1E+08	Hap1	2.47E-14	10.76269
NC_000077.7	1.02E+08	Itga2b	5.42E-10	-36.3994
NC_000077.7	1.07E+08	null	7.46E-07	16.66708
NC_000077.7	1.09E+08	Gm36876	2.83E-08	-12.54
NC_000077.7	1.09E+08	Gm36876	2.5E-07	-11.1817
NC_000077.7	1.09E+08	Gm36876	5.3E-10	-11.0591
NC_000077.7	1.09E+08	Gm36876	2.57E-07	-10.7858
NC_000077.7	1.09E+08	Gm36876	3.74E-08	-15.3497

NC_000077.7	1.1E+08	Map2k6	4.25E-07	-13.8423
NC_000077.7	1.13E+08	Slc39a11	1.15E-10	-32.6895
NC_000077.7	1.16E+08	Ten1	6.8E-07	-11.3436
NC_000077.7	1.17E+08	Sec14l1	7.78E-08	26.16918
NC_000077.7	1.17E+08	Septin9	1.98E-07	36.40179
NC_000077.7	1.18E+08	null	2.61E-07	-11.1259
NC_000077.7	1.18E+08	null	5.21E-07	-14.0578
NC_000077.7	1.18E+08	null	3.04E-07	-10.953
NC_000077.7	1.18E+08	Dnah17	2.99E-09	-27.9099
NC_000077.7	1.19E+08	null	7.4E-07	-36.9444
NC_000078.7	55537309	Nfkbia	3.57E-09	-30.5063
NC_000078.7	72332666	Rtn1	5.34E-16	22.60683
NC_000078.7	72332690	Rtn1	1.66E-09	15.98316
NC_000078.7	73604191	null	9.92E-07	-17.7431
NC_000078.7	81008060	Slc10a1	2.73E-07	-13.3239
NC_000078.7	84464381	Bbof1	6.17E-09	-14.2495
NC_000078.7	86687756	null	3.46E-09	-26.9806
NC_000078.7	86687787	null	6.83E-08	-20.4216
NC_000078.7	91017422	Cep128	3.84E-07	19.71934
NC_000078.7	91017437	Cep128	1.42E-08	21.65385
NC_000078.7	91017460	Cep128	1.05E-10	30.53874
NC_000078.7	1.11E+08	Gm35558	5.68E-11	15.21708
NC_000078.7	1.11E+08	Tnfaip2	4.64E-07	-17.1367
NC_000078.7	1.13E+08	Tedc1	1.99E-08	-22.9396
NC_000079.7	35176976	Eci2	3.14E-16	-23.2358
NC_000079.7	35176987	Eci2	2.18E-14	-22.3761
NC_000079.7	44569050	null	2.59E-14	-14.2684
NC_000079.7	44804819	null	1.28E-09	-17.957
NC_000079.7	47375922	Rnf144b	4.61E-09	12.87497
NC_000079.7	48870636	Gm36784	6.1E-08	-37.1755
NC_000079.7	52723571	Gm2848	3.04E-09	20.45822
NC_000079.7	52723749	Gm2848	6.65E-10	18.69733
NC_000079.7	55672200	Dok3	3.8E-10	-20.6504
NC_000079.7	64048955	Gm46423	2.35E-09	-41.4423
NC_000079.7	64300949	Zfp367	7.91E-07	-17.9886
NC_000079.7	68046493	null	9.88E-07	-23.2581
NC_000079.7	68046525	null	2.59E-07	-20.6001
NC_000079.7	73081596	Gm30049	3.09E-07	28.1251
NC_000079.7	91142014	Atg10	5.52E-08	-15.8946
NC_000079.7	91142102	Atg10	2.65E-07	-13.517
NC_000079.7	93762463	Bhmt	4.89E-08	15.59576
NC_000079.7	94272806	Lhfpl2	4.51E-08	-28.2838
NC_000079.7	96805708	Hmgcr	5.5E-07	12.85201
NC_000079.7	1.02E+08	Pik3r1	1.28E-11	15.53669
NC_000079.7	1.08E+08	Zswim6	9.97E-11	-16.2383
NC_000079.7	1.08E+08	Zswim6	5.47E-12	-12.1758
NC_000079.7	1.08E+08	Zswim6	4.56E-11	-14.1541

NC_000079.7	1.12E+08	null	7.58E-07	-15.2364
NC_000079.7	1.12E+08	null	2.58E-09	-20.9259
NC_000080.7	4237414	Nr1d2	1.07E-07	-19.3037
NC_000080.7	10661547	Ptprg	2.24E-09	-20.4555
NC_000080.7	25499150	Zmiz1	1.16E-08	-13.5864
NC_000080.7	28231270	Wnt5a	2.82E-07	10.01132
NC_000080.7	30609646	Itih4	1.13E-07	-22.4318
NC_000080.7	42979577	Cdv3-ps	1.41E-12	-11.5081
NC_000080.7	42979629	Cdv3-ps	3.16E-20	-16.3152
NC_000080.7	45680860	Gm34250	1.36E-09	-18.7835
NC_000080.7	45682764	Gm34250	5.56E-07	20.32901
NC_000080.7	45682955	Gm34250	5.83E-07	11.6025
NC_000080.7	47122064	Samd4	1.77E-07	-16.0057
NC_000080.7	49538122	Slc35f4	2.17E-08	-18.2492
NC_000080.7	51193852	Gm38316	2.51E-11	-40.8303
NC_000080.7	57636444	Cryl1	9.1E-08	-13.2997
NC_000080.7	60499601	Mtmr6	4.41E-11	18.23078
NC_000080.7	66315401	Adam2	2.09E-08	-18.3324
NC_000080.7	76245275	Gtf2f2	4.3E-07	16.52277
NC_000080.7	1.03E+08	Gm41236	3.83E-10	-21.419
NC_000080.7	1.21E+08	null	5.88E-07	-11.2698
NC_000081.7	5137633	Card6	1.1E-08	-10.377
NC_000081.7	5137636	Card6	6.72E-12	-13.3346
NC_000081.7	75765262	Naprt	3.6E-08	-25.7396
NC_000081.7	75765281	Naprt	6.34E-08	-24.6872
NC_000081.7	75869889	Mapk15	1.43E-09	-66.657
NC_000081.7	76464269	Adck5	5.25E-10	-17.8193
NC_000081.7	78851927	Triobp	3.49E-08	-11.8301
NC_000081.7	81853370	null	1.16E-07	33.23558
NC_000081.7	82005655	Mei1	2.87E-12	15.25111
NC_000081.7	83478531	Ttll12	7.95E-07	-26.5038
NC_000081.7	85499949	null	1.81E-09	-25.5943
NC_000081.7	94415977	null	1.44E-09	13.38718
NC_000081.7	96363155	Gm41408	2.74E-09	-16.8565
NC_000081.7	99192523	Fam186b	1.3E-08	-11.0421
NC_000081.7	1.01E+08	Cela1	1.73E-07	-31.9416
NC_000081.7	1.01E+08	Scn8a	3.35E-08	18.10537
NC_000081.7	1.02E+08	Krt79	5.33E-07	-29.3271
NC_000081.7	1.02E+08	Csad	8.62E-24	-12.165
NC_000081.7	1.02E+08	Csad	0	-15.7316
NC_000082.7	3155382	null	1.38E-10	12.95519
NC_000082.7	4745670	Mgrn1	4.23E-09	-50.639
NC_000082.7	29561619	null	5.09E-07	-27.5479
NC_000082.7	29561626	null	2.59E-07	-26.1764
NC_000082.7	29561633	null	1.2E-09	-35.249
NC_000082.7	43627460	Drd3	9.14E-12	-13.4797
NC_000082.7	45664552	Phldb2	2.7E-08	-10.8731

NC_000082.7	45664614	Phldb2	1.6E-13	-15.4114
NC_000082.7	49595873	Gm15518	9.87E-07	16.56415
NC_000082.7	57211989	Cmss1	1.83E-07	-21.4386
NC_000082.7	62693598	Pros1	4.91E-07	-25.6071
NC_000082.7	76477084	null	3.29E-07	17.13088
NC_000082.7	78131728	Cxadr	8.47E-07	14.38515
NC_000082.7	78131734	Cxadr	7.88E-07	14.66611
NC_000082.7	85562860	null	1.59E-09	-20.1618
NC_000082.7	93386510	Setd4	8.44E-10	-21.5405
NC_000083.7	10554893	null	2.91E-15	-32.9768
NC_000083.7	10554918	null	1.29E-07	-20.7516
NC_000083.7	14296247	null	1.18E-07	-14.5748
NC_000083.7	22893044	Gm9805	7.81E-08	-16.3942
NC_000083.7	25069933	Hagh	4.34E-07	-11.8546
NC_000083.7	27259346	null	3.7E-08	-36.0051
NC_000083.7	29117400	Bnip5	9.3E-08	21.68438
NC_000083.7	32076009	Sik1	3.77E-08	-14.8049
NC_000083.7	32631040	Gm41569	2.82E-07	14.88321
NC_000083.7	34170439	B3galt4	1.99E-07	-41
NC_000083.7	34170455	B3galt4	5.96E-08	-42.4386
NC_000083.7	34812228	Pbx2	2.59E-09	-13.8469
NC_000083.7	35107336	null	2.1E-11	-22.6062
NC_000083.7	35439767	Nfkbil1	3.29E-07	26.0609
NC_000083.7	35439773	Nfkbil1	1.94E-08	28.08691
NC_000083.7	35439782	Nfkbil1	3.9E-08	28.03543
NC_000083.7	35439795	Nfkbil1	7.65E-08	27.86685
NC_000083.7	35439803	Nfkbil1	5.92E-09	29.8462
NC_000083.7	35501221	null	1.92E-07	-10.7896
NC_000083.7	40154203	Gm26917	6.95E-20	-15.5985
NC_000083.7	40154376	Gm26917	2.07E-26	-10.971
NC_000083.7	40154395	Gm26917	1.42E-36	-13.671
NC_000083.7	40154409	Gm26917	1.57E-38	-16.4592
NC_000083.7	40154463	Gm26917	2.53E-40	-11.4493
NC_000083.7	40154483	Gm26917	8.55E-34	-10.5873
NC_000083.7	40154537	Gm26917	2.67E-32	-10.9157
NC_000083.7	40154818	Gm26917	0	-13.1274
NC_000083.7	40154829	Gm26917	1.8E-41	-10.2812
NC_000083.7	40154862	Gm26917	5.78E-32	-11.7658
NC_000083.7	40154927	Gm26917	0	-11.1956
NC_000083.7	40155012	Gm26917	2.88E-23	-11.4663
NC_000083.7	40155013	Gm26917	0	-10.9185
NC_000083.7	40155059	Gm26917	2.85E-41	-10.8386
NC_000083.7	40155063	Gm26917	6.13E-37	-10.7016
NC_000083.7	40155065	Gm26917	2.43E-34	-10.6445
NC_000083.7	40155098	Gm26917	2.52E-36	-11.4413
NC_000083.7	40155103	Gm26917	1.21E-36	-11.6135
NC_000083.7	40155131	Gm26917	1.48E-11	-10.6049

NC_000083.7	40155132	Gm26917	1.12E-32	-10.0523
NC_000083.7	40155280	Gm26917	5.65E-22	-11.062
NC_000083.7	40155298	Gm26917	9.25E-21	-10.9182
NC_000083.7	40155300	Gm26917	1.14E-24	-11.537
NC_000083.7	40155306	Gm26917	1.46E-25	-11.3372
NC_000083.7	40155314	Gm26917	1.87E-21	-10.2993
NC_000083.7	40155321	Gm26917	2.35E-27	-13.2498
NC_000083.7	40155351	Gm26917	6.07E-25	-10.9438
NC_000083.7	40155376	Gm26917	7.82E-30	-10.2541
NC_000083.7	40155401	Gm26917	3.85E-29	-10.3192
NC_000083.7	40155422	Gm26917	2.99E-31	-13.0032
NC_000083.7	40155433	Gm26917	1.85E-30	-11.2838
NC_000083.7	40155443	Gm26917	1.65E-37	-11.0959
NC_000083.7	40155455	Gm26917	4.13E-34	-10.9248
NC_000083.7	40155488	Gm26917	8.28E-37	-11.4665
NC_000083.7	40155535	Gm26917	2.55E-26	-10.2774
NC_000083.7	40155568	Gm26917	1.37E-07	-21.0882
NC_000083.7	40155569	Gm26917	8.66E-08	-22.348
NC_000083.7	40155579	Gm26917	1.59E-09	-22.4818
NC_000083.7	40155582	Gm26917	4.01E-08	-16.8617
NC_000083.7	40155976	Gm26917	0	-11.3313
NC_000083.7	40155978	Gm26917	0	-12.8318
NC_000083.7	40155982	Gm26917	0	-10.7398
NC_000083.7	40155987	Gm26917	0	-12.2777
NC_000083.7	40155997	Gm26917	0	-10.5671
NC_000083.7	40155999	Gm26917	0	-10.4865
NC_000083.7	40156017	Gm26917	0	-10.2157
NC_000083.7	40156021	Gm26917	0	-10.3104
NC_000083.7	40156040	Gm26917	0	-11.4683
NC_000083.7	40156042	Gm26917	0	-11.2161
NC_000083.7	40156081	Gm26917	0	-11.9652
NC_000083.7	40156084	Gm26917	0	-12.6014
NC_000083.7	40156104	Gm26917	0	-10.5187
NC_000083.7	40156124	Gm26917	0	-12.1311
NC_000083.7	40156128	Gm26917	0	-13.0283
NC_000083.7	40156245	Gm26917	8.58E-20	-13.5435
NC_000083.7	40156287	Gm26917	4.39E-24	-11.1917
NC_000083.7	40156322	Gm26917	1.43E-19	-11.2164
NC_000083.7	40156324	Gm26917	1.81E-26	-10.973
NC_000083.7	40156336	Gm26917	1.04E-30	-12.4854
NC_000083.7	40156343	Gm26917	6.98E-24	-10.7148
NC_000083.7	40156383	Gm26917	2.19E-23	-10.2824
NC_000083.7	40156407	Gm26917	4.04E-27	-10.2993
NC_000083.7	40156420	Gm26917	1.56E-19	-12.3467
NC_000083.7	40156635	Gm26917	1.8E-07	-11.9258
NC_000083.7	40156643	Gm26917	2.65E-10	-11.8771
NC_000083.7	40156674	Gm26917	5.26E-07	-10.7248

NC_000083.7	40156676	Gm26917	1.76E-08	-12.5496
NC_000083.7	40156685	Gm26917	1.45E-07	-10.976
NC_000083.7	40156691	Gm26917	1.79E-09	-13.2797
NC_000083.7	40156698	Gm26917	5.67E-11	-15.349
NC_000083.7	40156709	Gm26917	8.29E-09	-12.1723
NC_000083.7	40157140	Gm26917	3.16E-40	-14.2265
NC_000083.7	40157165	Gm26917	5.71E-31	-11.1346
NC_000083.7	40157171	Gm26917	5.78E-39	-11.8851
NC_000083.7	40157188	Gm26917	7.59E-37	-10.2605
NC_000083.7	40157221	Gm26917	9.81E-45	-10.712
NC_000083.7	40157234	Gm26917	0	-11.2189
NC_000083.7	40157308	Gm26917	8.47E-33	-13.5473
NC_000083.7	40157312	Gm26917	2.44E-40	-13.8176
NC_000083.7	40159155	Gm26917	1.57E-34	-10.1734
NC_000083.7	40159172	Gm26917	2.16E-22	-15.3613
NC_000083.7	40159179	Gm26917	8.11E-19	-10.944
NC_000083.7	40159182	Gm26917	5.34E-17	-11.5986
NC_000083.7	40159186	Gm26917	8.99E-22	-10.8541
NC_000083.7	40159188	Gm26917	8.88E-25	-13.1381
NC_000083.7	40159197	Gm26917	1.24E-21	-12.6409
NC_000083.7	40159238	Gm26917	5.09E-18	-11.7954
NC_000083.7	40159249	Gm26917	5.49E-20	-12.4348
NC_000083.7	40159259	Gm26917	1.18E-10	-12.1257
NC_000083.7	40159265	Gm26917	2.89E-18	-18.6466
NC_000083.7	45930440	Gm52283	5.84E-07	-11.3179
NC_000083.7	45930487	Gm52283	6.83E-18	-22.0837
NC_000083.7	45930508	Gm52283	4.19E-08	-13.9576
NC_000083.7	56306956	Stap2	4.15E-08	13.24999
NC_000083.7	79538450	null	2.29E-07	-24.3051
NC_000083.7	80581446	Dhx57	1.81E-08	-19.427
NC_000083.7	87110430	Epas1	4.43E-10	22.29704
NC_000083.7	88468062	Gm31499	1.97E-08	-22.2573
NC_000083.7	88468071	Gm31499	3.11E-11	-29.1926
NC_000083.7	91395537	Nrxn1	8.14E-08	24.29077
NC_000084.7	5116403	Svil	1.22E-08	19.74669
NC_000084.7	23940683	Mapre2	2.54E-09	-31.6043
NC_000084.7	23940732	Mapre2	1.51E-10	-36.0251
NC_000084.7	36690622	Slc4a9	4.08E-10	-12.6308
NC_000084.7	36690683	Slc4a9	2.08E-15	-13.9442
NC_000084.7	36690692	Slc4a9	5.63E-19	-15.4416
NC_000084.7	37870449	Gm37013	4.03E-10	23.14609
NC_000084.7	37880839	Gm37013	2.51E-08	-45.0835
NC_000084.7	37880883	Gm37013	3.11E-07	-40.0005
NC_000084.7	39496200	Arhgap26	3.48E-10	20.27831
NC_000084.7	39496201	Arhgap26	8.62E-10	19.56775
NC_000084.7	39496219	Arhgap26	3.96E-09	17.71425
NC_000084.7	39496224	Arhgap26	6.16E-11	19.5889

NC_000084.7	39496242	Arhgap26	1.57E-14	23.73366
NC_000084.7	39496254	Arhgap26	1.33E-07	15.63328
NC_000084.7	39496255	Arhgap26	4.28E-13	22.12536
NC_000084.7	50179584	Tnfaip8	1.16E-07	-24.5058
NC_000084.7	50179611	Tnfaip8	1.14E-08	-25.5633
NC_000084.7	52886949	null	1.36E-07	-12.0291
NC_000084.7	69704307	Tcf4	6.68E-13	-30.1895
NC_000084.7	69704339	Tcf4	3.78E-07	-23.4545
NC_000084.7	69704365	Tcf4	1.14E-09	-25.8689
NC_000084.7	69747218	Tcf4	2.12E-07	15.87024
NC_000084.7	75581792	Ctif	1.6E-09	28.67162
NC_000084.7	76520044	null	6.39E-07	-10.5828
NC_000084.7	80212813	null	5.19E-11	14.42053
NC_000084.7	80212839	null	1.59E-08	13.66231
NC_000084.7	80213078	null	9.14E-09	10.60608
NC_000084.7	81141388	null	6.93E-09	-37.6017
NC_000084.7	81242412	null	8.67E-13	16.13765
NC_000084.7	81242421	null	1.78E-17	18.48086
NC_000084.7	81242540	null	8.08E-09	14.14596
NC_000085.7	3693979	Lrp5	9.67E-11	-23.8346
NC_000085.7	5699674	Rela	5.91E-08	10.54698
NC_000085.7	23016087	C330002G04Rik	1.97E-08	13.67141
NC_000085.7	31845977	A1cf	3.49E-08	-22.7997
NC_000085.7	37484360	Gm32294	1.36E-08	13.88145
NC_000085.7	44386328	Scd1	1.13E-10	-10.2363
NC_000085.7	44386378	Scd1	4.62E-10	-19.3285
NC_000085.7	44386379	Scd1	2.51E-26	-24.7947
NC_000085.7	44386432	Scd1	1.97E-12	-12.6068
NC_000085.7	44801133	Pax2	3.56E-07	-10.3301
NC_000085.7	46607433	Wbp1l	1.41E-07	24.446
NC_000085.7	53393215	null	2.95E-08	15.31934
NC_000085.7	55397356	Vti1a	7.24E-08	-21.4189
NC_000085.7	58358489	Gfra1	1.63E-12	13.14619
NC_000086.8	72286612	Xlr4c	3.45E-10	23.36278
NC_000086.8	1.01E+08	Gcna	2.97E-08	-11.1473
NC_000086.8	1.26E+08	null	4.52E-07	21.82104
NC_000086.8	1.69E+08	Mid1	1.84E-15	-13.1468
NC_005089.1	3401	ND1	0	-12.0897
NC_005089.1	3410	ND1	0	-13.0808
NC_005089.1	3412	ND1	0	-12.9877
NW_023337853.1	467	null	0	-11.0075
NW_023337853.1	481	null	0	-10.4472
NW_023337853.1	969	null	3.07E-07	-13.6488
NW_023337853.1	1003	null	7.89E-07	-12.0854
NW_023337853.1	1037	null	1.25E-20	-11.5005
NW_023337853.1	1082	null	1.51E-28	-11.3327
NW_023337853.1	1092	null	1.68E-15	-10.0647

NW_023337853.1	1277	null	1.8E-09	-12.0728
NW_023337853.1	1280	null	1.34E-07	-11.2901
NW_023337853.1	1354	null	8.15E-10	-12.3193
NW_023337853.1	1366	null	4.2E-09	-11.8437
NW_023337853.1	1390	null	6.54E-07	-10.8186
NW_023337853.1	1402	null	2.35E-13	-11.1662
NW_023337853.1	1440	null	6.38E-18	-10.4931
NW_023337853.1	1452	null	1.01E-13	-10.4324
NW_023337853.1	30820	null	0	-10.2079
NW_023337853.1	30823	null	0	-10.5584
NW_023337853.1	30825	null	0	-11.8559
NW_023337853.1	30831	null	0	-12.5483
NW_023337853.1	30858	null	0	-11.6635
NW_023337853.1	30870	null	0	-11.0041
NW_023337853.1	30897	null	0	-10.9927
NW_023337853.1	30903	null	0	-10.9944
NW_023337853.1	30957	null	0	-10.2099
**CTR vs HFD+beer**				
**Chromosome**	**Start**	**Gene**	**FDR**	**Meth Difference**
NC_000067.7	18148763	null	9.15E-07	-37.9819
NC_000067.7	34278984	Dst	2.8E-09	20.21622
NC_000067.7	34278993	Dst	1.33E-14	26.67485
NC_000067.7	34278996	Dst	8.31E-11	23.18049
NC_000067.7	34279012	Dst	2.97E-10	21.98474
NC_000067.7	86241679	null	3.05E-07	-37.2976
NC_000067.7	1.37E+08	Gm41958	1.36E-08	33.41478
NC_000067.7	1.55E+08	null	2.38E-07	28.97396
NC_000067.7	1.72E+08	Kcnj10	3.01E-11	-15.4204
NC_000067.7	1.78E+08	Catspere2	1.48E-13	-26.0686
NC_000067.7	1.89E+08	Ush2a	1.57E-08	12.28874
NC_000067.7	1.89E+08	Ush2a	8.2E-08	11.31436
NC_000067.7	1.9E+08	null	7.91E-07	-17.3266
NC_000068.8	3888925	Gm36031	1.84E-07	15.64917
NC_000068.8	3889017	Gm36031	5.6E-07	14.77433
NC_000068.8	32546130	Eng	7.53E-10	31.40619
NC_000068.8	32546141	Eng	3.34E-11	35.05189
NC_000068.8	32546167	Eng	1.16E-10	33.29726
NC_000068.8	32546219	Eng	1.46E-10	35.17522
NC_000068.8	32546263	Eng	7.18E-10	32.92596
NC_000068.8	32546279	Eng	4.31E-07	27.53738
NC_000068.8	34924782	Hc	4.57E-17	32.13766
NC_000068.8	34947409	Hc	1.07E-07	24.98314
NC_000068.8	34947413	Hc	8.28E-22	36.22471
NC_000068.8	34947414	Hc	1.63E-10	33.2124
NC_000068.8	91465857	F2	2.26E-11	11.01223
NC_000068.8	1.46E+08	Rin2	1.12E-09	-19.1884
NC_000068.8	1.48E+08	9030622O22Rik	2.78E-08	13.23826

NC_000068.8	1.52E+08	Rbck1	1.69E-07	17.75016
NC_000068.8	1.63E+08	null	1.78E-09	-16.3408
NC_000068.8	1.63E+08	null	7.66E-07	-12.5918
NC_000068.8	1.65E+08	Slc12a5	7.02E-09	-27.1916
NC_000068.8	1.65E+08	null	4.88E-07	-27.8211
NC_000068.8	1.68E+08	null	4.84E-08	29.13266
NC_000068.8	1.68E+08	null	5.72E-07	-19.5433
NC_000068.8	1.69E+08	null	4.21E-08	41.31985
NC_000068.8	1.75E+08	Gm14616	3.27E-09	26.67227
NC_000069.7	32759730	null	9.35E-07	24.4187
NC_000069.7	52508228	null	7.1E-08	41.12226
NC_000069.7	79286719	Gm17359	5.65E-14	22.17875
NC_000069.7	79286756	Gm17359	1.43E-07	16.12089
NC_000069.7	79286807	Gm17359	3.47E-07	16.72447
NC_000069.7	79286863	Gm17359	2.05E-11	19.06856
NC_000069.7	79286872	Gm17359	3.62E-12	19.8484
NC_000069.7	79634114	Rxfp1	2.24E-09	22.38039
NC_000069.7	94589673	null	2.27E-09	20.85647
NC_000069.7	94590831	null	3.44E-13	23.74116
NC_000069.7	94593386	null	7.48E-14	31.5709
NC_000069.7	94593388	null	1.37E-08	24.98029
NC_000069.7	94593389	null	8.3E-08	19.48467
NC_000069.7	1.22E+08	Bcar3	1.37E-07	26.95539
NC_000069.7	1.3E+08	Lrit3	1.11E-11	15.86864
NC_000069.7	1.3E+08	Lrit3	5.81E-08	21.69197
NC_000069.7	1.48E+08	Gm40175	5.53E-07	-25.9449
NC_000070.7	27768037	null	2.67E-07	-12.1776
NC_000070.7	44963316	null	1.91E-09	13.97373
NC_000070.7	55750033	null	2.04E-07	21.61624
NC_000070.7	55750106	null	3.26E-07	21.39736
NC_000070.7	55750108	null	7.52E-08	22.33616
NC_000070.7	99347825	null	2.97E-11	25.1665
NC_000070.7	99984008	Ror1	2.76E-07	14.04597
NC_000070.7	1.05E+08	Plpp3	3.09E-09	21.9115
NC_000070.7	1.12E+08	Skint3	3.11E-07	15.0936
NC_000070.7	1.18E+08	St3gal3	4.98E-07	15.0724
NC_000070.7	1.18E+08	Ptprf	7.67E-08	13.80637
NC_000070.7	1.28E+08	null	1E-09	36.76358
NC_000070.7	1.34E+08	Cep85	7.01E-11	14.8462
NC_000070.7	1.36E+08	E2f2	1.55E-07	25.4878
NC_000070.7	1.36E+08	E2f2	4.48E-07	26.57229
NC_000070.7	1.47E+08	Zfp991	8.43E-07	-20.0183
NC_000070.7	1.47E+08	Zfp991	9.62E-09	-23.3887
NC_000070.7	1.48E+08	Draxin	1.64E-07	-16.5402
NC_000070.7	1.49E+08	Gm572	4.34E-08	-49.5722
NC_000070.7	1.51E+08	Gm42356	4.32E-07	13.757
NC_000070.7	1.53E+08	Nphp4	1.51E-08	-21.1885

NC_000071.7	8132856	Adam22	3.83E-08	24.31256
NC_000071.7	15061715	null	1.83E-07	38.03189
NC_000071.7	31788910	Gm38424	2.02E-07	19.46543
NC_000071.7	35819150	Htra3	1.23E-12	-27.8104
NC_000071.7	38457473	Otop1	5.53E-07	-17.8498
NC_000071.7	66717900	Apbb2	2.02E-07	-19.7801
NC_000071.7	90652739	Afp	1.73E-09	21.03762
NC_000071.7	92512900	Art3	5.69E-08	28.02773
NC_000071.7	92512910	Art3	4.77E-11	31.28722
NC_000071.7	92512919	Art3	5.5E-09	28.33236
NC_000071.7	1.04E+08	Aff1	5.16E-12	47.54048
NC_000071.7	1.04E+08	Aff1	2.42E-12	47.31997
NC_000071.7	1.05E+08	Spp1	1.25E-21	32.09166
NC_000071.7	1.05E+08	Pkd2	5.58E-19	19.82094
NC_000071.7	1.05E+08	Pkd2	5.65E-14	17.03311
NC_000071.7	1.05E+08	Pkd2	1.4E-13	18.56555
NC_000071.7	1.12E+08	null	3.99E-09	23.26893
NC_000071.7	1.14E+08	Svop	5.52E-07	11.12657
NC_000071.7	1.15E+08	null	1.94E-08	39.21636
NC_000071.7	1.16E+08	Pla2g1b	3.84E-08	13.20701
NC_000071.7	1.16E+08	Pxn	3.9E-07	35.15314
NC_000071.7	1.16E+08	Pxn	4.92E-07	19.43187
NC_000071.7	1.22E+08	Sh2b3	3.67E-07	25.2413
NC_000071.7	1.23E+08	Camkk2	2.51E-11	15.8903
NC_000071.7	1.23E+08	Camkk2	2.37E-13	17.70415
NC_000071.7	1.23E+08	Camkk2	4.93E-08	13.66036
NC_000071.7	1.23E+08	Camkk2	4.75E-10	15.18408
NC_000071.7	1.23E+08	Camkk2	1.24E-11	16.04866
NC_000071.7	1.23E+08	Camkk2	1.48E-09	14.63199
NC_000071.7	1.23E+08	Anapc5	6.31E-09	-21.2831
NC_000071.7	1.24E+08	Vps37b	3.91E-08	25.09696
NC_000071.7	1.24E+08	Arl6ip4	1.31E-10	10.77224
NC_000071.7	1.24E+08	Arl6ip4	1.92E-12	11.55253
NC_000071.7	1.24E+08	Arl6ip4	2.84E-20	14.88375
NC_000071.7	1.24E+08	Arl6ip4	9.48E-24	17.51041
NC_000071.7	1.24E+08	Arl6ip4	5.89E-12	11.15985
NC_000071.7	1.25E+08	Ncor2	1.2E-12	19.93016
NC_000071.7	1.35E+08	Cldn3	2.4E-14	13.02121
NC_000071.7	1.38E+08	Mospd3	8.74E-07	22.40279
NC_000071.7	1.41E+08	Gna12	1.14E-09	14.54557
NC_000071.7	1.46E+08	Cdk8	1.27E-08	14.03087
NC_000071.7	1.46E+08	Cdk8	4.2E-13	16.08347
NC_000072.7	28928157	Snd1	7.06E-07	-12.7131
NC_000072.7	28928172	Snd1	4.27E-11	-15.7421
NC_000072.7	28928187	Snd1	8.48E-10	-15.8137
NC_000072.7	28928202	Snd1	2.28E-09	-14.192
NC_000072.7	28928213	Snd1	8.86E-11	-16.0127

NC_000072.7	28928220	Snd1	1.04E-07	-12.2713
NC_000072.7	28928227	Snd1	2.22E-07	-12.2505
NC_000072.7	28928236	Snd1	2.64E-09	-13.3222
NC_000072.7	38234484	null	1.4E-15	24.99035
NC_000072.7	43620669	Tpk1	1.31E-07	-18.4198
NC_000072.7	86470507	null	7.44E-07	19.13238
NC_000072.7	88753491	Mgll	9.47E-11	18.80437
NC_000072.7	97256128	null	2.82E-13	25.51711
NC_000072.7	1.15E+08	null	2.3E-07	-13.1422
NC_000072.7	1.19E+08	Cacna1c	1.09E-09	-25.6597
NC_000072.7	1.21E+08	Mical3	3.68E-08	18.92742
NC_000072.7	1.21E+08	Usp18	7.3E-11	25.08721
NC_000072.7	1.24E+08	null	7.82E-12	-25.9419
NC_000072.7	1.25E+08	Cd4	2.52E-15	17.00358
NC_000072.7	1.27E+08	null	6.44E-07	12.4573
NC_000072.7	1.36E+08	Gm36897	1.77E-08	24.40036
NC_000072.7	1.37E+08	Erp27	6.81E-07	11.29725
NC_000072.7	1.37E+08	Erp27	2.4E-11	15.81876
NC_000072.7	1.38E+08	Dera	2.69E-16	13.2521
NC_000072.7	1.43E+08	null	5.97E-15	28.76107
NC_000072.7	1.43E+08	null	2.03E-17	31.92174
NC_000072.7	1.43E+08	null	3.43E-07	18.5917
NC_000073.7	6762882	Usp29	1.23E-07	-28.308
NC_000073.7	19072743	D830036C21Rik	3.41E-11	12.83414
NC_000073.7	36227984	null	9.85E-08	-20.3181
NC_000073.7	44601043	Tsks	1.45E-09	-11.3735
NC_000073.7	44601120	Tsks	4.69E-16	-13.8263
NC_000073.7	45262530	Fgf21	7.61E-34	17.01593
NC_000073.7	45578545	Odad1	3.01E-07	21.05639
NC_000073.7	45578641	Odad1	3.38E-09	21.73812
NC_000073.7	46486859	null	8.15E-08	23.09315
NC_000073.7	46491476	Ldha	4.84E-10	12.6729
NC_000073.7	48813661	Nav2	2.41E-08	-38.7965
NC_000073.7	55201180	null	1.13E-07	18.79779
NC_000073.7	80106275	Blm	3.17E-08	23.50718
NC_000073.7	81543601	Hdgfl3	1.96E-09	15.56749
NC_000073.7	98875934	Mogat2	1.67E-08	-21.534
NC_000073.7	1.05E+08	null	2.28E-08	-14.4768
NC_000073.7	1.12E+08	Parva	4.07E-09	17.22005
NC_000073.7	1.13E+08	null	8.85E-07	34.46437
NC_000073.7	1.13E+08	null	7.17E-12	46.75874
NC_000073.7	1.32E+08	null	1.23E-09	-14.9703
NC_000073.7	1.42E+08	Dusp8	4.7E-08	25.05317
NC_000073.7	1.42E+08	Dusp8	3.87E-07	22.16784
NC_000073.7	1.42E+08	Dusp8	2.19E-08	24.40705
NC_000074.7	11054683	Irs2	4.06E-08	-37.1689
NC_000074.7	35883829	Gm34911	7.65E-10	19.09153

NC_000074.7	35883947	Gm34911	2.15E-12	52.52893
NC_000074.7	35883984	Gm34911	1.32E-08	36.24089
NC_000074.7	35884047	Gm34911	1.17E-07	35.04795
NC_000074.7	49247085	Tenm3	3.32E-10	12.01161
NC_000074.7	92439583	Gm36325	7.14E-07	-23.5789
NC_000074.7	1.1E+08	Zfhx3	1.93E-10	21.14441
NC_000074.7	1.21E+08	Gse1	1.52E-14	35.07081
NC_000074.7	1.21E+08	Gse1	3.5E-07	-22.2718
NC_000074.7	1.22E+08	null	5.59E-08	-13.9146
NC_000074.7	1.22E+08	Fbxo31	5.47E-07	15.09949
NC_000075.7	4259664	null	1.13E-08	21.18235
NC_000075.7	20784177	Shfl	2.81E-09	-21.8297
NC_000075.7	59509188	Celf6	1.35E-16	18.55347
NC_000075.7	63605746	Smad3	2.36E-08	15.32473
NC_000075.7	70119311	Gm26849	1.56E-07	-10.7664
NC_000075.7	74832680	null	6.61E-07	23.62618
NC_000075.7	74832681	null	3.39E-31	20.39478
NC_000075.7	77425781	Lrrc1	3.2E-08	45.84773
NC_000075.7	82980969	Gm2065	1.48E-14	-25.1735
NC_000075.7	82980974	Gm2065	5.51E-12	-22.4096
NC_000075.7	82980998	Gm2065	4.33E-16	-26.5861
NC_000075.7	82981009	Gm2065	5.09E-11	-22.8314
NC_000075.7	1.15E+08	null	3.51E-08	23.15673
NC_000076.7	4820215	Esr1	8.09E-11	10.41373
NC_000076.7	63419412	Ctnna3	3.16E-08	-15.6877
NC_000076.7	77736731	Lrrc3	1.85E-18	29.87005
NC_000076.7	77736755	Lrrc3	9.83E-28	39.33009
NC_000076.7	77736759	Lrrc3	2.31E-13	26.21668
NC_000076.7	77736771	Lrrc3	2.74E-17	29.27546
NC_000076.7	79806098	Grin3b	1.24E-16	16.56607
NC_000076.7	79806230	Grin3b	4.13E-12	10.62791
NC_000076.7	79806249	Grin3b	3.12E-16	16.03767
NC_000076.7	80806096	Gng7	1.43E-07	-13.7331
NC_000076.7	93191592	null	4.76E-08	-26.9481
NC_000076.7	1.21E+08	null	3.38E-07	19.1503
NC_000076.7	1.21E+08	null	4.2E-08	20.44745
NC_000076.7	1.21E+08	null	1.04E-07	19.59878
NC_000076.7	1.21E+08	null	9.76E-13	24.78177
NC_000076.7	1.27E+08	Lrp1	4.14E-07	13.55403
NC_000076.7	1.28E+08	Timeless	2.45E-07	13.32948
NC_000077.7	5958867	Camk2b	1.89E-08	14.77182
NC_000077.7	5958876	Camk2b	4.59E-10	16.17479
NC_000077.7	5958883	Camk2b	1.7E-09	15.71219
NC_000077.7	5958950	Camk2b	2.59E-10	16.3238
NC_000077.7	5958958	Camk2b	2.6E-08	14.77948
NC_000077.7	5958975	Camk2b	3.55E-10	16.35036
NC_000077.7	5959050	Camk2b	2.3E-09	15.96283

NC_000077.7	35839580	Wwc1	3.34E-09	-41.4731
NC_000077.7	35839655	Wwc1	5.23E-09	-44.7993
NC_000077.7	57561645	Galnt10	5.6E-08	-23.4738
NC_000077.7	60120812	Tom1l2	2.4E-08	-28.0596
NC_000077.7	60120916	Tom1l2	2.25E-07	-30.5844
NC_000077.7	69008528	9130213A22Rik	4.86E-07	10.22466
NC_000077.7	70117572	Bcl6b	1.94E-13	26.30699
NC_000077.7	78316323	null	1.35E-14	27.51327
NC_000077.7	87841836	null	1.62E-17	16.713
NC_000077.7	98287154	Pgap3	1.11E-08	-29.1312
NC_000077.7	1.13E+08	null	7.31E-07	20.92869
NC_000077.7	1.13E+08	2610035D17Rik	1.29E-08	32.54548
NC_000077.7	1.13E+08	2610035D17Rik	1.95E-08	32.11588
NC_000077.7	1.13E+08	2610035D17Rik	1.9E-07	29.8598
NC_000077.7	1.13E+08	2610035D17Rik	7.11E-10	34.91708
NC_000077.7	1.13E+08	2610035D17Rik	3.7E-09	37.58692
NC_000077.7	1.13E+08	Slc39a11	3.16E-18	-36.1043
NC_000077.7	1.13E+08	Slc39a11	4.75E-17	-30.6618
NC_000077.7	1.13E+08	Slc39a11	3.17E-08	-22.9758
NC_000077.7	1.16E+08	Myo15b	2.08E-07	12.90513
NC_000077.7	1.16E+08	Myo15b	1.08E-08	17.30997
NC_000077.7	1.16E+08	Myo15b	1.19E-11	17.01361
NC_000077.7	1.16E+08	null	1.89E-07	19.65823
NC_000077.7	1.16E+08	null	1.81E-07	16.17861
NC_000077.7	1.18E+08	Dnah17	1.59E-07	-28.06
NC_000078.7	19754559	null	1.67E-07	19.94142
NC_000078.7	21828172	6030426L16Rik	4.69E-08	17.63264
NC_000078.7	29404809	null	1.63E-07	24.98778
NC_000078.7	72332666	Rtn1	2.72E-07	13.34568
NC_000078.7	72332690	Rtn1	7.09E-07	12.51108
NC_000078.7	75880396	Syne2	2.65E-17	13.15957
NC_000078.7	80049618	null	4.4E-10	38.16718
NC_000078.7	85337504	Zc2hc1c	9.48E-13	25.53879
NC_000078.7	91017422	Cep128	1.63E-07	22.24316
NC_000078.7	91017437	Cep128	3E-16	32.53315
NC_000078.7	91017460	Cep128	2.99E-15	33.32513
NC_000078.7	1.09E+08	null	8.24E-08	-21.3423
NC_000078.7	1.15E+08	Igh	7.68E-07	-13.7647
NC_000079.7	35176987	Eci2	3.66E-07	-15.0058
NC_000079.7	41016563	Gcnt2	1.8E-07	18.73669
NC_000079.7	41016575	Gcnt2	6.84E-15	25.77671
NC_000079.7	41016628	Gcnt2	5.23E-07	15.38898
NC_000079.7	41016719	Gcnt2	3.76E-07	13.73152
NC_000079.7	41393380	Gm32063	2.81E-19	10.03728
NC_000079.7	41393387	Gm32063	1.36E-19	14.1999
NC_000079.7	43459170	Gm52047	2.15E-07	28.34962
NC_000079.7	44568905	null	1.74E-11	-11.0002

NC_000079.7	44568923	null	5.05E-10	-10.7001
NC_000079.7	44568971	null	3.83E-14	-13.0743
NC_000079.7	44569050	null	6.29E-16	-13.2174
NC_000079.7	47379505	Rnf144b	1.87E-07	21.80769
NC_000079.7	47379550	Rnf144b	9.61E-07	23.59082
NC_000079.7	47379670	Rnf144b	7.16E-07	24.2845
NC_000079.7	54966711	null	9.47E-07	18.17191
NC_000079.7	61733784	null	1.61E-07	-25.0307
NC_000079.7	93754068	Bhmt	3.47E-14	31.11625
NC_000079.7	93762463	Bhmt	3.96E-13	19.27996
NC_000079.7	95753068	F2r	1.56E-09	14.76696
NC_000079.7	95753089	F2r	3.54E-08	13.38086
NC_000079.7	95753096	F2r	6.3E-09	14.50016
NC_000079.7	98273282	Arhgef28	3.17E-09	-46.7402
NC_000079.7	1.02E+08	null	2.74E-24	19.45064
NC_000079.7	1.02E+08	Pik3r1	3.88E-16	15.39732
NC_000079.7	1.02E+08	Pik3r1	3.02E-20	18.24946
NC_000079.7	1.02E+08	Pik3r1	3.87E-10	22.17944
NC_000079.7	1.12E+08	null	9.01E-09	10.08786
NC_000079.7	1.2E+08	Tmem267	1.21E-07	-11.5823
NC_000079.7	1.2E+08	Tmem267	6.67E-10	-14.2626
NC_000079.7	1.2E+08	Tmem267	5.82E-09	-12.5151
NC_000080.7	3050869	null	4.17E-08	10.23359
NC_000080.7	28231270	Wnt5a	2.33E-11	11.57157
NC_000080.7	31126300	Sh3bp5	1.05E-07	38.49118
NC_000080.7	33673582	null	1.66E-12	-37.0541
NC_000080.7	33804603	A630023A22Rik	1.97E-22	23.2121
NC_000080.7	54437230	Tcra	5.37E-07	-11.2881
NC_000080.7	54681323	Mmp14	2.76E-08	15.24256
NC_000080.7	55384401	null	7.46E-11	10.58564
NC_000080.7	55384415	null	3.73E-10	11.71343
NC_000080.7	55384433	null	2.23E-29	24.35418
NC_000080.7	55384449	null	1.46E-14	16.83613
NC_000080.7	57121817	null	9.73E-07	-28.2212
NC_000080.7	57121829	null	7.86E-07	-32.7189
NC_000080.7	64494388	Msra	2.18E-08	-22.8624
NC_000080.7	76726264	Tsc22d1	1.09E-07	17.10001
NC_000080.7	76746386	Tsc22d1	8.98E-15	17.58483
NC_000080.7	76746394	Tsc22d1	3.5E-08	13.15873
NC_000080.7	1.21E+08	null	1.21E-10	-11.6135
NC_000081.7	54815166	Enpp2	5.95E-07	17.6258
NC_000081.7	59013937	null	1.69E-08	11.16491
NC_000081.7	76464269	Adck5	8.02E-20	-22.8636
NC_000081.7	76551234	Kifc2	5.53E-07	36.88699
NC_000081.7	78851957	Triobp	2.37E-14	17.31104
NC_000081.7	84495173	null	2.91E-10	-32.4226
NC_000081.7	85630993	Ppara	5.05E-07	22.7144

NC_000081.7	98933117	Tuba1c	3.68E-10	12.07777
NC_000081.7	1.01E+08	Slc4a8	1.56E-09	-26.6254
NC_000082.7	5655114	null	7.24E-09	54.13164
NC_000082.7	17320453	Aifm3	1.87E-07	-17.241
NC_000082.7	17903121	Prodh	2.62E-10	31.44439
NC_000082.7	23577134	null	6.97E-07	15.97157
NC_000082.7	34999729	Adcy5	6.65E-11	24.91513
NC_000082.7	49595873	Gm15518	9.01E-09	15.5977
NC_000082.7	87581523	null	1.69E-07	47.61024
NC_000082.7	89724750	Tiam1	1.35E-07	-12.2518
NC_000082.7	90082141	Scaf4	5.85E-11	-20.2995
NC_000082.7	91039768	Gm52261	1.6E-14	23.94041
NC_000082.7	93015112	null	5.43E-07	27.99851
NC_000083.7	22893044	Gm9805	3.17E-08	-16.6148
NC_000083.7	22893239	Gm9805	1.15E-13	-21.7647
NC_000083.7	22893520	Gm9805	8.72E-08	-18.4257
NC_000083.7	22893553	Gm9805	5.51E-07	-14.8042
NC_000083.7	22893654	Gm9805	7.17E-13	-22.813
NC_000083.7	23768034	n-TRcct2	2.6E-08	15.99793
NC_000083.7	26838766	Ergic1	1.49E-12	25.15914
NC_000083.7	28284626	Tcp11	4.59E-08	27.24298
NC_000083.7	28284684	Tcp11	1.83E-07	20.3522
NC_000083.7	34813192	Pbx2	2.26E-12	-29.8484
NC_000083.7	35133406	Slc44a4	5.07E-08	-16.9335
NC_000083.7	35133410	Slc44a4	3.76E-07	-14.8942
NC_000083.7	40154053	Gm26917	1.15E-10	28.05788
NC_000083.7	40158224	Gm26917	9.52E-09	29.70417
NC_000083.7	40158227	Gm26917	1.23E-15	38.67208
NC_000083.7	40158234	Gm26917	7.27E-15	37.43948
NC_000083.7	40158244	Gm26917	4.08E-15	38.46985
NC_000083.7	40158282	Gm26917	1.36E-13	32.45559
NC_000083.7	40158289	Gm26917	7.12E-19	42.39929
NC_000083.7	40158297	Gm26917	5.93E-19	42.84762
NC_000083.7	40158303	Gm26917	1.83E-14	37.27365
NC_000083.7	40158314	Gm26917	1.18E-11	31.50523
NC_000083.7	40158335	Gm26917	4.92E-16	40.0536
NC_000083.7	40158341	Gm26917	1.13E-11	29.99047
NC_000083.7	40158353	Gm26917	9.34E-14	35.78102
NC_000083.7	40158356	Gm26917	1.01E-15	37.48642
NC_000083.7	40158359	Gm26917	8.98E-12	31.16711
NC_000083.7	40158375	Gm26917	1.5E-15	36.46317
NC_000083.7	40159337	Gm26917	1.27E-17	10.93526
NC_000083.7	40159339	Gm26917	7.61E-12	10.24861
NC_000083.7	40159432	Gm26917	4.13E-07	13.25419
NC_000083.7	47323802	Ubr2	3.2E-08	14.70323
NC_000083.7	56478632	Tnfaip8l1	1.07E-07	27.27892
NC_000083.7	56478680	Tnfaip8l1	1.82E-08	27.42101

NC_000083.7	56478696	Tnfaip8l1	1.33E-07	24.75794
NC_000084.7	18026444	Gm54037	5.07E-10	-17.3472
NC_000084.7	33685151	null	9.89E-07	46.9571
NC_000084.7	33685178	null	3E-07	50.30667
NC_000084.7	33685207	null	5.07E-09	56.23029
NC_000084.7	37887153	Gm37013	1.51E-08	23.41647
NC_000084.7	37887185	Gm37013	3.56E-07	19.71041
NC_000084.7	38411020	Pcdh12	2.17E-07	39.97972
NC_000084.7	39496200	Arhgap26	1.2E-10	20.4573
NC_000084.7	39496201	Arhgap26	3.17E-14	20.48284
NC_000084.7	39496219	Arhgap26	9.94E-08	14.67509
NC_000084.7	39496224	Arhgap26	2.44E-13	19.41445
NC_000084.7	39496242	Arhgap26	6.06E-18	23.16412
NC_000084.7	39496249	Arhgap26	2.56E-08	14.98282
NC_000084.7	39496255	Arhgap26	8.16E-21	23.94197
NC_000084.7	39496356	Arhgap26	5.3E-09	18.31498
NC_000084.7	39496401	Arhgap26	9.05E-14	21.5271
NC_000084.7	56684372	Aldh7a1	2.82E-07	27.63246
NC_000084.7	72864499	null	4.09E-09	-18.2397
NC_000084.7	75556194	null	8.54E-10	-29.0089
NC_000084.7	80212969	null	3.23E-11	12.05501
NC_000084.7	80212993	null	4.3E-07	11.62652
NC_000084.7	81242412	null	8.58E-31	20.75849
NC_000084.7	81242421	null	1.13E-34	21.56669
NC_000084.7	81242540	null	7.13E-35	23.25037
NC_000084.7	81242544	null	5.35E-34	24.47861
NC_000084.7	84311125	Zfp407	6.64E-07	19.3203
NC_000084.7	84311158	Zfp407	1.81E-07	19.72703
NC_000084.7	84311216	Zfp407	4.86E-11	23.42068
NC_000085.7	3809972	null	2.24E-10	20.82806
NC_000085.7	5709387	Sipa1	5.34E-08	-22.4683
NC_000085.7	25014506	Dock8	3.46E-09	26.57066
NC_000085.7	37691281	Cyp26a1	1.32E-09	16.00458
NC_000085.7	38503299	Plce1	3.3E-09	19.51866
NC_000085.7	44386271	Scd1	1.15E-14	-17.3373
NC_000085.7	44386378	Scd1	2.3E-13	-17.6914
NC_000085.7	44386379	Scd1	2.33E-41	-27.9355
NC_000085.7	44386432	Scd1	4.86E-11	-10.6834
NC_000085.7	44387996	Scd1	0	-21.8181
NC_000085.7	44388031	Scd1	1.69E-35	-12.6723
NC_000085.7	47501478	Stn1	5.14E-17	21.53309
NC_000085.7	47501570	Stn1	9.9E-11	14.59656
NC_000085.7	47501614	Stn1	4.53E-09	15.20831
NC_000085.7	53331912	Mxi1	4.68E-35	14.39232
NC_000085.7	53393147	null	9.39E-09	15.67868
NC_000085.7	53393215	null	1.83E-09	16.2921
NC_000085.7	53545646	Gm54049	2.37E-10	19.12396

NC_000085.7	55903423	Tcf7l2	2.98E-10	10.84675
NC_000085.7	55903438	Tcf7l2	3.49E-13	12.24341
NC_000085.7	55903452	Tcf7l2	7E-15	14.06677
NC_000085.7	58358489	Gfra1	1.27E-19	14.63397
NC_000085.7	58441629	Gfra1	3.78E-18	43.69145
NC_000085.7	58441659	Gfra1	3.47E-10	29.53485
NC_000086.8	11995708	Bcor	8.41E-08	-39.5199
NC_000086.8	11995725	Bcor	3.47E-08	-40.7699
NC_000086.8	11995727	Bcor	3.47E-08	-40.7699
NC_000086.8	11995733	Bcor	3.47E-08	-40.7699
NC_000086.8	11995737	Bcor	8.78E-09	-48.744
NC_000086.8	11995806	Bcor	2.19E-08	-46.4713
NC_000086.8	11995835	Bcor	2.61E-07	-33.7253
NC_000086.8	72125995	Xlr4a	3.76E-07	17.79381
NC_000086.8	72126011	Xlr4a	6.5E-09	19.93609
NC_000086.8	72126019	Xlr4a	6.02E-07	18.74954
NC_000086.8	72126023	Xlr4a	4.26E-08	17.27425
NC_000086.8	72126042	Xlr4a	2.22E-09	16.67308
NC_000086.8	72258041	Xlr4b	6.41E-11	16.97001
NC_000086.8	72286612	Xlr4c	2E-18	26.41113
NC_000086.8	72286616	Xlr4c	4.05E-10	18.80892
NC_000086.8	1.01E+08	Gcna	5.71E-09	28.96423
NT_166281.1	116091	LOC100503923	5.43E-11	25.01473
NT_166281.1	116094	LOC100503923	8.63E-16	30.87958
**CTR vs CTR+beer**				
**Chromosome**	**Start**	**Gene**	**FDR**	**Meth Difference**
NC_000067.7	24046882	null	1.98E-12	27.14121
NC_000067.7	59386391	Cdk15	6.25E-07	21.51318
NC_000067.7	75464045	Obsl1	6.43E-13	12.02597
NC_000067.7	75464067	Obsl1	9.92E-12	11.03588
NC_000067.7	75464075	Obsl1	6.64E-10	10.2297
NC_000067.7	75464078	Obsl1	6.75E-12	11.42994
NC_000067.7	75464080	Obsl1	4.69E-15	13.49508
NC_000067.7	75464099	Obsl1	4.96E-11	11.16717
NC_000067.7	75464101	Obsl1	7.55E-11	10.93964
NC_000067.7	75464132	Obsl1	1.99E-13	12.1979
NC_000067.7	90130660	Ackr3	1.63E-08	16.49506
NC_000067.7	1.32E+08	Nuak2	1.55E-13	37.47413
NC_000067.7	1.37E+08	Gm41958	7.35E-07	27.93684
NC_000067.7	1.51E+08	Hmcn1	9.24E-07	20.78346
NC_000067.7	1.51E+08	Hmcn1	2.8E-08	23.91231
NC_000067.7	1.55E+08	Cacna1e	8.67E-07	-10.5362
NC_000067.7	1.55E+08	null	2.67E-10	38.58635
NC_000067.7	1.78E+08	Catspere2	3.79E-11	-25.6953
NC_000067.7	1.78E+08	Catspere2	6.45E-08	-22.6623
NC_000067.7	1.79E+08	Kif26b	5.42E-12	30.8823
NC_000067.7	1.81E+08	Degs1l	6.35E-11	13.40047

NC_000068.8	29758791	Cercam	1.31E-12	15.21527
NC_000068.8	29758795	Cercam	5.66E-07	10.81653
NC_000068.8	34924782	Hc	1.05E-11	24.42397
NC_000068.8	67852037	B3galt1	8.1E-10	31.4166
NC_000068.8	72047363	Rapgef4	2.39E-07	-19.8757
NC_000068.8	1.03E+08	Cd44	8.82E-08	-20.6255
NC_000068.8	1.16E+08	Cdin1	4.99E-07	-31.0957
NC_000068.8	1.22E+08	Gm34325	7.5E-08	-28.5681
NC_000068.8	1.22E+08	Gm34325	3.37E-11	-29.2255
NC_000068.8	1.22E+08	Gm34325	9.9E-10	-21.5657
NC_000068.8	1.31E+08	Atrn	6.51E-09	22.84993
NC_000068.8	1.38E+08	Btbd3	8.9E-09	32.5786
NC_000068.8	1.38E+08	Btbd3	5.37E-08	30.15924
NC_000068.8	1.38E+08	Btbd3	5.14E-08	30.19765
NC_000068.8	1.38E+08	Btbd3	6.66E-10	31.82481
NC_000068.8	1.42E+08	Macrod2	1.75E-09	22.03421
NC_000068.8	1.48E+08	9030622O22Rik	1.23E-15	16.71363
NC_000068.8	1.48E+08	9030622O22Rik	9.46E-14	14.64508
NC_000068.8	1.52E+08	Rbck1	3.62E-11	19.17304
NC_000068.8	1.53E+08	Nol4l	9.34E-08	-31.1518
NC_000068.8	1.63E+08	Tox2	1.06E-08	21.36007
NC_000068.8	1.63E+08	Hnf4a	2.97E-11	10.62387
NC_000068.8	1.68E+08	Nfatc2	3.04E-09	28.30462
NC_000068.8	1.71E+08	Dok5	5.81E-08	16.18595
NC_000069.7	18106447	null	1.83E-08	13.99053
NC_000069.7	27334395	Gm42192	1.1E-09	16.04785
NC_000069.7	36907416	null	1.15E-10	33.98655
NC_000069.7	36907418	null	8.2E-08	26.91149
NC_000069.7	36907450	null	4.71E-08	27.5706
NC_000069.7	36907470	null	6.54E-10	31.44672
NC_000069.7	36907500	null	3.45E-09	31.64141
NC_000069.7	38511541	Ankrd50	9.05E-10	-13.0502
NC_000069.7	38511555	Ankrd50	2.71E-07	-10.3905
NC_000069.7	41363583	null	2.77E-07	30.27938
NC_000069.7	52508228	null	1.51E-08	39.83437
NC_000069.7	53391390	null	1.27E-07	11.80526
NC_000069.7	53391398	null	5.37E-09	14.30291
NC_000069.7	67369961	Gfm1	3.72E-07	14.32121
NC_000069.7	79286719	Gm17359	2.55E-12	19.59918
NC_000069.7	79286756	Gm17359	7.25E-08	15.73599
NC_000069.7	79286863	Gm17359	3.49E-12	18.46601
NC_000069.7	79286872	Gm17359	5.59E-14	20.04506
NC_000069.7	97219442	null	3.59E-09	17.6018
NC_000069.7	97219457	null	3.25E-08	17.10136
NC_000069.7	97219513	null	3.32E-07	16.53889
NC_000069.7	1.01E+08	Ptgfrn	8.47E-08	-12.7868
NC_000069.7	1.2E+08	null	4.31E-09	-21.5075

NC_000069.7	1.22E+08	Bcar3	1.59E-10	29.15352
NC_000069.7	1.22E+08	Bcar3	1.34E-08	27.94706
NC_000069.7	1.28E+08	null	2.91E-10	10.7696
NC_000069.7	1.28E+08	null	1.84E-10	11.397
NC_000069.7	1.31E+08	Lef1	7.23E-07	24.94969
NC_000069.7	1.31E+08	Lef1	6.34E-07	23.39591
NC_000069.7	1.38E+08	Adh7	2.62E-07	30.07791
NC_000069.7	1.38E+08	Adh7	6.02E-07	25.62218
NC_000069.7	1.38E+08	Adh7	7.31E-07	26.25455
NC_000069.7	1.44E+08	Gm40161	2.01E-11	20.12123
NC_000070.7	40261373	Topors	1.38E-09	-12.1879
NC_000070.7	86556282	Plin2	1.06E-07	11.21461
NC_000070.7	97991130	Nfia	6.91E-07	16.10921
NC_000070.7	1.05E+08	C8a	1.66E-09	-12.4577
NC_000070.7	1.17E+08	Dynlt4	2.66E-07	-11.0164
NC_000070.7	1.28E+08	null	1.3E-09	30.20221
NC_000070.7	1.28E+08	null	3.33E-14	40.8366
NC_000070.7	1.28E+08	null	1.46E-08	32.44253
NC_000070.7	1.28E+08	null	3.61E-10	33.55777
NC_000070.7	1.29E+08	Tlr12	2.98E-08	10.45843
NC_000070.7	1.34E+08	Gm34296	2.55E-24	15.32949
NC_000070.7	1.34E+08	Gm34296	2.46E-12	11.22307
NC_000070.7	1.36E+08	Hmgcl	4.64E-08	19.25293
NC_000070.7	1.39E+08	Gm16287	6.93E-16	-21.4335
NC_000070.7	1.39E+08	Gm16287	4.93E-08	-12.8239
NC_000070.7	1.39E+08	Akr7a5	4.81E-08	15.52893
NC_000070.7	1.39E+08	Iffo2	1.89E-08	14.75229
NC_000070.7	1.39E+08	Iffo2	2.37E-07	12.29116
NC_000070.7	1.42E+08	Efhd2	3.9E-08	27.22181
NC_000070.7	1.46E+08	null	2.59E-08	35.66216
NC_000070.7	1.49E+08	Gm572	1.34E-07	12.00347
NC_000070.7	1.49E+08	Casz1	5.99E-07	23.96763
NC_000070.7	1.49E+08	Casz1	3.49E-08	24.66979
NC_000070.7	1.5E+08	Clstn1	4.14E-09	20.89619
NC_000070.7	1.5E+08	Clstn1	1.16E-09	25.51794
NC_000070.7	1.5E+08	Clstn1	2.98E-07	19.58233
NC_000070.7	1.5E+08	Clstn1	2.45E-08	15.22987
NC_000070.7	1.5E+08	Clstn1	1.28E-08	21.67335
NC_000070.7	1.5E+08	Clstn1	5.02E-07	18.67462
NC_000070.7	1.55E+08	Prkcz	1E-09	11.76567
NC_000070.7	1.56E+08	Nadk	3.02E-09	14.03327
NC_000070.7	1.56E+08	Nadk	9.28E-13	16.28071
NC_000070.7	1.56E+08	Nadk	4.69E-12	15.29277
NC_000070.7	1.56E+08	Nadk	1.52E-14	16.13281
NC_000070.7	1.56E+08	Nadk	2.12E-13	15.36097
NC_000071.7	5315390	Cdk14	2.58E-07	23.16451
NC_000071.7	36326734	Sorcs2	6.83E-08	12.59665

NC_000071.7	40952587	null	8.07E-07	16.02124
NC_000071.7	54447883	null	1.47E-09	12.30818
NC_000071.7	67805092	Atp8a1	5.74E-07	21.01346
NC_000071.7	77005932	Cracd	4.01E-08	18.08327
NC_000071.7	77005938	Cracd	1.57E-12	20.93871
NC_000071.7	92512900	Art3	1.2E-11	24.68092
NC_000071.7	92512910	Art3	3.02E-09	22.06318
NC_000071.7	92512919	Art3	5.41E-10	22.34762
NC_000071.7	92745191	null	1.11E-07	-21.1898
NC_000071.7	92747064	null	2.05E-10	11.56369
NC_000071.7	92747071	null	4.15E-10	11.1418
NC_000071.7	92747083	null	1.55E-11	10.9647
NC_000071.7	92747157	null	9.43E-09	10.11451
NC_000071.7	1.05E+08	Spp1	3.73E-10	23.80413
NC_000071.7	1.05E+08	Pkd2	3.69E-13	15.71843
NC_000071.7	1.13E+08	1700016C19Rik	4.27E-08	-14.5777
NC_000071.7	1.13E+08	1700016C19Rik	1.04E-07	-14.7427
NC_000071.7	1.15E+08	null	1.27E-09	36.4802
NC_000071.7	1.15E+08	null	1.08E-13	49.61677
NC_000071.7	1.15E+08	Fam222a	3.76E-07	15.12885
NC_000071.7	1.15E+08	Cabp1	2.4E-07	-24.4723
NC_000071.7	1.16E+08	Cit	3.2E-10	12.58362
NC_000071.7	1.16E+08	Cit	1.52E-08	12.77462
NC_000071.7	1.16E+08	Cit	6.12E-09	14.13581
NC_000071.7	1.16E+08	Cit	7.99E-11	15.03368
NC_000071.7	1.16E+08	Cit	1.46E-09	14.24401
NC_000071.7	1.16E+08	Cit	1.69E-08	13.49699
NC_000071.7	1.16E+08	Cit	2.28E-12	15.62129
NC_000071.7	1.21E+08	Tpcn1	6.24E-07	15.37129
NC_000071.7	1.22E+08	Sh2b3	2.88E-10	28.68679
NC_000071.7	1.22E+08	Sh2b3	2.15E-15	34.20552
NC_000071.7	1.22E+08	Sh2b3	1.91E-13	33.86875
NC_000071.7	1.22E+08	Sh2b3	1.66E-14	33.09635
NC_000071.7	1.22E+08	Cux2	5.5E-07	-22.576
NC_000071.7	1.23E+08	Camkk2	1.26E-09	15.20824
NC_000071.7	1.23E+08	Camkk2	2.09E-10	15.48254
NC_000071.7	1.23E+08	Camkk2	8.99E-07	12.62207
NC_000071.7	1.23E+08	Camkk2	4.12E-10	15.142
NC_000071.7	1.35E+08	Mlxipl	4.77E-07	21.2089
NC_000071.7	1.38E+08	Agfg2	8.21E-08	20.14925
NC_000071.7	1.38E+08	Agfg2	7.21E-12	24.94307
NC_000071.7	1.38E+08	Agfg2	1.81E-09	22.40635
NC_000071.7	1.38E+08	Mepce	2.56E-07	-44.4429
NC_000071.7	1.49E+08	Gm35620	2.65E-11	10.84844
NC_000071.7	1.49E+08	Gm35620	2.82E-07	10.5331
NC_000072.7	6184141	Slc25a13	6.66E-11	12.79608
NC_000072.7	18845949	null	1.01E-11	10.05346

NC_000072.7	28928070	Snd1	1.12E-08	23.29507
NC_000072.7	28928078	Snd1	1.48E-07	21.53699
NC_000072.7	28928088	Snd1	3.59E-08	24.50469
NC_000072.7	28928245	Snd1	2.16E-07	19.42341
NC_000072.7	28928258	Snd1	2.48E-07	19.48871
NC_000072.7	31077228	Lncpint	7.99E-10	11.56972
NC_000072.7	34974800	null	1.4E-10	10.52352
NC_000072.7	34974852	null	1.26E-14	15.02689
NC_000072.7	38035580	Atp6v0a4	7.65E-08	10.28924
NC_000072.7	52177359	Hoxa3	1.6E-09	22.93711
NC_000072.7	52876046	Jazf1	1.03E-07	18.91657
NC_000072.7	82671864	Gm32425	2.54E-07	10.99571
NC_000072.7	82671881	Gm32425	2.6E-07	12.94301
NC_000072.7	82671890	Gm32425	3.88E-07	11.78877
NC_000072.7	82671936	Gm32425	5.21E-10	13.3202
NC_000072.7	82671942	Gm32425	4.18E-12	13.87948
NC_000072.7	1.16E+08	Plxnd1	6.85E-10	-47.6352
NC_000072.7	1.19E+08	Cacna1c	2.72E-07	25.21697
NC_000072.7	1.19E+08	Cacna1c	3.16E-07	22.63839
NC_000072.7	1.19E+08	Cacna1c	2.29E-08	31.71285
NC_000072.7	1.21E+08	Mical3	3.89E-07	19.44005
NC_000072.7	1.43E+08	null	1.99E-08	28.38052
NC_000073.7	15667459	null	8.08E-09	20.367
NC_000073.7	24792171	Pou2f2	2.03E-07	-28.55
NC_000073.7	30810391	Hpn	3.99E-07	18.4141
NC_000073.7	45262530	Fgf21	4.25E-14	10.88198
NC_000073.7	62069755	Gm32061	1.36E-08	23.28472
NC_000073.7	62069887	Gm32061	8.28E-07	21.46725
NC_000073.7	80106222	Blm	6.25E-08	15.28465
NC_000073.7	80106275	Blm	2.89E-07	22.52035
NC_000073.7	80106276	Blm	7.76E-09	14.92571
NC_000073.7	81383538	Gm42400	7.84E-09	20.05713
NC_000073.7	83304061	Il16	1.77E-09	-35.4772
NC_000073.7	1.09E+08	Gm32563	5.37E-07	28.51813
NC_000073.7	1.12E+08	Gm39071	3.23E-08	37.56784
NC_000073.7	1.12E+08	Parva	1.35E-13	22.87927
NC_000073.7	1.12E+08	Parva	9.9E-07	17.63482
NC_000073.7	1.12E+08	Parva	6.24E-15	24.38455
NC_000073.7	1.13E+08	null	6.8E-10	20.7489
NC_000073.7	1.13E+08	null	3.59E-10	21.947
NC_000073.7	1.25E+08	Il4ra	7.52E-07	19.5103
NC_000073.7	1.31E+08	Htra1	9.26E-07	10.39182
NC_000073.7	1.41E+08	null	4.78E-08	21.92009
NC_000074.7	10904123	Gm39130	2.24E-08	14.89578
NC_000074.7	31657764	Mak16	1.13E-07	16.50275
NC_000074.7	35089299	B930018H19Rik	2.8E-07	11.5486
NC_000074.7	35089450	B930018H19Rik	8.57E-09	14.35723

NC_000074.7	35883829	Gm34911	7.89E-08	17.01914
NC_000074.7	35883863	Gm34911	3.95E-07	32.8136
NC_000074.7	35883984	Gm34911	3.64E-08	31.33768
NC_000074.7	35884047	Gm34911	2.71E-10	32.86732
NC_000074.7	36588986	Prag1	2.51E-08	27.12316
NC_000074.7	45428282	Fat1	2.25E-07	20.19452
NC_000074.7	71217718	6330537M06Rik	2.04E-11	16.71081
NC_000074.7	92264347	Fto	1.37E-25	27.80107
NC_000074.7	95821459	Katnb1	7.06E-07	-14.566
NC_000074.7	95855361	Kifc3	2.48E-08	18.1491
NC_000074.7	1.12E+08	Bcar1	2.04E-08	21.13342
NC_000074.7	1.16E+08	Wwox	4.6E-07	-16.1392
NC_000074.7	1.16E+08	Wwox	2.72E-07	-17.2729
NC_000074.7	1.2E+08	Cdh13	6E-09	13.78117
NC_000074.7	1.21E+08	Cibar2	4.94E-07	21.09596
NC_000074.7	1.21E+08	Cibar2	8.78E-07	21.39401
NC_000074.7	1.21E+08	A330074K22Rik	1.49E-07	24.44204
NC_000075.7	4259516	null	1.79E-07	20.22835
NC_000075.7	4259547	null	3.08E-08	19.06175
NC_000075.7	21079549	Gm36118	3.98E-14	43.0539
NC_000075.7	22552448	Bbs9	8.56E-07	17.97764
NC_000075.7	22916973	Gm36743	4.49E-07	15.29091
NC_000075.7	42585136	Grik4	4.31E-15	28.02547
NC_000075.7	42585156	Grik4	4.79E-12	23.05756
NC_000075.7	44619948	Phldb1	5.28E-13	11.07716
NC_000075.7	44620001	Phldb1	1.12E-13	11.09526
NC_000075.7	48672553	Zbtb16	1.18E-07	13.15208
NC_000075.7	53251797	Exph5	1.95E-08	14.82673
NC_000075.7	55451663	Isl2	4.36E-07	20.52493
NC_000075.7	59509188	Celf6	5.74E-18	19.02478
NC_000075.7	59509208	Celf6	3.3E-11	18.45164
NC_000075.7	63662598	Smad3	1.8E-07	11.27761
NC_000075.7	63662668	Smad3	8.96E-07	10.87615
NC_000075.7	63788155	null	6.98E-07	36.98727
NC_000075.7	83713334	null	1.39E-10	10.22264
NC_000075.7	1.18E+08	Rbms3	1.44E-08	27.0786
NC_000075.7	1.21E+08	Ulk4	1.38E-07	-33.3804
NC_000076.7	20042730	Map7	7.59E-09	25.16087
NC_000076.7	61664286	Gm51765	2.05E-07	-21.5956
NC_000076.7	69084635	Rhobtb1	6.31E-07	30.05935
NC_000076.7	77270448	null	1.59E-08	-10.5156
NC_000076.7	86827177	Stab2	6.35E-09	-41.2344
NC_000076.7	95250576	Socs2	3.88E-07	18.50243
NC_000076.7	95250615	Socs2	1.13E-08	19.50216
NC_000076.7	95250622	Socs2	1.72E-07	17.29587
NC_000076.7	95250628	Socs2	4.23E-08	19.85378
NC_000076.7	95250654	Socs2	9.44E-10	22.31617

NC_000076.7	95250659	Socs2	3.26E-08	30.14913
NC_000076.7	95250664	Socs2	9.44E-10	22.31617
NC_000076.7	95250680	Socs2	1.97E-07	15.51082
NC_000076.7	95250709	Socs2	3.97E-08	18.32647
NC_000076.7	95250711	Socs2	4.72E-08	18.05132
NC_000076.7	95250722	Socs2	1.24E-08	18.41909
NC_000076.7	95250729	Socs2	1.79E-07	17.31295
NC_000076.7	95250731	Socs2	6E-10	30.45109
NC_000076.7	95911895	Gm49817	5.11E-07	27.09251
NC_000076.7	1.11E+08	null	2.43E-07	12.88786
NC_000076.7	1.21E+08	null	7.52E-12	21.40445
NC_000076.7	1.21E+08	null	1.24E-11	20.93169
NC_000076.7	1.21E+08	null	1.59E-10	19.63851
NC_000076.7	1.21E+08	null	1.68E-07	17.3584
NC_000076.7	1.27E+08	Ddit3	5.74E-10	10.98148
NC_000076.7	1.28E+08	Gpr182	5.75E-07	-21.1709
NC_000077.7	5958867	Camk2b	1.71E-23	24.76152
NC_000077.7	5958876	Camk2b	1.77E-26	26.3407
NC_000077.7	5958883	Camk2b	2.13E-26	25.99859
NC_000077.7	5958950	Camk2b	1.04E-29	26.7007
NC_000077.7	5958958	Camk2b	1.01E-25	25.22173
NC_000077.7	5958975	Camk2b	9.07E-27	25.52662
NC_000077.7	5959006	Camk2b	3.26E-26	25.71205
NC_000077.7	22922036	Commd1	1.85E-07	30.27096
NC_000077.7	22922054	Commd1	2.71E-07	31.8426
NC_000077.7	22922082	Commd1	1.88E-07	32.73628
NC_000077.7	22922093	Commd1	6.66E-08	35.34712
NC_000077.7	22922112	Commd1	2.04E-07	32.37008
NC_000077.7	22922120	Commd1	2.62E-07	32.66082
NC_000077.7	22923934	Commd1	1.95E-11	18.64296
NC_000077.7	22923999	Commd1	3.67E-07	15.07907
NC_000077.7	22924027	Commd1	7.03E-08	15.79201
NC_000077.7	29475944	Mtif2	1.6E-11	14.43764
NC_000077.7	60707972	null	2.78E-08	16.568
NC_000077.7	60708058	null	1.13E-09	19.78889
NC_000077.7	60708088	null	1.74E-10	23.27689
NC_000077.7	70117548	Bcl6b	3.58E-08	15.50298
NC_000077.7	70117572	Bcl6b	4.66E-21	30.89224
NC_000077.7	95662536	null	4.62E-07	-33.1505
NC_000077.7	97029955	Tbkbp1	8.21E-07	18.90121
NC_000077.7	1E+08	Krt19	6.55E-09	16.74134
NC_000077.7	1.13E+08	null	6.16E-08	13.98883
NC_000077.7	1.16E+08	null	1.09E-09	21.29275
NC_000077.7	1.17E+08	Septin9	1.33E-07	28.27224
NC_000077.7	1.17E+08	Septin9	2.47E-08	31.20493
NC_000077.7	1.17E+08	Septin9	3.61E-08	21.13946
NC_000077.7	1.17E+08	Septin9	2.05E-07	37.38388

NC_000077.7	1.18E+08	Dnah17	8.45E-08	-32.4919
NC_000077.7	1.18E+08	Dnah17	5.58E-09	-27.4942
NC_000078.7	11744879	null	1.96E-07	14.27288
NC_000078.7	16591722	Lpin1	2.06E-08	19.55581
NC_000078.7	21911546	null	3.39E-08	13.67982
NC_000078.7	31244649	null	1.75E-07	-17.2643
NC_000078.7	39842471	null	5.53E-11	14.97137
NC_000078.7	80217340	Actn1	6.84E-07	15.86279
NC_000078.7	91017422	Cep128	1.25E-07	18.76372
NC_000078.7	91017437	Cep128	3.96E-12	23.09058
NC_000078.7	91017460	Cep128	1.64E-07	19.22842
NC_000078.7	91383283	Tshr	8.32E-07	20.14404
NC_000078.7	1.06E+08	null	1.56E-10	15.42713
NC_000079.7	8340078	Adarb2	4.07E-11	14.36535
NC_000079.7	46074617	Atxn1	5.29E-08	28.80383
NC_000079.7	56237710	Macroh2a1	2.3E-07	29.55345
NC_000079.7	73910646	Slc12a7	9.59E-07	14.49901
NC_000079.7	76010468	null	6.51E-10	15.65226
NC_000079.7	1.03E+08	null	2.69E-08	33.43185
NC_000079.7	1.03E+08	Mast4	5.57E-12	16.34605
NC_000079.7	1.14E+08	Snx18	2.45E-09	28.90867
NC_000079.7	1.2E+08	Tmem267	8.19E-08	-12.122
NC_000080.7	28231203	Wnt5a	1.01E-10	11.31784
NC_000080.7	28231259	Wnt5a	2.31E-13	16.41348
NC_000080.7	28231270	Wnt5a	8.8E-13	15.00743
NC_000080.7	33673582	null	8.46E-15	-38.5897
NC_000080.7	55384401	null	3.55E-10	10.63135
NC_000080.7	55384433	null	1.31E-14	18.58987
NC_000080.7	64494388	Msra	2.19E-07	-18.7872
NC_000080.7	64494390	Msra	7.99E-07	-15.2085
NC_000080.7	72205286	null	5.93E-08	15.75245
NC_000080.7	76388970	n-TEttc6	5.64E-21	15.38348
NC_000080.7	1.19E+08	Abcc4	2.52E-08	-29.6102
NC_000081.7	25440594	Gm48957	1.45E-07	17.72042
NC_000081.7	25440599	Gm48957	2.28E-07	17.835
NC_000081.7	25440604	Gm48957	3.19E-10	20.24369
NC_000081.7	59013937	null	9.34E-12	13.45395
NC_000081.7	80781173	Tnrc6b	7.46E-10	12.95496
NC_000081.7	80781223	Tnrc6b	8.1E-08	12.156
NC_000081.7	82956801	null	4.15E-07	-14.7871
NC_000081.7	83581683	Scube1	8.82E-09	13.40773
NC_000081.7	94432948	Pus7l	5.82E-08	11.95031
NC_000081.7	98276105	Or8s2	2.11E-09	-27.145
NC_000081.7	1.01E+08	Slc4a8	6.7E-10	-25.6085
NC_000081.7	1.02E+08	Tns2	1.7E-18	25.10696
NC_000081.7	1.02E+08	Tns2	3.11E-07	15.06906
NC_000081.7	1.02E+08	Tns2	1.38E-08	16.40824

NC_000081.7	1.02E+08	Tns2	1.75E-09	15.09792
NC_000081.7	1.02E+08	Tns2	1.22E-08	15.19239
NC_000081.7	1.02E+08	Tns2	9.57E-10	13.01255
NC_000081.7	1.02E+08	Tns2	7.32E-09	14.00861
NC_000081.7	1.02E+08	Tns2	2E-09	14.03827
NC_000081.7	1.02E+08	Tns2	5.22E-11	16.21822
NC_000082.7	4236725	Adcy9	1.01E-09	15.13033
NC_000082.7	4536440	Nmral1	2.04E-09	13.5041
NC_000082.7	4536533	Nmral1	4.69E-10	12.61908
NC_000082.7	4536550	Nmral1	4.19E-08	10.89533
NC_000082.7	4536561	Nmral1	2.21E-12	14.54781
NC_000082.7	4536594	Nmral1	6.43E-08	10.9242
NC_000082.7	4536604	Nmral1	6.61E-09	11.78335
NC_000082.7	10601990	Socs1	4.98E-09	15.79679
NC_000082.7	17320478	Aifm3	5.09E-09	-21.4295
NC_000082.7	30899126	Xxylt1	2.81E-08	15.48518
NC_000082.7	31116206	Apod	9.13E-07	16.47892
NC_000082.7	37674823	Btnl12	7.16E-07	12.60344
NC_000082.7	43067583	Zbtb20	3.91E-11	10.81301
NC_000082.7	57211639	Cmss1	0	12.09035
NC_000082.7	57211651	Cmss1	0	11.59216
NC_000082.7	57211658	Cmss1	0	10.30147
NC_000082.7	57211699	Cmss1	0	11.17897
NC_000082.7	57211725	Cmss1	0	10.81504
NC_000082.7	57211785	Cmss1	0	11.17194
NC_000082.7	57211814	Cmss1	2.33E-42	10.35266
NC_000082.7	65918303	null	1.69E-09	-25.9032
NC_000082.7	76477084	null	8.09E-07	13.1549
NC_000082.7	90534400	Mrap	2.96E-15	14.4785
NC_000082.7	90534404	Mrap	1.4E-07	10.10939
NC_000083.7	15602078	LOC118568318	2.14E-11	11.49405
NC_000083.7	29738864	Tmem217	2.99E-07	12.2866
NC_000083.7	31544074	Slc37a1	4.7E-09	19.18724
NC_000083.7	34135805	Zbtb22	3.71E-09	23.46619
NC_000083.7	34170322	B3galt4	1.53E-08	-26.1546
NC_000083.7	34170334	B3galt4	4.3E-08	-24.4243
NC_000083.7	34170387	B3galt4	1.76E-09	-24.901
NC_000083.7	34170447	B3galt4	1.25E-07	-42.7696
NC_000083.7	34170455	B3galt4	6.64E-09	-46.1746
NC_000083.7	34170465	B3galt4	8.37E-07	-40.4251
NC_000083.7	34170475	B3galt4	7.9E-08	-42.689
NC_000083.7	34813192	Pbx2	5.26E-09	-24.1795
NC_000083.7	40159337	Gm26917	1.86E-13	12.14099
NC_000083.7	40159339	Gm26917	4.02E-11	10.57079
NC_000083.7	40159432	Gm26917	3.44E-07	15.18445
NC_000083.7	44815666	Runx2	2.5E-07	12.06642
NC_000083.7	46004143	Mrpl14	5E-07	11.80356

NC_000083.7	46004239	Mrpl14	8.85E-07	15.34927
NC_000083.7	46004259	Mrpl14	1.87E-08	14.89438
NC_000083.7	46361818	null	5.42E-08	14.28759
NC_000083.7	50117221	Kif6	7.5E-07	10.85483
NC_000083.7	56314290	Stap2	2.7E-09	19.4554
NC_000083.7	56314319	Stap2	6.77E-07	16.07727
NC_000083.7	56314435	Stap2	1.12E-08	18.34242
NC_000083.7	56317475	Mpnd	9.17E-10	16.17042
NC_000083.7	56317524	Mpnd	1.24E-11	22.49041
NC_000083.7	56317566	Mpnd	2.9E-08	18.43176
NC_000083.7	56478632	Tnfaip8l1	7.09E-08	24.88973
NC_000083.7	71199732	Gm26510	5.94E-08	11.74007
NC_000083.7	74193380	Xdh	7.38E-12	20.49582
NC_000083.7	74193388	Xdh	3.26E-14	23.20137
NC_000083.7	74193430	Xdh	1.46E-11	20.73146
NC_000083.7	74193441	Xdh	3.17E-07	16.33387
NC_000083.7	78714503	Fez2	1.69E-10	28.19739
NC_000083.7	85095991	Lrpprc	1.47E-09	16.38979
NC_000083.7	86795608	Prkce	1.69E-08	11.95568
NC_000083.7	88866166	Ppp1r21	5.62E-08	30.86043
NC_000084.7	35786403	Prob1	2.82E-07	-19.3484
NC_000084.7	35786422	Prob1	4.47E-07	-18.4893
NC_000084.7	35786468	Prob1	1.6E-07	-13.9092
NC_000084.7	37870449	Gm37013	3.41E-08	17.29622
NC_000084.7	37871041	Gm37013	1.66E-10	10.93006
NC_000084.7	37880531	Gm37013	4.79E-10	16.48386
NC_000084.7	60737434	Synpo	4.15E-08	24.91148
NC_000084.7	60737511	Synpo	7.47E-07	23.87026
NC_000084.7	60737531	Synpo	1.72E-07	22.37685
NC_000084.7	68195284	Ldlrad4	8.43E-08	19.66499
NC_000084.7	75013128	null	6.81E-10	14.20737
NC_000084.7	77879413	F830208F22Rik	2.28E-09	11.64702
NC_000084.7	81242412	null	1.6E-09	13.79606
NC_000084.7	84311216	Zfp407	2.84E-09	23.60666
NC_000085.7	5690883	Rela	4.03E-14	20.05134
NC_000085.7	5690911	Rela	5.01E-07	13.78346
NC_000085.7	5690921	Rela	2.83E-10	17.14016
NC_000085.7	5690939	Rela	8.01E-09	15.00853
NC_000085.7	6903463	Kcnk4	8.38E-08	26.56525
NC_000085.7	6904424	Kcnk4	3.27E-08	16.93731
NC_000085.7	6904430	Kcnk4	9.92E-08	18.36396
NC_000085.7	6904500	Kcnk4	4.16E-07	14.12573
NC_000085.7	6904561	Kcnk4	2.62E-11	19.81304
NC_000085.7	37691242	Cyp26a1	9.32E-10	19.32593
NC_000085.7	37691261	Cyp26a1	1.07E-11	21.19628
NC_000085.7	37691281	Cyp26a1	2.1E-12	17.16245
NC_000085.7	37691331	Cyp26a1	4.09E-08	14.44455

NC_000085.7	43878537	Dnmbp	7.95E-10	10.36738
NC_000085.7	43878547	Dnmbp	1.18E-10	10.24759
NC_000086.8	12000761	Bcor	1.62E-07	12.39787
NC_000086.8	1.49E+08	Tro	3.51E-08	15.13227
NC_000086.8	1.69E+08	Mid1	5.41E-08	-12.4513
NW_023337853.1	435	null	0	12.02944
NW_023337853.1	443	null	0	10.38947
NW_023337853.1	447	null	0	11.72611
NW_023337853.1	452	null	0	10.8858
NW_023337853.1	459	null	0	12.20454
NW_023337853.1	463	null	0	10.56613
NW_023337853.1	467	null	0	10.28574
NW_023337853.1	523	null	0	10.33809
NW_023337853.1	594	null	0	10.59194
NW_023337853.1	611	null	0	10.15701
NW_023337853.1	615	null	0	12.03003
NW_023337853.1	619	null	0	10.48664
NW_023337853.1	623	null	0	10.74696
NW_023337853.1	627	null	0	10.48155
NW_023337853.1	629	null	0	10.77113
NW_023337853.1	633	null	0	10.71315
NW_023337853.1	638	null	0	10.73817
NW_023337853.1	641	null	0	11.87486
NW_023337853.1	645	null	0	12.90075
NW_023337853.1	654	null	0	10.0739
**HFD vs HFD+beer**				
**Chromosome**	**Start**	**Gene**	**FDR**	**Meth Difference**
NC_000067.7	34478607	Ccdc115	1.63E-07	10.6212
NC_000067.7	34478609	Ccdc115	2.15E-08	12.98931
NC_000067.7	52205918	Gls	1.78E-10	20.90599
NC_000067.7	52205966	Gls	1.59E-12	27.7253
NC_000067.7	59522650	Fzd7	1.92E-09	16.58072
NC_000067.7	59523730	Fzd7	2.94E-10	30.14213
NC_000067.7	59523744	Fzd7	1.74E-07	27.32927
NC_000067.7	59523831	Fzd7	3.73E-07	28.88747
NC_000067.7	86452742	Ptma	9.17E-08	14.77443
NC_000067.7	90945340	Lrrfip1	4.22E-09	29.85552
NC_000067.7	1.36E+08	Phlda3	3.02E-07	-33.442
NC_000067.7	1.36E+08	null	1.33E-09	15.66241
NC_000067.7	1.57E+08	null	3.56E-08	29.91429
NC_000067.7	1.64E+08	Slc19a2	7.84E-08	17.78846
NC_000067.7	1.67E+08	null	2.55E-18	20.85439
NC_000067.7	1.67E+08	null	3.31E-13	17.53278
NC_000067.7	1.71E+08	Fcgr3	1.02E-08	25.52294
NC_000067.7	1.71E+08	Fcgr3	1.52E-08	20.62035
NC_000067.7	1.71E+08	Fcgr3	5.51E-07	18.93772
NC_000067.7	1.71E+08	Fcgr3	3.19E-07	20.45016

NC_000067.7	1.71E+08	Fcgr3	1.27E-08	20.30424
NC_000067.7	1.71E+08	Fcgr3	5.06E-09	21.68057
NC_000067.7	1.81E+08	null	2.32E-11	15.56999
NC_000067.7	1.81E+08	null	3.1E-09	12.36921
NC_000067.7	1.81E+08	null	9.16E-10	11.18102
NC_000067.7	1.81E+08	null	3.28E-07	10.31221
NC_000067.7	1.81E+08	null	6.94E-12	13.82899
NC_000067.7	1.81E+08	null	2.1E-09	12.43584
NC_000067.7	1.81E+08	null	4.24E-12	13.77523
NC_000067.7	1.81E+08	null	1.67E-08	10.32668
NC_000067.7	1.81E+08	null	6.62E-07	10.33042
NC_000067.7	1.84E+08	Hlx	6.16E-07	11.02837
NC_000067.7	1.84E+08	null	5.4E-09	11.44237
NC_000067.7	1.85E+08	Mtarc2	2.21E-09	34.10298
NC_000067.7	1.85E+08	Mtarc2	4.87E-09	26.12434
NC_000067.7	1.89E+08	Ush2a	2.05E-11	10.9836
NC_000068.8	11727700	Il15ra	9.26E-12	-30.8679
NC_000068.8	18069744	Mllt10	3.36E-09	34.76915
NC_000068.8	18069748	Mllt10	2.32E-09	34.76915
NC_000068.8	18069784	Mllt10	6.41E-09	30.00724
NC_000068.8	18069799	Mllt10	3.15E-07	25.95319
NC_000068.8	20904949	Arhgap21	4.65E-13	29.31263
NC_000068.8	20904953	Arhgap21	2.57E-07	19.04786
NC_000068.8	20904962	Arhgap21	9.43E-08	21.73573
NC_000068.8	20905000	Arhgap21	4.61E-11	21.37313
NC_000068.8	20905005	Arhgap21	2.47E-09	23.02445
NC_000068.8	30597754	null	2.02E-09	17.088
NC_000068.8	30597760	null	1.08E-07	16.29327
NC_000068.8	52269055	Gm13522	3.33E-08	27.25268
NC_000068.8	63486022	null	8.41E-08	-29.1605
NC_000068.8	72008965	Rapgef4	8.41E-08	25.66635
NC_000068.8	74760647	null	3.69E-07	24.67329
NC_000068.8	84496647	Btbd18	3.82E-07	19.11898
NC_000068.8	84496700	Btbd18	1.24E-07	19.62744
NC_000068.8	92261904	Cry2	1.92E-09	12.01459
NC_000068.8	1.03E+08	null	4.31E-08	36.83282
NC_000068.8	1.52E+08	Defb45	5.91E-08	-15.8717
NC_000068.8	1.57E+08	Blcap	3.28E-11	18.40995
NC_000068.8	1.57E+08	Blcap	3.86E-07	11.53087
NC_000068.8	1.57E+08	Blcap	1.71E-08	13.48936
NC_000068.8	1.63E+08	Serinc3	2.67E-09	22.24706
NC_000068.8	1.73E+08	null	2.09E-09	22.65325
NC_000069.7	9674387	Zfp704	5.04E-07	18.05148
NC_000069.7	28897349	null	2.9E-10	42.10789
NC_000069.7	31149263	Skil	6.27E-12	21.94876
NC_000069.7	31149269	Skil	6.81E-09	18.0281
NC_000069.7	31149283	Skil	5.95E-07	15.37402

NC_000069.7	31149295	Skil	1.63E-08	16.93747
NC_000069.7	31149314	Skil	2.14E-08	15.96136
NC_000069.7	31149323	Skil	6.07E-10	21.21635
NC_000069.7	51469240	5031434O11Rik	4.56E-15	35.18768
NC_000069.7	68500711	Iqschfp	7.57E-11	16.01869
NC_000069.7	68500791	Iqschfp	2.8E-09	15.88344
NC_000069.7	82938392	Fga	2.27E-07	10.21765
NC_000069.7	88996826	Rusc1	3.64E-08	10.3585
NC_000069.7	89134908	Mir92b	3.35E-08	42.98514
NC_000069.7	89135052	Mir92b	2.8E-07	40.02247
NC_000069.7	89836327	null	3.98E-08	-25.3285
NC_000069.7	94590831	null	2.31E-15	23.19047
NC_000069.7	1.09E+08	Fndc7	1.06E-08	26.33752
NC_000069.7	1.22E+08	null	5.16E-07	28.25648
NC_000069.7	1.22E+08	null	2.97E-10	31.27685
NC_000069.7	1.22E+08	null	1.22E-08	34.80616
NC_000069.7	1.26E+08	Arsj	2.89E-11	-16.1948
NC_000069.7	1.3E+08	Lrit3	5.19E-08	11.13131
NC_000069.7	1.31E+08	Lef1	8.9E-09	-25.2049
NC_000069.7	1.31E+08	Lef1	1.03E-08	-26.715
NC_000069.7	1.31E+08	Lef1	5.66E-11	-26.9055
NC_000069.7	1.42E+08	Gbp3	1.08E-08	12.94857
NC_000069.7	1.42E+08	Gbp3	5.45E-12	11.93556
NC_000069.7	1.45E+08	Ccn1	8.85E-07	17.88793
NC_000069.7	1.46E+08	Syde2	9.58E-09	17.93992
NC_000069.7	1.46E+08	Syde2	3.12E-11	18.63882
NC_000070.7	41507631	1110017D15Rik	7.78E-08	21.45217
NC_000070.7	41507765	1110017D15Rik	2.04E-07	15.20624
NC_000070.7	46190878	Xpa	2.13E-07	-17.0072
NC_000070.7	1.17E+08	Rnf220	2.55E-07	19.60585
NC_000070.7	1.17E+08	Rnf220	9.35E-08	21.192
NC_000070.7	1.31E+08	Gm46723	7.67E-07	-24.0562
NC_000070.7	1.33E+08	Gpatch3	8.34E-13	19.9459
NC_000070.7	1.37E+08	Gm31820	5.18E-08	11.77992
NC_000070.7	1.39E+08	Rnf186	1.23E-14	33.31048
NC_000070.7	1.39E+08	Rnf186	2.86E-15	36.9524
NC_000070.7	1.39E+08	Rnf186	1.41E-13	33.48176
NC_000070.7	1.39E+08	Rnf186	1.17E-07	26.45457
NC_000070.7	1.39E+08	Rnf186	1.1E-10	28.43715
NC_000070.7	1.39E+08	Rnf186	6.57E-10	28.81271
NC_000070.7	1.4E+08	Klhdc7a	1.71E-08	29.9833
NC_000070.7	1.42E+08	Efhd2	4.76E-07	39.74313
NC_000070.7	1.42E+08	Efhd2	8.82E-09	38.92749
NC_000070.7	1.46E+08	null	4.48E-07	15.00033
NC_000070.7	1.46E+08	null	5.49E-09	16.06319
NC_000070.7	1.46E+08	null	1.5E-08	15.56227
NC_000070.7	1.46E+08	null	1.18E-08	17.43951

NC_000070.7	1.46E+08	null	3.93E-08	15.42822
NC_000070.7	1.46E+08	null	2.03E-07	15.67148
NC_000070.7	1.46E+08	null	6.11E-08	14.78843
NC_000070.7	1.46E+08	null	5.46E-12	20.04256
NC_000070.7	1.46E+08	null	7.13E-10	18.36998
NC_000070.7	1.46E+08	null	5.06E-07	23.3553
NC_000070.7	1.46E+08	null	9.15E-07	23.08683
NC_000070.7	1.46E+08	null	4.01E-07	24.2557
NC_000070.7	1.46E+08	null	9.3E-08	24.82698
NC_000070.7	1.46E+08	null	3.45E-13	24.95801
NC_000070.7	1.46E+08	null	2.35E-08	20.92208
NC_000070.7	1.46E+08	null	1.23E-11	22.90917
NC_000070.7	1.49E+08	Casz1	8.02E-09	36.47668
NC_000070.7	1.49E+08	Casz1	7.19E-07	28.70335
NC_000070.7	1.52E+08	Espn	9.82E-11	37.46257
NC_000071.7	15061732	null	8.16E-10	38.51033
NC_000071.7	15111529	null	9.46E-09	-14
NC_000071.7	38008986	Gm33993	8.66E-08	-20.2506
NC_000071.7	38059284	null	2.15E-07	13.81304
NC_000071.7	38059340	null	2.57E-07	15.9859
NC_000071.7	40952587	null	6.95E-07	18.63276
NC_000071.7	44826121	Ldb2	8.84E-09	-24.8732
NC_000071.7	53401377	Gm30519	9.19E-07	27.15034
NC_000071.7	54447845	null	1.51E-09	11.08886
NC_000071.7	72334436	null	1.87E-12	16.98262
NC_000071.7	72334445	null	7.67E-09	10.32138
NC_000071.7	72334464	null	4.07E-12	11.85644
NC_000071.7	80048716	null	2.35E-13	14.12947
NC_000071.7	1E+08	Hnrnpdl	1.66E-07	17.80266
NC_000071.7	1E+08	Hnrnpdl	3.8E-07	16.35483
NC_000071.7	1E+08	Hnrnpdl	7.04E-07	12.11195
NC_000071.7	1E+08	Hnrnpdl	5.26E-07	23.31368
NC_000071.7	1.15E+08	Fam222a	3.09E-10	18.1665
NC_000071.7	1.21E+08	Dtx1	1.08E-08	19.55506
NC_000071.7	1.22E+08	null	5.1E-10	13.9211
NC_000071.7	1.22E+08	null	2.12E-08	19.80558
NC_000071.7	1.23E+08	null	4.81E-09	27.82293
NC_000071.7	1.23E+08	Rhof	2.03E-10	20.02462
NC_000071.7	1.23E+08	Rhof	6.49E-08	15.97427
NC_000071.7	1.23E+08	Bcl7a	1.56E-11	22.9943
NC_000071.7	1.24E+08	Arl6ip4	7.1E-09	12.65084
NC_000071.7	1.25E+08	Ncor2	3.92E-08	13.59377
NC_000071.7	1.25E+08	Ncor2	9.55E-17	21.27526
NC_000071.7	1.25E+08	n-TAtgc10	6.78E-07	15.57659
NC_000071.7	1.38E+08	Mospd3	2.62E-07	10.50639
NC_000071.7	1.38E+08	Mblac1	8.5E-08	32.93271
NC_000071.7	1.46E+08	null	1.01E-09	-10.8149

NC_000071.7	1.5E+08	Fry	1.77E-12	-23.5988
NC_000072.7	3201396	null	5.28E-07	12.56568
NC_000072.7	13070166	Tmem106b	8.66E-08	14.61092
NC_000072.7	28420954	Gcc1	2.18E-07	16.9034
NC_000072.7	28421007	Gcc1	5.18E-10	15.46789
NC_000072.7	29485068	Kcp	8.39E-08	35.12896
NC_000072.7	52231022	null	1.34E-10	10.53282
NC_000072.7	52231031	null	2.05E-07	10.21683
NC_000072.7	57512040	Ppm1k	1.29E-07	15.03788
NC_000072.7	57512053	Ppm1k	6.52E-08	16.10931
NC_000072.7	72755962	Tcf7l1	4.93E-08	16.63025
NC_000072.7	83054641	Pcgf1	1.14E-07	16.14134
NC_000072.7	90683671	Iqsec1	9.41E-09	25.63503
NC_000072.7	95422778	null	2.27E-07	-10.1732
NC_000072.7	1.08E+08	Itpr1	4.83E-10	20.52143
NC_000072.7	1.13E+08	Prrt3	5.42E-08	28.26105
NC_000072.7	1.15E+08	Syn2	4.16E-08	22.88148
NC_000072.7	1.26E+08	Vwf	6.16E-07	21.27625
NC_000072.7	1.26E+08	Ano2	6.79E-09	-14.0619
NC_000072.7	1.26E+08	Ntf3	4.74E-07	27.33565
NC_000072.7	1.27E+08	Kcna5	2.28E-07	19.33569
NC_000072.7	1.34E+08	null	2.51E-07	18.36069
NC_000072.7	1.35E+08	1700055D16Rik	8E-07	13.46033
NC_000072.7	1.35E+08	1700055D16Rik	1.71E-12	16.12394
NC_000072.7	1.44E+08	null	3.63E-09	-17.481
NC_000072.7	1.45E+08	Lmntd1	1.61E-07	34.30674
NC_000072.7	1.46E+08	null	6.31E-11	13.56511
NC_000072.7	1.46E+08	null	1.04E-09	12.4529
NC_000072.7	1.46E+08	null	5.39E-07	10.62792
NC_000073.7	18748655	Foxa3	1.27E-10	23.91114
NC_000073.7	19097927	Ppp1r13l	1.83E-16	17.54329
NC_000073.7	28078715	Paf1	6.52E-07	11.27076
NC_000073.7	44601043	Tsks	1.99E-07	-13.761
NC_000073.7	44830952	Pth2	1.58E-07	17.79244
NC_000073.7	45164843	Tulp2	4.72E-07	22.14585
NC_000073.7	45164876	Tulp2	1.74E-07	24.80881
NC_000073.7	67956621	Fam169b	9.87E-07	17.25613
NC_000073.7	70000616	Nr2f2	9.09E-08	30.27627
NC_000073.7	79360494	Kif7	9.83E-11	53.66914
NC_000073.7	80495880	null	5.18E-07	30.75177
NC_000073.7	1.02E+08	Numa1	5.77E-08	11.54539
NC_000073.7	1.02E+08	Numa1	5.12E-08	14.15185
NC_000073.7	1.11E+08	null	3.98E-09	-18.3029
NC_000073.7	1.22E+08	Plk1	7.15E-13	38.00927
NC_000073.7	1.35E+08	Ptpre	1.88E-10	24.53049
NC_000073.7	1.41E+08	Sigirr	9.08E-09	25.31746
NC_000073.7	1.41E+08	Cracr2b	2.48E-08	14.38943

NC_000074.7	4287815	Lrrc8e	5.4E-09	18.94655
NC_000074.7	4288169	Map2k7	9.45E-08	17.02886
NC_000074.7	4289240	Map2k7	2.71E-11	15.22443
NC_000074.7	8711970	4921522P10Rik	1.02E-07	17.85264
NC_000074.7	10467048	Myo16	1.56E-07	-26.0621
NC_000074.7	12511705	Gm33326	1.69E-10	23.85897
NC_000074.7	12511713	Gm33326	1.83E-09	22.28307
NC_000074.7	18896000	Agpat5	1.21E-07	14.11437
NC_000074.7	18896002	Agpat5	4.14E-08	14.25581
NC_000074.7	23107326	Gm30135	4.2E-11	20.38655
NC_000074.7	27714383	Got1l1	2E-12	-17.6238
NC_000074.7	35272526	Dusp4	3.86E-08	13.89375
NC_000074.7	45522134	Mtnr1a	9.54E-07	12.97479
NC_000074.7	70355180	Ndufa13	2.33E-07	22.15857
NC_000074.7	70355222	Tssk6	5.41E-07	17.09511
NC_000074.7	70355243	Tssk6	6.3E-07	20.78569
NC_000074.7	70945963	Crlf1	9.42E-10	35.85825
NC_000074.7	71144492	Gm39191	9.7E-09	46.82759
NC_000074.7	71144954	Gm39191	2.84E-08	-19.2899
NC_000074.7	71206052	Rab3a	6.33E-07	19.32573
NC_000074.7	72145478	B3gnt3	1.13E-07	28.06152
NC_000074.7	72145508	B3gnt3	9.22E-08	27.42091
NC_000074.7	72145513	B3gnt3	5.72E-09	31.29971
NC_000074.7	72145523	B3gnt3	1.81E-10	39.13352
NC_000074.7	72145539	B3gnt3	8.29E-08	28.46374
NC_000074.7	91778125	Chd9	2.97E-08	11.63391
NC_000074.7	1.06E+08	LOC102636360	1.91E-07	17.0001
NC_000074.7	1.06E+08	Elmo3	2.24E-08	17.3302
NC_000074.7	1.06E+08	Carmil2	7.46E-09	22.83541
NC_000074.7	1.06E+08	Carmil2	2.29E-10	22.62417
NC_000074.7	1.07E+08	Slc7a6	5.26E-08	10.29127
NC_000074.7	1.18E+08	Plcg2	7.35E-07	-11.9562
NC_000074.7	1.19E+08	Cdh13	8.62E-07	-12.6482
NC_000074.7	1.2E+08	Adad2	9.43E-11	39.99126
NC_000074.7	1.22E+08	Fbxo31	1.89E-13	23.46546
NC_000074.7	1.22E+08	Fbxo31	1.43E-12	19.62886
NC_000074.7	1.22E+08	Fbxo31	1.53E-16	23.46041
NC_000074.7	1.22E+08	Fbxo31	4.86E-07	18.82467
NC_000074.7	1.22E+08	Fbxo31	4.19E-08	14.12632
NC_000074.7	1.23E+08	Cbfa2t3	1.69E-12	10.80532
NC_000075.7	22067519	Pigyl	8.53E-16	25.92046
NC_000075.7	31916887	Gm47465	1.18E-07	13.50099
NC_000075.7	43136057	Oaf	3.98E-07	22.82279
NC_000075.7	43136291	Oaf	7.76E-09	13.5969
NC_000075.7	43136340	Oaf	1.92E-07	13.59696
NC_000075.7	43456394	null	1.89E-07	-17.6545
NC_000075.7	44409772	Bcl9l	4.23E-11	16.23559

NC_000075.7	44409787	Bcl9l	7.03E-07	16.36866
NC_000075.7	44409795	Bcl9l	6.18E-08	19.75643
NC_000075.7	44409801	Bcl9l	7.93E-08	16.45996
NC_000075.7	44409822	Bcl9l	5.91E-12	18.52811
NC_000075.7	44409826	Bcl9l	2.69E-13	23.95917
NC_000075.7	44619943	Phldb1	3.02E-10	10.38894
NC_000075.7	44619948	Phldb1	2.71E-09	10.41613
NC_000075.7	44619983	Phldb1	1.24E-08	10.16155
NC_000075.7	45375589	Dscaml1	2E-09	20.81266
NC_000075.7	62251029	Anp32a	1.58E-07	22.21817
NC_000075.7	62251087	Anp32a	1.68E-09	24.75607
NC_000075.7	66956498	Tpm1	1.49E-10	13.35045
NC_000075.7	72848659	Pygo1	1.24E-08	-19.8129
NC_000075.7	78728903	null	3.51E-07	22.52754
NC_000075.7	78728922	null	3.01E-07	24.96751
NC_000075.7	78728963	null	1.99E-09	19.35028
NC_000075.7	78728996	null	2.26E-08	20.78778
NC_000075.7	85206806	Tent5a	3.14E-11	37.31632
NC_000075.7	1.06E+08	Iqcf3	8.65E-11	-26.7004
NC_000075.7	1.08E+08	Gnai2	3.13E-07	14.14165
NC_000075.7	1.11E+08	Pth1r	2.53E-09	10.09
NC_000075.7	1.11E+08	Tmie	3.22E-07	29.76986
NC_000075.7	1.11E+08	Epm2aip1	2.74E-08	12.29721
NC_000075.7	1.19E+08	Xylb	1.5E-07	11.7557
NC_000076.7	4571381	Esr1	7.15E-09	12.12877
NC_000076.7	4571587	Esr1	4.37E-08	21.19349
NC_000076.7	13843143	Hivep2	1.15E-07	18.40029
NC_000076.7	13843153	Hivep2	2.45E-08	25.03473
NC_000076.7	13843200	Hivep2	7.71E-08	21.41234
NC_000076.7	13843232	Hivep2	9.66E-10	24.91289
NC_000076.7	13843234	Hivep2	1.12E-11	29.70541
NC_000076.7	13843250	Hivep2	9.07E-08	20.34522
NC_000076.7	13843283	Hivep2	2.07E-08	20.39629
NC_000076.7	14062148	null	1.4E-08	14.28517
NC_000076.7	70356692	Fam13c	1.77E-07	26.38607
NC_000076.7	70803120	Bicc1	7.2E-10	-39.5078
NC_000076.7	80768064	Gadd45b	2.72E-12	10.12967
NC_000076.7	81012810	Eef2	7.41E-07	15.98786
NC_000076.7	81299717	Celf5	1.85E-07	-14.073
NC_000076.7	81309387	Celf5	1.23E-07	-12.8556
NC_000076.7	87115359	4930555G07Rik	1.16E-07	-17.3993
NC_000076.7	87350817	null	1.37E-14	21.28705
NC_000076.7	87350841	null	7.98E-08	16.15287
NC_000076.7	91017549	Tmpo	6.11E-09	21.61008
NC_000076.7	91017554	Tmpo	3.9E-08	20.84078
NC_000076.7	94298889	Tmcc3	1.16E-08	34.17626
NC_000076.7	94298903	Tmcc3	2.98E-07	29.97391

NC_000076.7	99039793	null	5.16E-09	22.78333
NC_000076.7	99039817	null	2.07E-09	23.85825
NC_000076.7	1.18E+08	Gm32605	1.06E-09	39.33109
NC_000076.7	1.27E+08	null	4.14E-07	23.30735
NC_000076.7	1.27E+08	Agap2	6.83E-09	22.24692
NC_000076.7	1.27E+08	Agap2	4.68E-08	21.05386
NC_000076.7	1.27E+08	Agap2	8.17E-08	33.07423
NC_000076.7	1.27E+08	Agap2	1.9E-08	33.08492
NC_000076.7	1.27E+08	Agap2	3.57E-07	31.05642
NC_000076.7	1.27E+08	Agap2	5.4E-09	33.66767
NC_000076.7	1.27E+08	Agap2	2.65E-09	34.36443
NC_000076.7	1.27E+08	Arhgap9	4.52E-08	13.91658
NC_000076.7	1.27E+08	Arhgap9	1.48E-08	17.47006
NC_000076.7	1.27E+08	Arhgap9	1.31E-10	16.80348
NC_000076.7	1.27E+08	Lrp1	4.05E-08	22.79542
NC_000076.7	1.29E+08	Pym1	5.17E-09	14.43351
NC_000077.7	5658348	Mrps24	9.55E-10	18.0229
NC_000077.7	5960290	Camk2b	2.78E-07	16.61292
NC_000077.7	8576760	Tns3	8.69E-10	16.56865
NC_000077.7	18964758	Meis1	9.48E-07	23.78225
NC_000077.7	22922581	Commd1	7.65E-10	21.09873
NC_000077.7	22922602	Commd1	6.52E-09	19.51915
NC_000077.7	22922609	Commd1	5.37E-08	18.33951
NC_000077.7	22922625	Commd1	6.45E-08	18.04309
NC_000077.7	22922635	Commd1	1.65E-08	19.0566
NC_000077.7	22922646	Commd1	8.49E-09	18.4607
NC_000077.7	22922654	Commd1	3.64E-08	18.57459
NC_000077.7	22922671	Commd1	3.43E-08	18.59759
NC_000077.7	22922684	Commd1	1.69E-09	21.41683
NC_000077.7	22922702	Commd1	4.22E-09	20.59928
NC_000077.7	22922721	Commd1	1.28E-07	17.76986
NC_000077.7	22922731	Commd1	7.91E-10	20.12436
NC_000077.7	43517620	null	7.75E-09	12.71794
NC_000077.7	63155944	null	4.52E-07	-35.1898
NC_000077.7	63813977	Hs3st3b1	2.48E-08	11.27781
NC_000077.7	67385786	Gas7	1.41E-07	-11.4915
NC_000077.7	68952141	Borcs6	4.83E-07	14.73688
NC_000077.7	68952162	Borcs6	1.58E-08	17.66788
NC_000077.7	70365978	null	3.26E-11	15.0927
NC_000077.7	79681546	9130204K15Rik	4.64E-07	23.24376
NC_000077.7	82453142	null	4.37E-07	-27.8062
NC_000077.7	98673840	Gm31862	3.82E-09	10.52365
NC_000077.7	98673850	Gm31862	1.01E-09	10.70158
NC_000077.7	1.02E+08	Etv4	4.81E-07	24.98459
NC_000077.7	1.02E+08	Hdac5	1.8E-08	21.49613
NC_000077.7	1.02E+08	Hdac5	2.27E-07	16.02188
NC_000077.7	1.09E+08	Gm36876	7.53E-10	13.73266

NC_000077.7	1.09E+08	Gm36876	2.07E-09	10.38706
NC_000077.7	1.09E+08	Gm36876	2.15E-09	10.08871
NC_000077.7	1.09E+08	Gm36876	1.91E-08	10.61476
NC_000077.7	1.09E+08	Gm36876	3.81E-10	11.14927
NC_000077.7	1.09E+08	Gm36876	1.23E-11	12.34079
NC_000077.7	1.09E+08	Gm36876	5.03E-10	10.1244
NC_000077.7	1.09E+08	Gm36876	4.89E-14	15.08043
NC_000077.7	1.1E+08	Map2k6	2.17E-14	17.7528
NC_000077.7	1.17E+08	Septin9	3.96E-07	10.41377
NC_000077.7	1.18E+08	Afmid	1.46E-07	-22.6328
NC_000077.7	1.18E+08	Afmid	2.83E-07	12.67277
NC_000077.7	1.18E+08	Dnah17	6.96E-08	-18.4191
NC_000077.7	1.19E+08	Gm53657	6.67E-07	10.14046
NC_000077.7	1.19E+08	Gm53657	5.88E-07	13.0151
NC_000077.7	1.19E+08	null	5.55E-10	15.46245
NC_000077.7	1.19E+08	null	3.88E-17	17.36386
NC_000077.7	1.19E+08	null	9.48E-07	15.4401
NC_000077.7	1.19E+08	null	1.38E-07	10.99288
NC_000077.7	1.19E+08	null	1.21E-13	17.96517
NC_000077.7	1.2E+08	Rptor	2.75E-07	-22.7563
NC_000077.7	1.2E+08	Aatk	4.05E-08	35.74449
NC_000077.7	1.2E+08	Gm9734	6.68E-11	14.73052
NC_000077.7	1.2E+08	Gm9734	2.31E-08	13.48839
NC_000077.7	1.2E+08	Gm9734	1.37E-08	12.74306
NC_000077.7	1.2E+08	0610009L18Rik	5.62E-07	15.7866
NC_000078.7	20895220	null	3.22E-07	15.01514
NC_000078.7	20970499	3110053B16Rik	1.84E-07	12.39516
NC_000078.7	20970538	3110053B16Rik	9.33E-10	13.14861
NC_000078.7	21833805	6030426L16Rik	3.28E-10	26.95905
NC_000078.7	60972249	null	5.46E-08	19.68169
NC_000078.7	80804271	Ccdc177	3.29E-18	34.14976
NC_000078.7	80804275	Ccdc177	8.95E-09	25.67739
NC_000078.7	82018125	Pcnx	3.34E-07	-46.21
NC_000078.7	85532638	null	1.85E-08	12.12172
NC_000078.7	87022368	null	2.41E-08	-20.4294
NC_000078.7	99611481	Gm53756	1.09E-07	20.58731
NC_000078.7	99611496	Gm53756	1.31E-08	17.19038
NC_000078.7	99611500	Gm53756	1.21E-10	20.27601
NC_000078.7	99611506	Gm53756	9.55E-10	17.42785
NC_000078.7	99611516	Gm53756	1.1E-07	14.62753
NC_000078.7	99611526	Gm53756	3.89E-10	18.52876
NC_000078.7	99611546	Gm53756	6.2E-11	22.40437
NC_000078.7	99611575	Gm53756	1.8E-07	16.53317
NC_000078.7	1.08E+08	Gm33236	2.84E-12	43.24641
NC_000078.7	1.11E+08	n-TIaat6	1.08E-09	30.27535
NC_000078.7	1.11E+08	n-TIaat6	2E-08	21.99878
NC_000078.7	1.11E+08	n-TIaat6	9.8E-08	20.05972

NC_000078.7	1.19E+08	null	2.49E-07	-14.5118
NC_000079.7	3528861	2810429I04Rik	2.36E-09	13.74306
NC_000079.7	16192195	Inhba	1.59E-07	39.94393
NC_000079.7	22165960	Pom121l2	4.92E-08	25.61847
NC_000079.7	30668515	null	3.36E-07	23.63817
NC_000079.7	37641531	null	2.23E-10	-30.8723
NC_000079.7	45023292	Jarid2	8.66E-42	10.1229
NC_000079.7	45862956	Atxn1	1.81E-10	12.39184
NC_000079.7	57018625	Trpc7	1.58E-12	-48.4868
NC_000079.7	57675249	Spock1	1.61E-07	10.19901
NC_000079.7	59969539	Tut7	7.19E-07	24.01321
NC_000079.7	64302049	Zfp367	1.19E-07	11.8142
NC_000079.7	95753096	F2r	7.7E-08	18.84452
NC_000079.7	1.02E+08	Pik3r1	1.24E-07	13.31868
NC_000079.7	1.12E+08	null	3.81E-07	-24.113
NC_000079.7	1.13E+08	null	3.42E-10	13.99968
NC_000079.7	1.13E+08	null	1.9E-14	15.61109
NC_000079.7	1.13E+08	null	4.43E-12	14.51638
NC_000079.7	1.17E+08	Emb	8.07E-08	27.01197
NC_000079.7	1.2E+08	Tmem267	4.61E-08	-11.3021
NC_000079.7	1.2E+08	Tmem267	9.32E-08	-10.0962
NC_000080.7	4237413	Nr1d2	4.35E-07	10.54053
NC_000080.7	20753039	6230400D17Rik	8.07E-08	22.24724
NC_000080.7	25760639	Zcchc24	1.26E-07	36.06118
NC_000080.7	45682808	Gm34250	4.85E-08	-11.1031
NC_000080.7	48392646	Peli2	3.68E-07	13.89417
NC_000080.7	57636444	Cryl1	1.7E-07	10.57169
NC_000080.7	66348876	Ephx2	1.1E-08	22.55801
NC_000080.7	70745992	Bmp1	1.02E-08	19.30013
NC_000080.7	70746038	Bmp1	1.54E-09	20.65408
NC_000080.7	70746102	Bmp1	8.87E-11	21.07601
NC_000080.7	72205020	null	2.95E-07	23.02612
NC_000080.7	93453464	Pcdh9	1.73E-08	20.15067
NC_000080.7	1.03E+08	Gm41236	1.57E-08	17.61797
NC_000080.7	1.04E+08	Gm35197	1.49E-07	26.5203
NC_000080.7	1.04E+08	Gm35197	2.55E-08	22.16952
NC_000080.7	1.04E+08	Gm35197	6.49E-08	20.76732
NC_000080.7	1.04E+08	Gm35197	5.13E-09	21.78421
NC_000080.7	1.04E+08	Gm35197	1.35E-08	21.90722
NC_000080.7	1.12E+08	Slitrk5	5.07E-07	26.96038
NC_000081.7	3053519	null	1.55E-08	14.90895
NC_000081.7	12502322	Pdzd2	8.37E-07	14.388
NC_000081.7	73055321	37469	6.89E-07	36.46436
NC_000081.7	75869847	Mapk15	7.23E-08	44.99769
NC_000081.7	75869889	Mapk15	4.41E-07	46.7389
NC_000081.7	75869935	Mapk15	6.46E-10	51.98589
NC_000081.7	75869944	Mapk15	1.45E-07	44.41953

NC_000081.7	76883630	Gm53916	6.09E-10	18.8219
NC_000081.7	77741901	Gm53901	3.8E-07	26.41225
NC_000081.7	77741903	Gm53901	1.06E-07	28.68949
NC_000081.7	78289685	Tst	1.78E-08	14.87615
NC_000081.7	78289737	Tst	3.42E-11	20.55832
NC_000081.7	78352958	Tmprss6	1.4E-08	13.40768
NC_000081.7	78851925	Triobp	3.46E-11	17.67132
NC_000081.7	78851957	Triobp	1.03E-12	19.08281
NC_000081.7	78851989	Triobp	4.26E-12	16.71306
NC_000081.7	81743870	Tob2	2.12E-08	21.66938
NC_000081.7	81743880	Tob2	9.26E-08	17.31835
NC_000081.7	81743932	Tob2	6.87E-07	14.52921
NC_000081.7	89383493	Shank3	3.29E-08	14.43827
NC_000081.7	96506559	Slc38a1	5E-07	21.02394
NC_000081.7	97271738	Pced1b	7.13E-07	15.02171
NC_000081.7	97274768	Pced1b	2.66E-10	12.04067
NC_000081.7	97274781	Pced1b	1.58E-07	10.11026
NC_000081.7	97274804	Pced1b	3.94E-07	12.02159
NC_000081.7	97899918	Col2a1	2.6E-07	17.44514
NC_000081.7	97899979	Col2a1	3.06E-07	14.39647
NC_000081.7	1.01E+08	Cela1	1.22E-10	27.53491
NC_000081.7	1.01E+08	Cela1	6.5E-08	22.01986
NC_000081.7	1.01E+08	Fignl2	8.27E-07	25.47039
NC_000081.7	1.01E+08	Fignl2	3.09E-11	31.92133
NC_000081.7	1.01E+08	Fignl2	1.12E-14	34.84635
NC_000081.7	1.01E+08	Fignl2	7.47E-08	10.60027
NC_000081.7	1.01E+08	Fignl2	8.97E-07	20.19093
NC_000081.7	1.02E+08	Krt79	7.3E-10	21.93574
NC_000081.7	1.02E+08	Krt79	6.01E-08	24.54478
NC_000081.7	1.02E+08	Krt79	1.06E-10	47.35159
NC_000081.7	1.02E+08	null	2.43E-08	12.00603
NC_000081.7	1.02E+08	null	1.27E-08	11.01454
NC_000082.7	28659380	Mb21d2	3.16E-08	-15.4866
NC_000082.7	31983663	Nrros	2.68E-11	-37.6452
NC_000082.7	31983677	Nrros	2.98E-14	-42.7345
NC_000082.7	31983695	Nrros	9.75E-15	-40.8508
NC_000082.7	31983750	Nrros	1.61E-08	-32.7782
NC_000082.7	31983763	Nrros	8.86E-10	-35.2011
NC_000082.7	31983783	Nrros	4.31E-10	-34.3625
NC_000082.7	33571874	Heg1	1.26E-07	31.37947
NC_000082.7	55771904	null	1.37E-08	16.75623
NC_000082.7	57211639	Cmss1	0	13.68827
NC_000082.7	57211651	Cmss1	0	12.39565
NC_000082.7	57211655	Cmss1	8.07E-41	11.14773
NC_000082.7	57211658	Cmss1	0	11.82889
NC_000082.7	57211669	Cmss1	0	12.32715
NC_000082.7	57211675	Cmss1	4.12E-43	10.98134

NC_000082.7	57211699	Cmss1	2.38E-44	10.89766
NC_000082.7	57211725	Cmss1	0	13.19431
NC_000082.7	57211737	Cmss1	0	13.54274
NC_000082.7	57211754	Cmss1	0	11.84489
NC_000082.7	57211758	Cmss1	0	11.45198
NC_000082.7	57211762	Cmss1	0	11.06172
NC_000082.7	57211771	Cmss1	0	11.44793
NC_000082.7	57211780	Cmss1	0	11.54865
NC_000082.7	57211785	Cmss1	1.06E-40	11.05474
NC_000082.7	57211814	Cmss1	0	13.236
NC_000082.7	57211815	Cmss1	0	10.17582
NC_000082.7	57211844	Cmss1	3.78E-43	11.2935
NC_000082.7	57211854	Cmss1	0	13.62612
NC_000082.7	57211856	Cmss1	0	12.64389
NC_000082.7	57211860	Cmss1	0	14.9096
NC_000082.7	57211864	Cmss1	0	12.89553
NC_000082.7	57211960	Cmss1	1.63E-11	24.84527
NC_000082.7	57211981	Cmss1	1.41E-07	20.83735
NC_000082.7	57211986	Cmss1	2.51E-16	27.95308
NC_000082.7	57211989	Cmss1	4.35E-12	25.32133
NC_000082.7	57211996	Cmss1	6.92E-17	29.65882
NC_000082.7	57212000	Cmss1	9.62E-08	20.99916
NC_000082.7	57212015	Cmss1	2.18E-08	20.07812
NC_000082.7	57212019	Cmss1	1.14E-12	26.33112
NC_000082.7	76477065	null	1.35E-07	22.96964
NC_000082.7	92295134	Clic6	9.52E-10	-36.331
NC_000082.7	92402824	Runx1	1.57E-07	33.95063
NC_000082.7	92402834	Runx1	2.83E-07	31.36524
NC_000082.7	92402943	Runx1	5.7E-07	25.85581
NC_000082.7	92402945	Runx1	4.81E-07	25.77639
NC_000083.7	10554919	null	1.3E-07	11.106
NC_000083.7	10554941	null	4.83E-07	19.80522
NC_000083.7	24425883	Ntn3	5.87E-09	63.43847
NC_000083.7	24427765	Ntn3	3.73E-09	22.87101
NC_000083.7	24877401	Npw	1.6E-07	43.7141
NC_000083.7	24877402	Npw	6.4E-07	27.51941
NC_000083.7	25087045	Gm41549	8.37E-07	17.55379
NC_000083.7	29082153	Pnpla1	4.39E-09	15.66445
NC_000083.7	34338985	Brd2	1.88E-11	17.8615
NC_000083.7	34338990	Brd2	6.32E-09	14.27919
NC_000083.7	34339007	Brd2	2.02E-11	17.2012
NC_000083.7	34339016	Brd2	2E-07	12.16624
NC_000083.7	34339046	Brd2	9.19E-07	11.33342
NC_000083.7	35114489	Zbtb12	5.25E-11	33.06126
NC_000083.7	40153917	Gm26917	2.63E-23	10.36033
NC_000083.7	40154052	Gm26917	1.14E-08	43.28352
NC_000083.7	40154190	Gm26917	3.28E-17	10.89337

NC_000083.7	40154203	Gm26917	2.78E-29	15.93276
NC_000083.7	40154356	Gm26917	3.64E-20	12.78849
NC_000083.7	40154376	Gm26917	1E-18	12.56449
NC_000083.7	40154395	Gm26917	5.18E-27	15.35377
NC_000083.7	40154409	Gm26917	7.1E-23	17.72805
NC_000083.7	40154443	Gm26917	1.09E-29	11.21658
NC_000083.7	40154446	Gm26917	2.29E-25	10.05956
NC_000083.7	40154454	Gm26917	1.07E-29	11.45121
NC_000083.7	40154463	Gm26917	8.93E-33	13.23079
NC_000083.7	40154464	Gm26917	7.26E-27	10.23172
NC_000083.7	40154465	Gm26917	2.13E-28	12.0033
NC_000083.7	40154483	Gm26917	3.53E-32	12.50511
NC_000083.7	40154490	Gm26917	2.57E-24	11.52115
NC_000083.7	40154491	Gm26917	4.7E-25	10.9741
NC_000083.7	40154509	Gm26917	8.65E-35	12.35993
NC_000083.7	40154513	Gm26917	2.34E-34	10.79038
NC_000083.7	40154515	Gm26917	3.31E-36	11.57068
NC_000083.7	40154537	Gm26917	6.89E-42	13.13472
NC_000083.7	40154557	Gm26917	2.02E-34	11.28899
NC_000083.7	40154585	Gm26917	3.11E-34	11.80244
NC_000083.7	40154740	Gm26917	5.7E-10	12.53029
NC_000083.7	40154807	Gm26917	9.95E-44	12.77727
NC_000083.7	40154818	Gm26917	0	12.84335
NC_000083.7	40154819	Gm26917	0	10.93431
NC_000083.7	40154829	Gm26917	0	12.55034
NC_000083.7	40154830	Gm26917	0	12.33238
NC_000083.7	40154849	Gm26917	0	11.66599
NC_000083.7	40154850	Gm26917	0	12.24512
NC_000083.7	40154862	Gm26917	4.83E-28	13.76727
NC_000083.7	40154870	Gm26917	0	10.64627
NC_000083.7	40154871	Gm26917	0	10.50908
NC_000083.7	40154882	Gm26917	0	10.14617
NC_000083.7	40154889	Gm26917	0	12.21354
NC_000083.7	40154893	Gm26917	0	11.98881
NC_000083.7	40154894	Gm26917	5.65E-39	10.43651
NC_000083.7	40154895	Gm26917	0	11.24068
NC_000083.7	40154900	Gm26917	0	10.83531
NC_000083.7	40154912	Gm26917	0	11.77058
NC_000083.7	40154921	Gm26917	0	12.01052
NC_000083.7	40154927	Gm26917	0	11.86565
NC_000083.7	40154935	Gm26917	0	12.14116
NC_000083.7	40154936	Gm26917	0	11.07368
NC_000083.7	40154941	Gm26917	0	11.0855
NC_000083.7	40154946	Gm26917	0	11.10614
NC_000083.7	40154947	Gm26917	0	11.53379
NC_000083.7	40154959	Gm26917	0	10.41568
NC_000083.7	40154961	Gm26917	0	11.56668

NC_000083.7	40154962	Gm26917	0	10.94148
NC_000083.7	40154980	Gm26917	2.8E-45	11.05539
NC_000083.7	40154981	Gm26917	0	10.97136
NC_000083.7	40154988	Gm26917	3.65E-39	10.55641
NC_000083.7	40154989	Gm26917	0	11.38447
NC_000083.7	40154997	Gm26917	0	11.91134
NC_000083.7	40155012	Gm26917	2.42E-32	11.98515
NC_000083.7	40155013	Gm26917	0	13.91789
NC_000083.7	40155057	Gm26917	4.2E-45	10.19089
NC_000083.7	40155059	Gm26917	1.63E-43	10.26388
NC_000083.7	40155063	Gm26917	9.11E-41	10.21112
NC_000083.7	40155064	Gm26917	1.36E-31	10.11969
NC_000083.7	40155065	Gm26917	0	11.34857
NC_000083.7	40155090	Gm26917	1E-32	10.40591
NC_000083.7	40155093	Gm26917	6.62E-25	10.24279
NC_000083.7	40155103	Gm26917	1.54E-44	12.13359
NC_000083.7	40155131	Gm26917	5.29E-08	12.40738
NC_000083.7	40155132	Gm26917	3.78E-41	10.16392
NC_000083.7	40155171	Gm26917	1.19E-13	11.17464
NC_000083.7	40155174	Gm26917	1.04E-13	10.03728
NC_000083.7	40155189	Gm26917	1.34E-14	11.14814
NC_000083.7	40155193	Gm26917	1.43E-12	10.11654
NC_000083.7	40155280	Gm26917	2.13E-12	10.29188
NC_000083.7	40155298	Gm26917	3.39E-15	11.10193
NC_000083.7	40155300	Gm26917	1.06E-17	11.41716
NC_000083.7	40155306	Gm26917	1.67E-17	10.59447
NC_000083.7	40155321	Gm26917	3.6E-20	12.13267
NC_000083.7	40155327	Gm26917	5.43E-18	10.98706
NC_000083.7	40155433	Gm26917	2.8E-23	10.1521
NC_000083.7	40155440	Gm26917	0	10.44058
NC_000083.7	40155443	Gm26917	0	13.2341
NC_000083.7	40155445	Gm26917	0	11.81439
NC_000083.7	40155448	Gm26917	0	12.48474
NC_000083.7	40155451	Gm26917	0	10.52862
NC_000083.7	40155455	Gm26917	0	12.17676
NC_000083.7	40155471	Gm26917	0	11.20711
NC_000083.7	40155475	Gm26917	0	10.52994
NC_000083.7	40155488	Gm26917	0	13.88589
NC_000083.7	40155498	Gm26917	1.4E-45	11.02858
NC_000083.7	40155506	Gm26917	0	12.06456
NC_000083.7	40155512	Gm26917	3.42E-43	11.03226
NC_000083.7	40155524	Gm26917	0	10.58645
NC_000083.7	40155529	Gm26917	0	12.72612
NC_000083.7	40155535	Gm26917	0	12.1212
NC_000083.7	40155543	Gm26917	3.18E-43	10.03386
NC_000083.7	40155551	Gm26917	0	11.67994
NC_000083.7	40155559	Gm26917	9.48E-07	11.68091

NC_000083.7	40155569	Gm26917	1.91E-11	18.68489
NC_000083.7	40155574	Gm26917	1.91E-10	19.00189
NC_000083.7	40155579	Gm26917	2.04E-11	19.36765
NC_000083.7	40155582	Gm26917	7.3E-11	15.69766
NC_000083.7	40155634	Gm26917	5.92E-11	38.45966
NC_000083.7	40155751	Gm26917	1.96E-09	17.66234
NC_000083.7	40155764	Gm26917	3.65E-08	17.60582
NC_000083.7	40155791	Gm26917	9.04E-10	19.16143
NC_000083.7	40155833	Gm26917	4.44E-07	15.86221
NC_000083.7	40155976	Gm26917	0	13.66293
NC_000083.7	40155978	Gm26917	0	12.95408
NC_000083.7	40155982	Gm26917	0	12.51888
NC_000083.7	40155987	Gm26917	0	14.00788
NC_000083.7	40155997	Gm26917	0	13.07119
NC_000083.7	40155999	Gm26917	0	12.42362
NC_000083.7	40156017	Gm26917	0	12.53011
NC_000083.7	40156021	Gm26917	0	14.15994
NC_000083.7	40156040	Gm26917	0	12.87801
NC_000083.7	40156042	Gm26917	0	12.0428
NC_000083.7	40156081	Gm26917	0	13.26186
NC_000083.7	40156084	Gm26917	0	14.32053
NC_000083.7	40156104	Gm26917	0	12.95252
NC_000083.7	40156124	Gm26917	0	13.46372
NC_000083.7	40156128	Gm26917	0	15.05886
NC_000083.7	40156169	Gm26917	1.27E-08	30.26045
NC_000083.7	40156241	Gm26917	1.21E-07	11.3885
NC_000083.7	40156245	Gm26917	1.32E-11	13.27693
NC_000083.7	40156287	Gm26917	2.9E-13	11.02093
NC_000083.7	40156322	Gm26917	6.24E-18	13.64431
NC_000083.7	40156324	Gm26917	1.91E-12	10.24032
NC_000083.7	40156336	Gm26917	1.31E-12	11.03488
NC_000083.7	40156343	Gm26917	1.57E-17	12.98286
NC_000083.7	40156383	Gm26917	2.26E-14	10.50456
NC_000083.7	40156386	Gm26917	4.11E-18	11.28024
NC_000083.7	40156407	Gm26917	3.56E-19	12.10975
NC_000083.7	40156420	Gm26917	1.03E-11	12.73538
NC_000083.7	40156635	Gm26917	3.14E-08	12.11571
NC_000083.7	40156646	Gm26917	5.53E-08	10.70069
NC_000083.7	40156674	Gm26917	6.54E-13	14.18391
NC_000083.7	40156676	Gm26917	6.76E-10	11.67299
NC_000083.7	40156685	Gm26917	1.2E-10	12.69175
NC_000083.7	40156691	Gm26917	4.95E-10	14.22219
NC_000083.7	40156698	Gm26917	8.9E-13	14.79949
NC_000083.7	40156709	Gm26917	1.29E-12	12.79122
NC_000083.7	40156711	Gm26917	1.68E-07	11.32838
NC_000083.7	40156721	Gm26917	2.28E-07	11.85476
NC_000083.7	40156731	Gm26917	3.22E-11	16.23473

NC_000083.7	40156760	Gm26917	8.79E-10	11.88724
NC_000083.7	40157069	Gm26917	7.41E-08	22.98812
NC_000083.7	40157140	Gm26917	1.71E-28	13.7886
NC_000083.7	40157155	Gm26917	4.4E-25	10.42159
NC_000083.7	40157165	Gm26917	2.31E-30	13.3637
NC_000083.7	40157168	Gm26917	3.8E-26	11.93859
NC_000083.7	40157171	Gm26917	1.16E-33	13.19622
NC_000083.7	40157186	Gm26917	1.36E-31	12.17719
NC_000083.7	40157188	Gm26917	1.72E-27	10.58391
NC_000083.7	40157221	Gm26917	4.2E-45	11.79661
NC_000083.7	40157230	Gm26917	4.08E-31	10.5671
NC_000083.7	40157234	Gm26917	8.76E-39	10.62636
NC_000083.7	40157308	Gm26917	2.02E-30	11.99434
NC_000083.7	40157312	Gm26917	1.96E-44	13.90689
NC_000083.7	40157632	Gm26917	0	10.07286
NC_000083.7	40157681	Gm26917	0	10.39242
NC_000083.7	40158192	Gm26917	2.74E-07	20.16845
NC_000083.7	40158224	Gm26917	1.5E-10	34.06611
NC_000083.7	40158227	Gm26917	2.59E-10	33.56322
NC_000083.7	40158234	Gm26917	1.56E-10	33.99728
NC_000083.7	40158244	Gm26917	7.12E-11	30.79171
NC_000083.7	40158282	Gm26917	6.37E-08	22.83202
NC_000083.7	40158289	Gm26917	2.81E-08	30.02462
NC_000083.7	40158297	Gm26917	9.07E-08	27.13962
NC_000083.7	40158303	Gm26917	1.8E-07	17.19352
NC_000083.7	40158314	Gm26917	7.67E-09	29.22704
NC_000083.7	40158335	Gm26917	7.93E-14	36.10092
NC_000083.7	40158341	Gm26917	5.14E-07	19.53957
NC_000083.7	40158353	Gm26917	8.51E-12	34.62906
NC_000083.7	40158356	Gm26917	4.98E-11	29.65312
NC_000083.7	40158359	Gm26917	1.93E-11	32.68317
NC_000083.7	40158375	Gm26917	2.15E-09	27.51297
NC_000083.7	40158379	Gm26917	0	11.36298
NC_000083.7	40158518	Gm26917	6.93E-10	19.87431
NC_000083.7	40159119	Gm26917	2.24E-44	10.3928
NC_000083.7	40159135	Gm26917	2.33E-38	10.07903
NC_000083.7	40159152	Gm26917	1.33E-41	11.04574
NC_000083.7	40159155	Gm26917	0	12.12281
NC_000083.7	40159172	Gm26917	3.95E-24	19.58559
NC_000083.7	40159179	Gm26917	2.93E-20	14.73211
NC_000083.7	40159182	Gm26917	5.12E-20	15.16429
NC_000083.7	40159186	Gm26917	1.01E-21	14.22151
NC_000083.7	40159188	Gm26917	2.99E-19	13.2837
NC_000083.7	40159197	Gm26917	3.25E-22	15.21783
NC_000083.7	40159238	Gm26917	1.08E-16	13.58324
NC_000083.7	40159249	Gm26917	4.77E-16	15.87288
NC_000083.7	40159259	Gm26917	1.77E-11	19.75862

NC_000083.7	43347359	Tnfrsf21	2.16E-08	23.13488
NC_000083.7	43702704	Adgrf5	8.07E-07	14.61328
NC_000083.7	45930408	Gm52283	1.8E-07	11.10934
NC_000083.7	45930440	Gm52283	5.18E-07	10.18422
NC_000083.7	45930487	Gm52283	2.16E-09	14.52168
NC_000083.7	46863931	Srf	2.39E-11	23.0528
NC_000083.7	48078661	Tfeb	8.13E-09	13.03765
NC_000083.7	48954703	null	7.25E-10	33.89947
NC_000083.7	50995445	Plcl2	4.7E-11	15.72986
NC_000083.7	79328572	Prkd3	7.32E-08	37.52243
NC_000083.7	83584952	null	1.92E-07	23.8149
NC_000083.7	83584954	null	4.36E-08	22.35801
NC_000083.7	85096410	Lrpprc	3.34E-07	15.71783
NC_000084.7	20878447	B4galt6	5.95E-10	14.42236
NC_000084.7	37093785	Pcdha1	9.68E-07	37.92911
NC_000084.7	37881078	Gm37013	1.23E-08	24.40316
NC_000084.7	37881085	Gm37013	2.22E-09	19.86718
NC_000084.7	37889654	Gm37013	2.39E-08	49.65815
NC_000084.7	39126539	Arhgap26	3.88E-07	14.43304
NC_000084.7	39173250	Arhgap26	5.99E-09	30.96224
NC_000084.7	54586528	Redrum	5.37E-10	19.06866
NC_000084.7	62944011	null	2.5E-08	23.71893
NC_000084.7	75508991	Smad7	1.51E-09	15.87397
NC_000084.7	75509049	Smad7	1.98E-08	14.60775
NC_000084.7	77126031	Katnal2	8.85E-07	-24.5193
NC_000084.7	77138581	null	6.64E-10	15.8849
NC_000084.7	77273779	St8sia5	1.61E-07	30.74748
NC_000085.7	4561233	Pcx	4.45E-10	13.99872
NC_000085.7	6904500	Kcnk4	1E-13	21.93718
NC_000085.7	6904521	Kcnk4	6.19E-10	17.58206
NC_000085.7	13949746	null	5.09E-08	-17.5758
NC_000085.7	28312376	Glis3	7.83E-08	-18.8673
NC_000085.7	28312430	Glis3	2.44E-09	-19.2079
NC_000085.7	37684585	Gm32342	2.27E-08	18.25121
NC_000085.7	38111377	Rbp4	1.25E-07	14.4518
NC_000085.7	38111387	Rbp4	2.03E-07	14.1234
NC_000085.7	38111389	Rbp4	3.67E-07	13.3243
NC_000085.7	38111455	Rbp4	1.81E-09	15.87593
NC_000085.7	38111456	Rbp4	2.98E-08	12.84607
NC_000085.7	38111466	Rbp4	7.63E-08	12.50261
NC_000085.7	41827452	null	2.7E-08	19.81751
NC_000085.7	43909298	Dnmbp	5.33E-09	35.78703
NC_000085.7	43909322	Dnmbp	2.4E-07	48.29753
NC_000085.7	55049910	null	2.52E-07	-18.2696
NC_000086.8	98196404	null	2.21E-07	30.62049
NC_000086.8	1.03E+08	null	3.02E-07	12.28893
NC_000086.8	1.61E+08	Nhs	5.59E-10	50.18537

NC_000086.8	1.69E+08	Mid1	8.86E-08	12.94814
NC_000086.8	1.69E+08	Mid1	1.41E-27	14.68614
NC_000086.8	1.69E+08	Mid1	2.28E-12	11.19114
NC_000086.8	1.69E+08	Mid1	1.2E-24	19.36119
NC_000086.8	1.69E+08	Mid1	2.26E-16	15.87528
NC_000087.8	1286745	Ddx3y	2.32E-08	14.64035
NC_005089.1	3401	ND1	0	15.33953
NC_005089.1	3410	ND1	0	15.48192
NC_005089.1	3412	ND1	0	15.19452
NT_187064.1	65938	null	6.59E-08	-34.1374
NW_023337853.1	435	null	0	15.76717
NW_023337853.1	443	null	0	15.35764
NW_023337853.1	447	null	0	15.3014
NW_023337853.1	452	null	0	14.75721
NW_023337853.1	459	null	0	13.71952
NW_023337853.1	463	null	0	15.13454
NW_023337853.1	467	null	0	15.61462
NW_023337853.1	481	null	0	15.48509
NW_023337853.1	493	null	0	13.60863
NW_023337853.1	499	null	0	14.44726
NW_023337853.1	515	null	0	14.49195
NW_023337853.1	523	null	0	13.37707
NW_023337853.1	527	null	0	13.9825
NW_023337853.1	546	null	0	11.7052
NW_023337853.1	551	null	0	13.11021
NW_023337853.1	573	null	0	13.2418
NW_023337853.1	594	null	0	12.49519
NW_023337853.1	604	null	0	12.45114
NW_023337853.1	611	null	0	13.18695
NW_023337853.1	615	null	0	13.70029
NW_023337853.1	619	null	0	13.05707
NW_023337853.1	623	null	0	13.49136
NW_023337853.1	627	null	0	12.80471
NW_023337853.1	629	null	0	13.52736
NW_023337853.1	633	null	0	13.38118
NW_023337853.1	637	null	3.56E-17	10.33139
NW_023337853.1	638	null	0	13.2023
NW_023337853.1	641	null	0	13.86708
NW_023337853.1	645	null	0	14.15966
NW_023337853.1	649	null	0	10.94865
NW_023337853.1	654	null	0	10.9584
NW_023337853.1	660	null	0	11.09952
NW_023337853.1	664	null	0	10.38503
NW_023337853.1	669	null	0	10.37966
NW_023337853.1	676	null	0	11.12596
NW_023337853.1	678	null	0	10.80737
NW_023337853.1	680	null	0	11.71074

NW_023337853.1	688	null	0	10.42211
NW_023337853.1	692	null	0	10.25639
NW_023337853.1	696	null	0	10.62338
NW_023337853.1	829	null	1.45E-14	10.26289
NW_023337853.1	969	null	9.09E-13	15.20991
NW_023337853.1	985	null	1.82E-19	19.50347
NW_023337853.1	991	null	1.52E-07	11.17548
NW_023337853.1	996	null	3.18E-09	10.75133
NW_023337853.1	1000	null	1.55E-08	13.80545
NW_023337853.1	1003	null	1.77E-12	14.00505
NW_023337853.1	1017	null	1.45E-11	13.05958
NW_023337853.1	1028	null	4.92E-20	10.15961
NW_023337853.1	1037	null	1.72E-20	10.44086
NW_023337853.1	1082	null	2.08E-22	10.59531
NW_023337853.1	1086	null	0	10.70301
NW_023337853.1	1089	null	0	11.45097
NW_023337853.1	1092	null	6.03E-23	10.84571
NW_023337853.1	1097	null	2.56E-41	10.01911
NW_023337853.1	1111	null	0	11.14833
NW_023337853.1	1113	null	2.54E-41	10.25018
NW_023337853.1	1277	null	5.57E-07	13.89846
NW_023337853.1	1354	null	9.82E-07	13.34416
NW_023337853.1	1402	null	1.47E-19	14.00084
NW_023337853.1	1440	null	2.56E-16	10.59244
NW_023337853.1	1452	null	1.14E-17	12.33866
NW_023337853.1	1488	null	8.89E-22	13.17227
NW_023337853.1	1581	null	4.03E-16	11.78444
NW_023337853.1	1824	null	4.66E-10	10.39521
NW_023337853.1	28499	LOC118568479	8.8E-07	-19.278
NW_023337853.1	30390	null	2.2E-09	10.18509
NW_023337853.1	30820	null	0	10.56858
NW_023337853.1	30823	null	0	10.95476
NW_023337853.1	30825	null	0	12.36834
NW_023337853.1	30831	null	0	13.68797
NW_023337853.1	30858	null	0	12.42157
NW_023337853.1	30870	null	0	11.56421
NW_023337853.1	30874	null	0	11.21232
NW_023337853.1	30897	null	0	11.97271
NW_023337853.1	30903	null	0	12.19207
NW_023337853.1	30957	null	0	11.53092

Table S8. Gene Ontology (GO) analysis of the differentially methylated genes DMGs for CTR vs HFD, CTR vs HFD+beer, CTR vs CTR+beer and HFD vs HFD+beer comparisons. Gene ontology IDs (GO-ID), gene ontology terms (GO-term), associated genes found and corrected (Bonferroni step down) p-values as determined by ClueGO (http://apps.cytoscape.org/apps/cluego) are indicated.

**CTR vs HFD**

ID GO:	Associated Genes Found	Nr. Genes	Term	PValue
1508	[Ctnna3, Cxadr, Gm37013, Kcnip1, Kcnq1, Mtor, Scn8a]	7	action potential	0.0398
45778	[Acvr2b, Cyp27b1, Rxra, Tent5a, Wnt5a]	5	positive regulation of ossification	0.0225
50882	[Map1a, Mtor, Vti1a]	3	voluntary musculoskeletal movement	0.0059
60561	[Cyp27b1, Lef1, Lrp5, Pax2]	4	apoptotic process involved in morphogenesis	0.0202
61041	[Anxa5, Fgg, Hmgcr, Mtor, Phldb2, Pros1, Serpinf2]	7	regulation of wound healing	0.0167
61045	[Anxa5, Fgg, Hmgcr, Phldb2, Pros1, Serpinf2]	6	negative regulation of wound healing	0.0054
70423	[Irgm1, Nfkbia, Rela]	3	nucleotide-binding oligomerization domain containing signaling pathway	0.0479
70431	[Irgm1, Nfkbia, Rela]	3	nucleotide-binding oligomerization domain containing 2 signaling pathway	0.0357
1902017	[Hap1, Mapk15, Septin9]	3	regulation of cilium assembly	0.0272
1903035	[Anxa5, Fgg, Hmgcr, Phldb2, Pros1, Serpinf2]	6	negative regulation of response to wounding	0.0171

CTR vs HFD+beer

ID GO:	Associated Genes Found	Nr. Genes	Term	PValue
1952	[Enpp2, Lrp1, Mmp14, Pik3r1, Rin2, Smad3]	6	regulation of cell-matrix adhesion	0.0205
1953	[Enpp2, Lrp1, Mmp14, Pik3r1]	4	negative regulation of cell-matrix adhesion	0.0081
1959	[Otop1, Ptprf, Sh2b3, Usp18, Usp29, Wnt5a]	6	regulation of cytokine-mediated signaling pathway	0.0283
6638	[Esr1, Fgf21, Mgll, Mogat2, Plce1, Scd1, Tcf7l2, Tom1l2]	8	neutral lipid metabolic process	0.0008
6639	[Esr1, Fgf21, Mgll, Mogat2, Plce1, Scd1, Tcf7l2, Tom1l2]	8	acylglycerol metabolic process	0.0008
6641	[Esr1, Fgf21, Mgll, Mogat2, Scd1, Tcf7l2, Tom1l2]	7	triglyceride metabolic process	0.0011
7044	[Dst, Enpp2, Lrp1, Mmp14, Pxn, Smad3]	6	cell-substrate junction assembly	0.0039
8347	[Lrp1, Mmp14, Syne2]	3	glial cell migration	0.0400
10717	[Eng, Gcnt2, Smad3, Tcf7l2, Tiam1]	5	regulation of epithelial to mesenchymal transition	0.0334
10718	[Eng, Gcnt2, Smad3, Tcf7l2]	4	positive regulation of epithelial to mesenchymal transition	0.0253
10769	[Camk2b, Enpp2, Fbxo31, Ptprf, Triobp, Xlr4b]	6	regulation of cell morphogenesis involved in differentiation	0.0143
10770	[Camk2b, Enpp2, Fbxo31, Ptprf, Triobp, Xlr4b]	6	positive regulation of cell morphogenesis involved in differentiation	0.0064
10812	[Enpp2, Gcnt2, Lrp1, Mmp14, Pik3r1]	5	negative regulation of cell-substrate adhesion	0.0047
30193	[F2, F2r, Sh2b3]	3	regulation of blood coagulation	0.0276
35904	[Eng, Lrp1, Pkd2]	3	aorta development	0.0400
42058	[Bcar3, Esr1, Ptprf]	3	regulation of epidermal growth factor receptor signaling pathway	0.0149
45599	[Ncor2, Smad3, Tcf7l2, Wnt5a]	4	negative regulation of fat cell differentiation	0.0343
46460	[Mogat2, Plce1, Tcf7l2, Tom1l2]	4	neutral lipid biosynthetic process	0.0328
46463	[Mogat2, Plce1, Tcf7l2, Tom1l2]	4	acylglycerol biosynthetic process	0.0328
46503	[Enpp2, Fgf21, Mgll]	3	glycerolipid catabolic process	0.0400
48041	[Enpp2, Lrp1, Mmp14, Pxn, Smad3]	5	focal adhesion assembly	0.0196
50775	[Camk2b, Fbxo31, Ptprf, Xlr4b]	4	positive regulation of dendrite morphogenesis	0.0433
90101	[Eng, Htra3, Lrp1, Ppara, Tcf7l2, Wnt5a]	6	negative regulation of transmembrane receptor protein serine/threonine kinase signaling pathway	0.0299
90207	[Esr1, Fgf21, Tcf7l2, Tom1l2]	4	regulation of triglyceride metabolic process	0.0267

150115	[Dst, Enpp2, Lrp1, Mmp14, Pik3r1, Pxn, Smad3]	7	cell-substrate junction organization	0.0005
150116	[Enpp2, Lrp1, Mmp14, Pik3r1, Smad3]	5	regulation of cell-substrate junction organization	0.0075
1900046	[F2, F2r, Sh2b3]	3	regulation of hemostasis	0.0149
1901890	[Cldn3, Enpp2, Nphp4, Pik3r1, Smad3, Xlr4b]	6	positive regulation of cell junction assembly	0.0147
2000107	[Blm, Dock8, Irs2]	3	negative regulation of leukocyte apoptotic process	0.0276
**CTR vs CTR+beer**				
**ID GO:**	**Associated Genes Found**	**Nr. Genes**	**Term**	**PValue**
2076	[Runx2, Smad3, Tro]	3	osteoblast development	0.0297
2293	[Il4ra, Lef1, Prkcz]	3	alpha-beta T cell differentiation involved in immune response	0.0206
2294	[Il4ra, Lef1, Prkcz]	3	CD4-positive, alpha-beta T cell differentiation involved in immune response	0.0398
3143	[Pkd2, Smad3, Wnt5a]	3	embryonic heart tube morphogenesis	0.0398
5245	[Cabp1, Cacna1c, Cacna1e, Gm37013, Pkd2]	5	voltage-gated calcium channel activity	0.0392
32332	[Rela, Runx2, Smad3, Zbtb16]	4	positive regulation of chondrocyte differentiation	0.0018
42308	[Apod, Cabp1, Hnf4a]	3	negative regulation of protein import into nucleus	0.0261
42532	[Hnf4a, Sh2b3, Socs1]	3	negative regulation of tyrosine phosphorylation of STAT protein	0.0077
46426	[Hnf4a, Sh2b3, Socs1, Socs2]	4	negative regulation of receptor signaling pathway via JAK-STAT	0.0021
46627	[Prkcz, Rela, Socs1, Tns2]	4	negative regulation of insulin receptor signaling pathway	0.0170
46632	[Blm, Il4ra, Lef1, Prkcz, Socs1, Zbtb16]	6	alpha-beta T cell differentiation	0.0361
46634	[Blm, Cd44, Il4ra, Prkcz, Socs1, Zbtb16]	6	regulation of alpha-beta T cell activation	0.0212
46635	[Blm, Il4ra, Prkcz, Socs1, Zbtb16]	5	positive regulation of alpha-beta T cell activation	0.0208
46638	[Il4ra, Prkcz, Socs1, Zbtb16]	4	positive regulation of alpha-beta T cell differentiation	0.0388
48339	[Lef1, Smad3, Wnt5a]	3	paraxial mesoderm development	0.0465
48340	[Lef1, Smad3, Wnt5a]	3	paraxial mesoderm morphogenesis	0.0096
61035	[Rela, Runx2, Smad3, Wnt5a, Zbtb16]	5	regulation of cartilage development	0.0214
61036	[Rela, Runx2, Smad3, Wnt5a, Zbtb16]	5	positive regulation of cartilage development	0.0005
61371	[Pkd2, Smad3, Wnt5a]	3	determination of heart left/right asymmetry	0.0398
1900077	[Prkcz, Rela, Socs1, Tns2]	4	negative regulation of cellular response to insulin stimulus	0.0247
1903307	[Gm37013, Il4ra, Slc4a8]	3	positive regulation of regulated secretory pathway	0.0206
1904893	[Hnf4a, Sh2b3, Socs1, Socs2]	4	negative regulation of receptor signaling pathway via STAT	0.0034
**HFD vs HFD+beer**				
**ID GO:**	**Associated Genes Found**	**Nr. Genes**	**Term**	**PValue**
3205	[Ccn1, Heg1, Kif7, Lrp1, Ppp1r13l, Rbp4, Smad7, Srf, Tpm1]	9	cardiac chamber development	0.0167
22602	[Esr1, Inhba, Iqschfp, Map2k6, Runx1]	5	ovulation cycle process	0.0490
42698	[Esr1, Inhba, Iqschfp, Map2k6, Ncor2, Runx1]	6	ovulation cycle	0.0341
44843	[Bcl7a, Camk2b, Fbxo31, Inhba, Kcna5, Mblac1, Myo16, Paf1, Plcg2, Rptor, Tcf7l1]	11	cell cycle G1/S phase transition	0.0063
46637	[Hlx, Runx1, Smad7]	3	regulation of alpha-beta T cell differentiation	0.0384
48168	[Camk2b, Rab3a, Shank3]	3	regulation of neuronal synaptic plasticity	0.0384

50891	[Ncor2, Pnpla1, Srf]	3	multicellular organismal water homeostasis	0.0384
60044	[Jarid2, Meis1, Rbp4]	3	negative regulation of cardiac muscle cell proliferation	0.0499
60976	[Kif7, Lrp1, Srf]	3	coronary vasculature development	0.0384
71772	[Ccn1, Col2a1, Kcp, Lef1, Numa1, Skil, Smad7, Tmprss6]	8	response to BMP	0.0355
71773	[Ccn1, Col2a1, Kcp, Lef1, Numa1, Skil, Smad7, Tmprss6]	8	cellular response to BMP stimulus	0.0355
1900046	[F2r, Fga, Tmprss6]	3	regulation of hemostasis	0.0384
1902806	[Bcl7a, Fbxo31, Inhba, Kcna5, Mblac1, Myo16, Paf1, Plcg2, Rptor, Tcf7l1]	10	regulation of cell cycle G1/S phase transition	0.0027
1902808	[Kcna5, Mblac1, Paf1, Plcg2, Rptor]	5	positive regulation of cell cycle G1/S phase transition	0.0306
2000045	[Bcl7a, Fbxo31, Inhba, Kcna5, Mblac1, Myo16, Rptor, Tcf7l1]	8	regulation of G1/S transition of mitotic cell cycle	0.0292
2000144	[Esr1, Srf, Xpa]	3	positive regulation of DNA-templated transcription, initiation	0.0384

Table S9. List of DMCs presenting longest CpG stretches, consecutive cytosine ≤ 2000bp above five, for all four comparisons.

**CTR*vs*HFD**		**CTR*vs*HFD+beer**		**HFD*vs*HFD+beer**		**CTR*vs*CTR+beer**	
**Feature**	**near DMCs**	**Feature**	**near DMCs**	**Feature**	**near DMCs**	**Feature**	**near DMCs**
Gm26917	91	Gm26917	20	Socs2	14	Gm26917	192
Klf15	14	Arhgap26	10	Commd1	10	Cmss1	31
Arhgap26	8	Snd1	9	Tns2	10	Commd1	13
Gm36876	6	Camk2b	8	Obsl1	9	Gm36876	9
Nfkbil1	6	Bcor	8	Cit	8	Gm53756	9
Paf1	5	Eng	7	Camk2b	8	Hivep2	8
Tob1	5	Camkk2	7	Cmss1	8	Agap2	8
Tcf4	5	Scd1	7	B3galt4	8	Fcgr3	7
Scd1	5	Gm17359	6	Clstn1	7	Skil	7
		Arl6ip4	6	Nmral1	7	Rnf186	7
		2610035D17Rik	6	Nadk	6	Bcl9l	7
		Gm9805	6	Snd1	6	Nrros	7
		Xlr4a	6	Gm32425	6	Rbp4	7
		Dst	5	Kcnk4	6	Arhgap21	6
		Hc	5	Btbd3	5	B3gnt3	6
		Gm34911	5	Gm17359	5	Fbxo31	6
		Gm2065	5	Sh2b3	5	Gm35197	6
		Lrrc3	5	Camkk2	5	Fignl2	6
		Gcnt2	5	Gm34911	5	Brd2	6
				Septin9	5	Mid1	6
				Xdh	5	Fzd7	5
				Rela	5	Mllt10	5
				Cyp26a1	5	Hnrnpdl	5
						Mapk15	5

Pced1b	5
Runx1	5

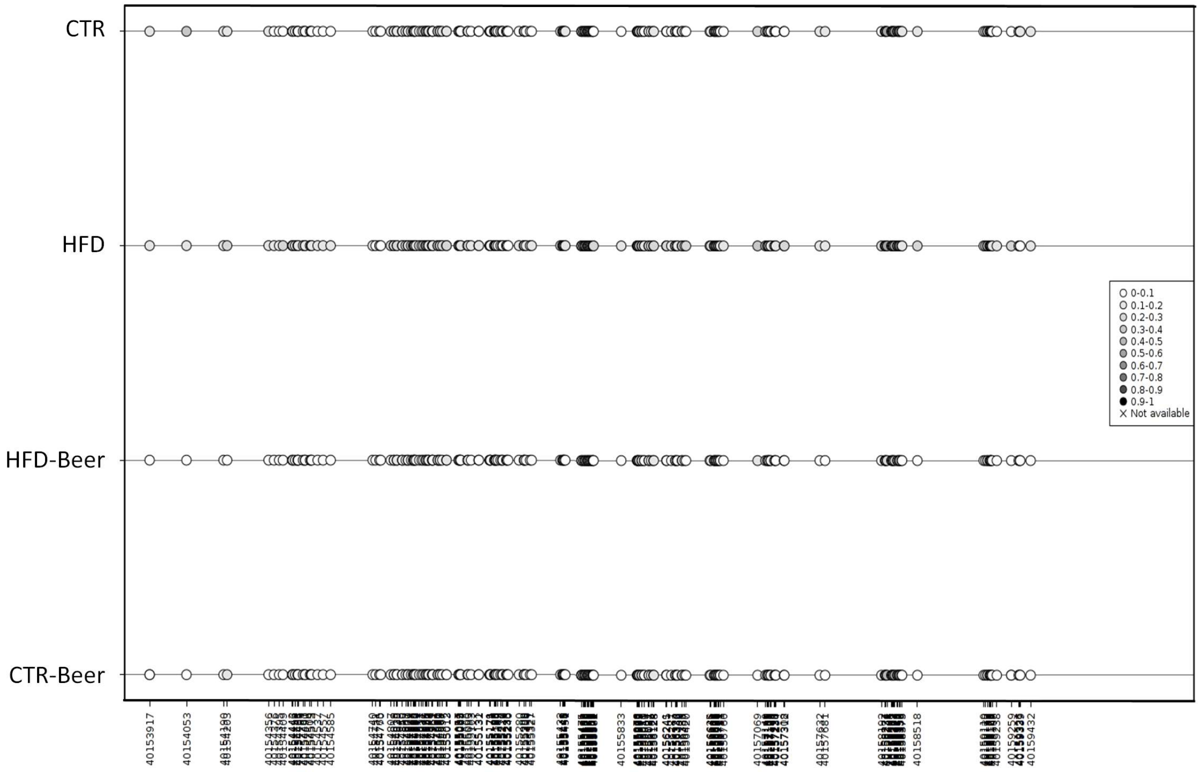

Figure S3. Representation of average level of CpG methylation for mice subjected to the four different treatments for Gm26917 gene presenting a high number of close differentially methylated stretches.

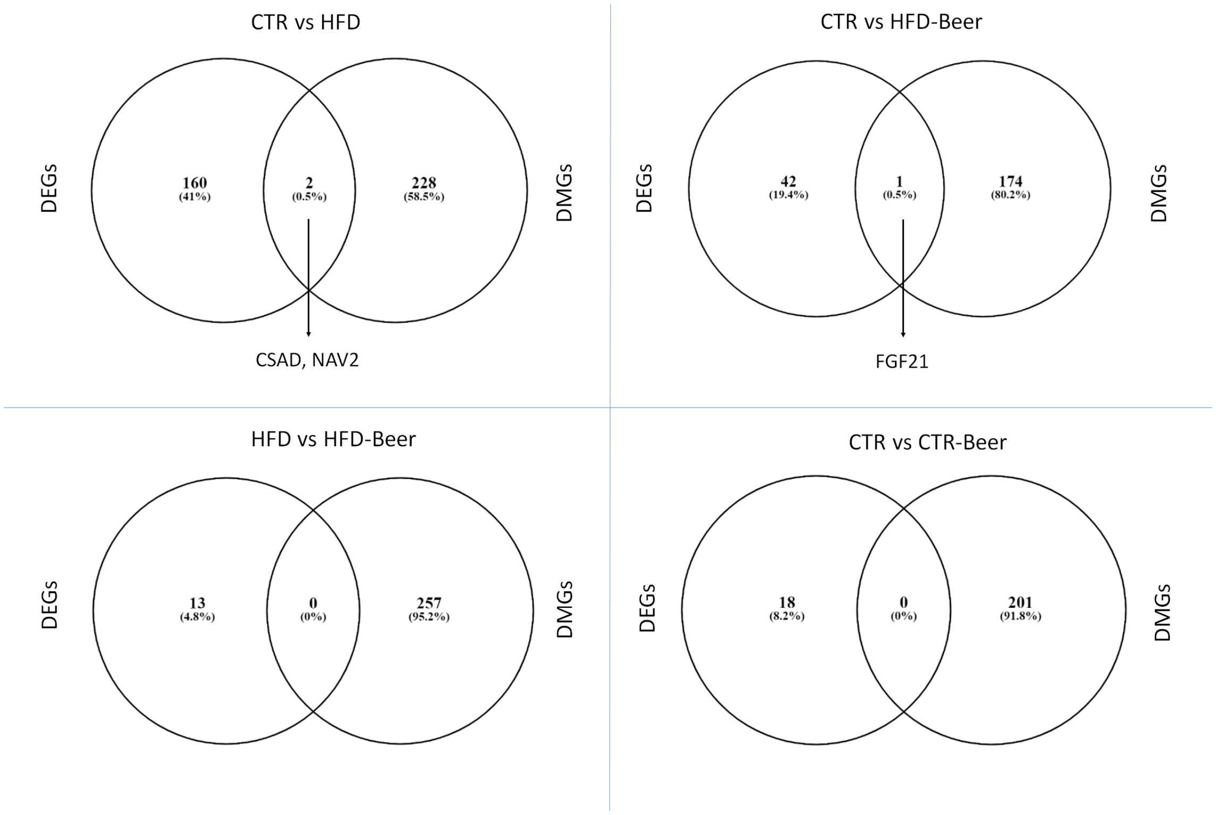

Figure S4. Venn Diagram of shared differentially expressed (DEGs) and differentially methylated (DMGs) genes found between RNA-Seq and RRBBs dataset, for different comparison: CTR vs HFD, CTR vs HFD-Beer, HFD vs HFD-Beer and CTR vs CTR-Beer.
